# Blockade of the pro‐fibrotic reaction mediated by the miR‐143/‐145 cluster enhances the responses to targeted therapy in melanoma

**DOI:** 10.15252/emmm.202115295

**Published:** 2022-02-14

**Authors:** Serena Diazzi, Alberto Baeri, Julien Fassy, Margaux Lecacheur, Oskar Marin‐Bejar, Christophe A Girard, Lauren Lefevre, Caroline Lacoux, Marie Irondelle, Carine Mounier, Marin Truchi, Marie Couralet, Mickael Ohanna, Alexandrine Carminati, Ilona Berestjuk, Frederic Larbret, David Gilot, Georges Vassaux, Jean‐Christophe Marine, Marcel Deckert, Bernard Mari, Sophie Tartare‐Deckert

**Affiliations:** ^1^ Université Côte d'Azur INSERM C3M Nice France; ^2^ Université Côte d'Azur CNRS Institut de Pharmacologie Moléculaire et Cellulaire (IPMC) Sophia Antipolis France; ^3^ Equipe labellisée Ligue Contre le Cancer Nice France; ^4^ Laboratory For Molecular Cancer Biology VIB Center for Cancer Biology VIB Leuven Belgium; ^5^ Department of Oncology KU Leuven Leuven Belgium; ^6^ CYU Université, ERRMECe (EA1391) Neuville‐sur‐Oise France; ^7^ INSERM U1242 University of Rennes Rennes France; ^8^ FHU‐OncoAge Nice France

**Keywords:** fibrosis, MAPK inhibitors, melanoma, microRNA, nintedanib, Cancer, Skin

## Abstract

Lineage dedifferentiation toward a mesenchymal‐like state displaying myofibroblast and fibrotic features is a common mechanism of adaptive and acquired resistance to targeted therapy in melanoma. Here, we show that the anti‐fibrotic drug nintedanib is active to normalize the fibrous ECM network, enhance the efficacy of MAPK‐targeted therapy, and delay tumor relapse in a preclinical model of melanoma. Acquisition of this resistant phenotype and its reversion by nintedanib pointed to miR‐143/‐145 pro‐fibrotic cluster as a driver of this mesenchymal‐like phenotype. Upregulation of the miR‐143/‐145 cluster under BRAFi/MAPKi therapy was observed in melanoma cells *in vitro* and *in vivo* and was associated with an invasive/undifferentiated profile. The 2 mature miRNAs generated from this cluster, miR‐143‐3p and miR‐145‐5p, collaborated to mediate transition toward a drug‐resistant undifferentiated mesenchymal‐like state by targeting Fascin actin‐bundling protein 1 (FSCN1), modulating the dynamic crosstalk between the actin cytoskeleton and the ECM through the regulation of focal adhesion dynamics and mechanotransduction pathways. Our study brings insights into a novel miRNA‐mediated regulatory network that contributes to non‐genetic adaptive drug resistance and provides proof of principle that preventing MAPKi‐induced pro‐fibrotic stromal response is a viable therapeutic opportunity for patients on targeted therapy.

## Graphical abstract

This study identifies a critical miRNA‐mediated regulatory axis controlling melanoma cell phenotypic adaptation and resistance to MAPK inhibitors (MAPKi) and targetable by the anti‐fibrotic drug Nintedanib.

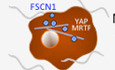

**The paper explained**

**Problem**

Despite recent improvements in targeting metastatic melanoma, resistance to inhibition of the BRAFV600 oncogenic pathway occurs in most patients treated with MAPK‐inhibiting drugs. Melanoma cells adopt various means to evade therapy, including transcriptional reprogramming leading to phenotypic dedifferentiation and acquisition of mesenchymal and pro‐fibrotic features. This state of cellular resistance is highly invasive and displays an increased ability to produce and remodel the extracellular matrix (ECM), creating a drug‐tolerant microenvironment. However, the molecular networks that define this pro‐fibrotic cellular behavior and promote resistance are still unclear.

**Results**

We show that the anti‐fibrotic drug nintedanib prevents the fibrotic reaction and improves MAPK‐targeting therapy efficacy, retarding the onset of resistance in a mouse melanoma model. Expression screening and mechanistic studies identified the pro‐fibrotic miR‐143/‐145 cluster as a driver of nintedanib‐sensitive mesenchymal resistant phenotype. Using a combination of gain‐ and loss‐of‐function approaches, we dissected the molecular and cellular processes regulated by these FibromiRs and demonstrate that during drug adaptation, melanoma cells upregulate the miRNA cluster, which drives a phenotypic switch toward a dedifferentiated therapy‐resistant state. The miR‐143/‐145 cluster also induces ECM production and promotes cell migration and invasion through the activation of focal adhesion dynamics and mechanotransduction pathways. Finally, Fascin actin‐bundling protein 1 (FSCN1) was identified as a key functional target of miR‐143‐3p and miR‐145‐5p for the acquisition of the pro‐fibrotic therapy‐resistant phenotype.

**Impact**

Our study highlights non‐genetic mechanisms of therapeutic resistance in melanoma and deciphers a regulatory cascade involving the miR‐143/‐145/FSCN1 pro‐fibrotic axis in the acquisition of a therapy‐resistant cellular state. It also provides a scientific rationale for designing clinical trials with nintedanib and potentially other anti‐fibrotic agents to overcome resistance in patients with BRAF‐mutated melanoma. Finally, our findings might have implications for other MAPK‐driven cancers and fibrosis‐related diseases.

## Introduction

Because of its high mutational burden, metastasis propensity, and resistance to treatment, cutaneous melanoma is one of the most aggressive human cancers and the deadliest form of skin cancer (Shain & Bastian, 2016). Melanoma is a non‐epithelial tumor that originates from neural crest‐derived and pigment‐producing melanocytes in the skin. Genetic alterations in the *BRAF*, *NRAS*, or *NF1* genes define melanoma subtypes and lead to the MAPK pathway hyperactivation (Flaherty *et al*, 2012; Cancer Genome Atlas, 2015). Current therapeutic options for BRAFV600E/K metastatic melanoma include MAPK‐targeted therapies, which show remarkable efficacy during the first months of treatment (Chapman *et al*, 2011; Robert *et al*, 2019). However, the majority of patients treated with a combination of BRAF inhibitor (BRAFi) and MEK inhibitor (MEKi) inevitably relapse within months (Long *et al*, 2017). Genetic mechanisms of resistance cannot singly explain the acquisition of therapy resistance in melanoma, and non‐genetic heterogeneity actively participates in drug tolerance (Rambow *et al*, 2019; Marine *et al*, 2020). Extensive studies have been carried out to dissect the non‐mutational mechanisms of resistance (Rambow *et al*, 2018; Tsoi *et al*, 2018). Genetic and non‐genetic mechanisms of resistance are frequently linked and not mutually exclusive (Marine *et al*, 2020). Non‐genetic resistance is due to the intrinsic melanoma cell phenotypic plasticity, i.e., ability to undergo transcriptional and epigenetic reprogramming in response to environmental challenges or upon therapy (Arozarena & Wellbrock, 2019). These adaptive mechanisms exploit the developmental plasticity of melanoma cells and often result in an undifferentiated state characterized by upregulation of receptor tyrosine kinases (RTK) such as PDGFRβ or AXL, downregulation of melanocyte differentiation transcription factors MITF and SOX10 (Sun *et al*, 2014), and acquisition of mesenchymal and invasive features (Nazarian *et al*, 2010; Villanueva *et al*, 2010; Girotti *et al*, 2013; Muller *et al*, 2014; Fallahi‐Sichani *et al*, 2017; Rambow *et al*, 2018; Tsoi *et al*, 2018; Rathore *et al*, 2019).

Tumors are shaped dynamically by reciprocal crosstalk between cancer cells and the extracellular matrix (ECM) through cellular–ECM interactions and stromal matrix remodeling. Recent findings indicated that elevated ECM production and remodeling contribute to adaptive and acquired resistance to BRAFi therapy by conferring a drug‐protective niche to melanoma cells (Fedorenko *et al*, 2016; Titz *et al*, 2016; Girard *et al*, 2020; Marusak *et al*, 2020). Moreover, we recently reported that undifferentiated mesenchymal‐like BRAFi‐resistant cells exhibit myofibroblast/cancer‐associated fibroblast (CAF)‐like features leading to pro‐fibrotic ECM reprogramming *in vitro* and *in vivo* (Diazzi *et al*, 2020; Girard *et al*, 2020). Cell‐autonomous ECM deposition and remodeling abilities adopted by melanoma cells after MAPKi treatment result in cross‐linked collagen matrix and tumor stiffening fostering a feedforward loop dependent on the mechanotransducers YAP and MRTFA and leading to therapy resistance (Girard *et al*, 2020). Thus, this pro‐fibrotic‐like response, typical of the early adaptation and acquired resistance to MAPK inhibition, provides a therapeutic escape route through the activation of alternative survival pathways mediated by cell‐matrix communications. However, the signaling networks underlying the acquisition of this undifferentiated, mesenchymal‐like melanoma cell state and drug‐resistant behavior remain unclear.

We reasoned that therapeutic approaches aimed at preventing this targeted therapy‐induced abnormal pro‐fibrotic reaction could represent rationale combination strategies to normalize the fibrous stroma and overcome non‐genetic resistance in BRAFV600E‐mutated melanomas. We show here that the anti‐fibrotic drug nintedanib (BIBF1120, Ofev^®^) improves the response of the BRAFi/MEKi‐targeted therapy in a preclinical model of melanoma and in BRAF‐mutated cell lines by preventing MAPKi‐induced lineage dedifferentiation, ECM reprogramming, and mesenchymal traits. We also identified the master regulator associated with the acquisition of this pro‐fibrotic and dedifferentiation program, pointing the miR‐143/‐145 cluster as a driver of the phenotype switching to a drug‐resistant mesenchymal‐like cell state.

## Results

### Nintedanib/BIBF1120 prevents MAPKi‐induced pro‐fibrotic‐like response, enhances targeted therapy efficiency, and delays tumor relapse

**Figure 1:**
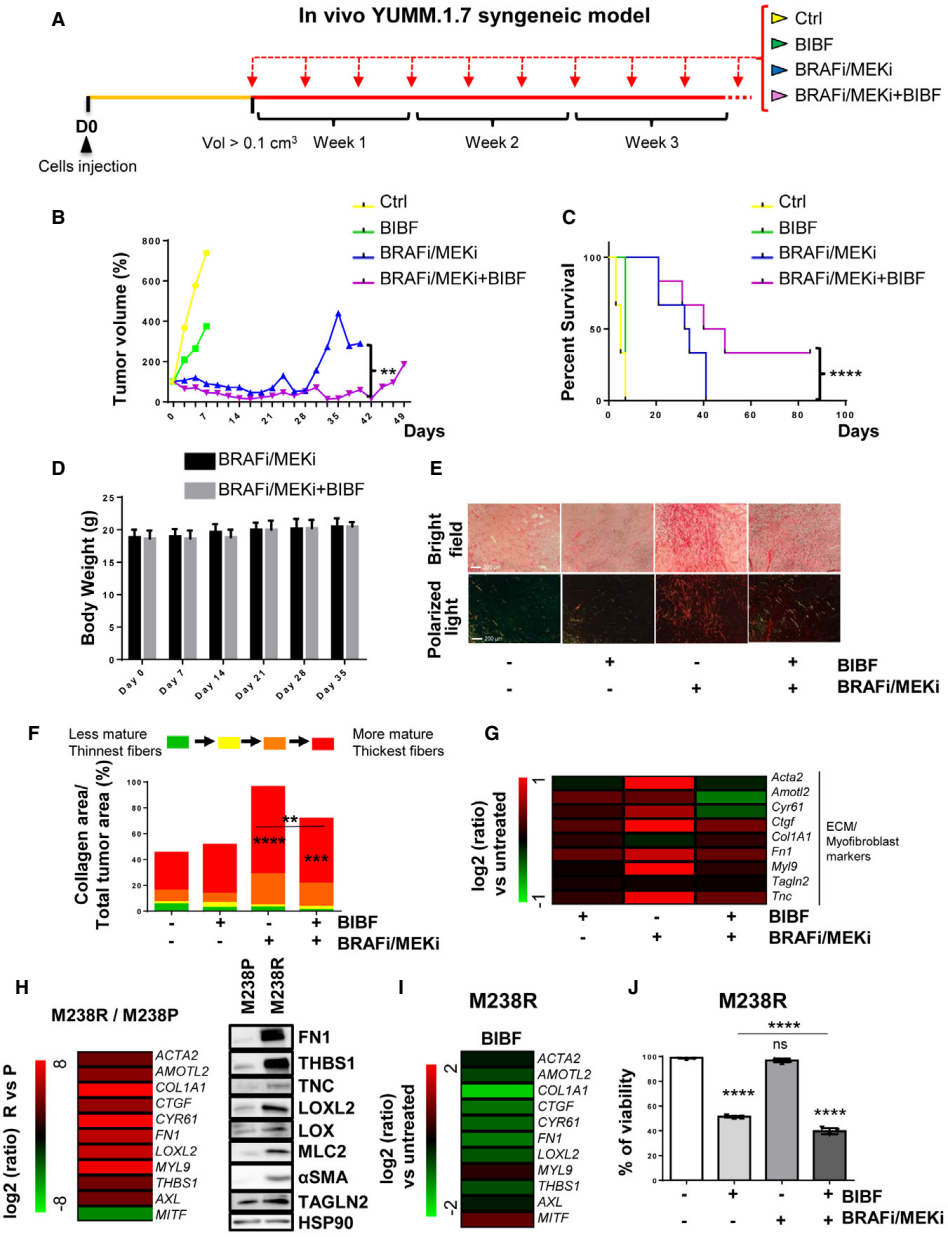
Nintedanib/BIBF1120 prevents MAPKi‐induced ECM remodeling, decreases resistance to targeted therapy, and delays tumor relapse A–GMouse YUMM1.7 melanoma cells were subcutaneously inoculated into C57BL/6 mice, and when tumors reached 100 mm^3^, mice were treated with vehicle (Ctrl), nintedanib/BIBF1120 (BIBF), MAPKi (BRAFi, vemurafenib and MEKi, trametinib), or BRAFi/MEKi plus BIBF (*n* = 6). (B) Representative median graphics showing tumor growth following treatment (*n* = 6). Two‐way ANOVA was used for statistical analysis. **P ≤ 0.01. (C) Kaplan–Meier survival curves of mice treated with the indicated therapies (*n* = 6). The log rank (Mantel–Cox) statistical test was used for MAPKi vs MAPKi/BIBF1120. ****P ≤ 0.0001. (D) Mouse body weight was measured at the indicated times. Data shown are mean ± SD (*n* = 6). (E, F) Tumor sections were stained with picrosirius red and imaged under polarized light. (E) Representative image of collagen fiber network in tumors from mice under the different treatments. Scale bar 200 μm. (F) Quantification of collagen fiber thickness (*n* = 6 for control, BIBF, and BRAFi/MEKi groups and *n* = 5 for BRAFi/MEKi + BIBF group). Two‐way ANOVA statistical test was used for statistical analysis of mature collagen fiber thickness quantification. **P ≤ 0.01, ***P ≤ 0.001, and ****P ≤ 0.0001. Significance was calculated against the control group. Statistical significance of BIBF vs BIBF + BRAFi/MEKi was also calculated. (G) Heatmap showing the differential expression of ECM and myofibroblast/CAF genes in mice treated with MAPK‐targeted therapies with or without BIBF compared to control mice (log_2_ ratio, *n* = 5).H–JHuman M238R cells and/or parental M238P cells were analyzed for different parameters. (H) Heatmap and Western Blot showing the expression of ECM, myofibroblast/CAF and phenotype switch markers in M238R compared to M238P cells. Heatmap represents the mean of expression of 3 independent experiments by RT‐qPCR. (I) Heatmap showing the expression of ECM, myofibroblast/CAF and phenotype switch markers in M238R treated with BIBF (2 µM, 72 h) or vehicle alone by RT‐qPCR (*n* = 3). (J) Crystal violet viability assay of M238R cells treated with BRAFi/MEKi (BRAFi, Vemurafenib and MEKi, Trametinib) (1 µM), BIBF (2 μM) or with BRAFi/MEKi (1 μM) plus BIBF (2 μM) for 72 h. Paired Student *t*‐test was used for statistical analysis. ****P ≤ 0.0001. Significance was calculated against the control group. Statistical significance of BIBF vs BIBF + BRAFi/MEKi was also calculated. Data is represented as mean ± SD from a triplicate representative of 3 independent experiments. Source data are available online for this figure.

**Figure EV1:**
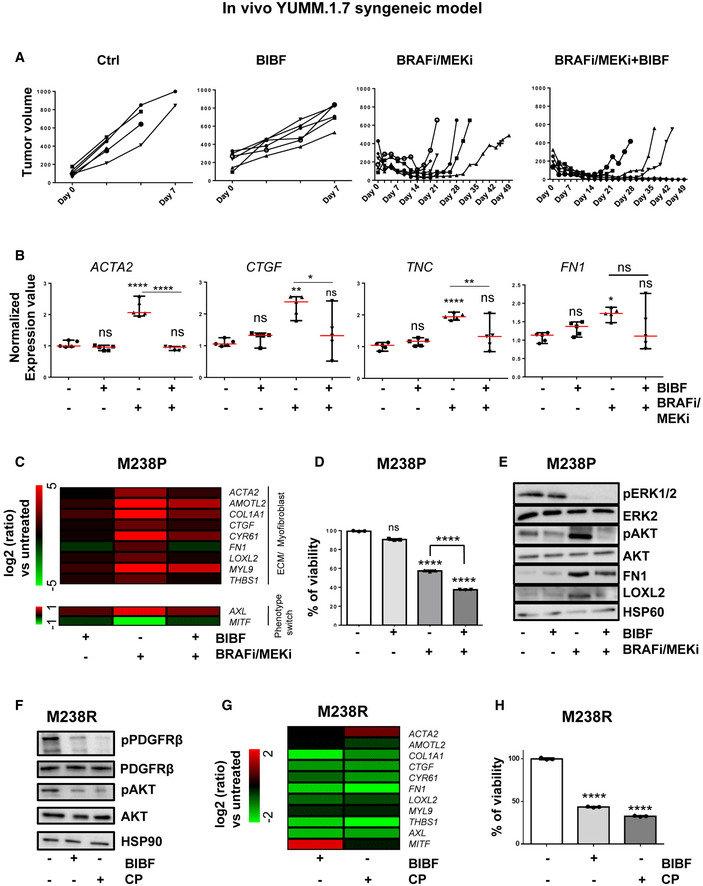
Administration of nintedanib/BIBF1120 resensitizes melanoma cells to MAPK‐targeted therapies, delays tumor relapse, and normalizes MAPKi‐induced ECM remodeling and miR‐143/‐145 expression A, BYUMM1.7 cells were subcutaneously inoculated into C57BL/6 mice, and when tumors reached 100 mm^3^, mice were treated with the indicated therapies. (A) Individual graphics showing tumor growth following treatment. (B) Normalized expression of myofibroblast/CAF and ECM‐related genes assessed by RT‐qPCR in individual tumors treated as indicated. Data are represented as median with range (*n* = 5). One‐way ANOVA was used for statistical analysis. *P ≤ 0.05, **P ≤ 0.01, and ****P ≤ 0.0001. Significance was calculated against the control group. Statistical significance of BRAFi/MEKi vs BRAFi/MEKi + BIBF was also calculated.C–EHuman M238P cells were treated with BRAFi (vemurafenib) + MEKi (trametinib; 1 μM), BIBF1120 (2 µM) or BRAFI + MEKi (1 µM) plus BIBF (2 µM) for 72 h. (C) Heatmap showing the expression of ECM, myofibroblast/CAF markers, and phenotype switch markers by RT‐qPCR (*n* = 3). (D) Crystal violet viability assay of M238P cells treated with MAPK‐targeted therapies as above. Paired Student's *t*‐test was used for statistical analysis. ****P ≤ 0.0001. Data are represented as mean ± SD from a triplicate representative of three independent experiments. (E) Western blot showing the expression of ECM, myofibroblast/CAF markers, and activation levels of signaling pathways (AKT and ERK1/2) in the different conditions.F–HHuman M238R cells were treated with BIBF1120 (2 µM) or with CP673451 (2 µM) for 72 h. (F) Western blot showing activation levels of signaling pathways (PDGFR and AKT) in the different conditions. (G) Heatmap showing the expression of ECM, myofibroblast/CAF markers, and phenotype switch markers by RT‐qPCR in M238R cells treated with the indicated inhibitors (*n* = 3). (H) Crystal violet viability assay of M238R cells treated with the indicated inhibitors. Paired Student's *t*‐test was used for statistical analysis. ****P ≤ 0.0001. Data are represented as mean ± SD from a triplicate representative of three independent experiments.

In order to limit ECM reprogramming and collagen remodeling associated with therapy resistance and relapse in melanoma, we tested the effect of the anti‐fibrotic drug nintedanib (BIBF1120), a triple inhibitor of PDGFR, VEGFR, and FGFR used to treat idiopathic pulmonary fibrosis (IPF) in combination with BRAFi/MEKi in a syngeneic model of transplanted murine YUMM1.7 Braf‐mutant melanoma (Meeth *et al*, 2016). YUMM1.7 cells were subcutaneously injected, and tumors were treated with vehicle, BIBF1120 alone, a combination of BRAFi plus MEKi, or the triple combination (Fig 1A). BIBF1120 did not display any anti‐melanoma effect when administered alone, slightly slowing down tumor growth but not triggering tumor volume decrease. Administration of the BRAFi/MEKi initially reduced tumor growth, but after three weeks of treatment, tumor growth resumed and 100% of tumors relapsed. Importantly, combination of MAPK‐targeted therapies and BIBF1120 significantly delayed relapse and led to complete remission in 33% of mice (2 out of 6; Figs 1B and C, and EV1A). Overall, the combined treatment significantly improved mouse survival (Fig 1C) without body weight loss or sign of toxicity throughout the study (Fig 1D). As previously described in melanoma xenograft models (Girard *et al*, 2020), an extensive deposition of collagens and increased expression of ECM remodeling and myofibroblast markers were observed in YUMM1.7 tumors treated with the combination of BRAFi and MEKi as revealed by picrosirius red staining of collagen fibers and qPCR analysis of typical molecular markers of tumor fibrosis. This response was significantly reduced by the co‐administration of BIBF1120 (Figs 1E–G and EV1B). Thus, combination of targeted therapy with the anti‐fibrotic drug nintedanib prevents the appearance of a pro‐fibrotic matrix observed upon MAPK‐targeted therapy exposure and significantly delays the onset of resistance *in vivo*.

We next examined the impact of nintedanib on ECM reprogramming and cell phenotype switching in the context of early adaptation and resistance to MAPK‐targeted therapy in human BRAFV600E‐mutated melanoma M238P cells. BIBF1120 strongly attenuated targeted drug‐induced ECM/myofibroblast‐related signatures, prevented the undifferentiated AXLhigh–MITFlow phenotype switch (Fig EV1C) and potentiated the effect of the BRAFi/MEKi cocktail on M238P cell viability (Fig EV1D). The efficacy of the described treatment to reduce upregulation of fibronectin (FN1) and LOXL2 expression was confirmed at protein levels by Western blot analysis (Fig EV1E). Of note, a strong activation of AKT induced by the BRAFi/MEKi cocktail was fully inhibited by BIBF1120, suggesting that the anti‐fibrotic drug is able to counteract the rewiring of alternative survival pathway observed upon MAPK oncogenic pathway inhibition (Fig EV1E) (Nazarian *et al*, 2010).

We finally evaluated the effect of BIBF1120 on the undifferentiated mesenchymal‐like resistant M238R cells obtained through chronic exposure of the M238P cells to the BRAFi vemurafenib (Nazarian *et al*, 2010) and that displayed cross‐resistance to MEKi (Atefi *et al*, 2011). We recently reported that this RTK‐driven resistant cell line exhibits low expression of the differentiation factor MITF and high AXL levels and displays a strong myofibroblast‐like phenotype with expression of classical ECM and contractile markers such as smooth muscle actin‐α (αSMA) and myosin light chain 2 (MLC2), as well as ECM remodeling activities compared with parental M238P cells (Fig 1H) (Girard *et al*, 2020). BIBF1120 was able to attenuate melanoma‐undifferentiated state markers and expression of ECM and myofibroblast/CAF‐related signature (Fig 1I), but also significantly decreased cell viability and resistance to BRAFi (Fig 1J). To address the specific contribution of PDGFRβ inhibition in nintedanib/BIBF1120 effects, we compared the effect of the selective PDGFR inhibitor CP673451 with BIBF1120 in M238R‐resistant cells. The two inhibitors showed similar efficiency in causing a strong decrease in phospho‐PDGFRβ and phospho‐AKT levels (Fig EV1F). However, while selective inhibition of PDGFR attenuated the myofibroblast‐like signature typical of resistant cells (Fig EV1G) and significantly decreased cell viability (Fig EV1H), CP673451 was found less efficient than BIBF1120 in inducing phenotypic switch toward a more differentiated cell state (Fig EV1G). Altogether, these findings indicate that an anti‐fibrotic therapy is able to revert the undifferentiated mesenchymal resistant phenotype and potentiate targeted therapy in human melanoma cells.

### Suppression of MAPKi‐induced resistant pro‐fibrotic phenotype by nintedanib is associated with loss of miR‐143/145 cluster expression

**Figure 2:**
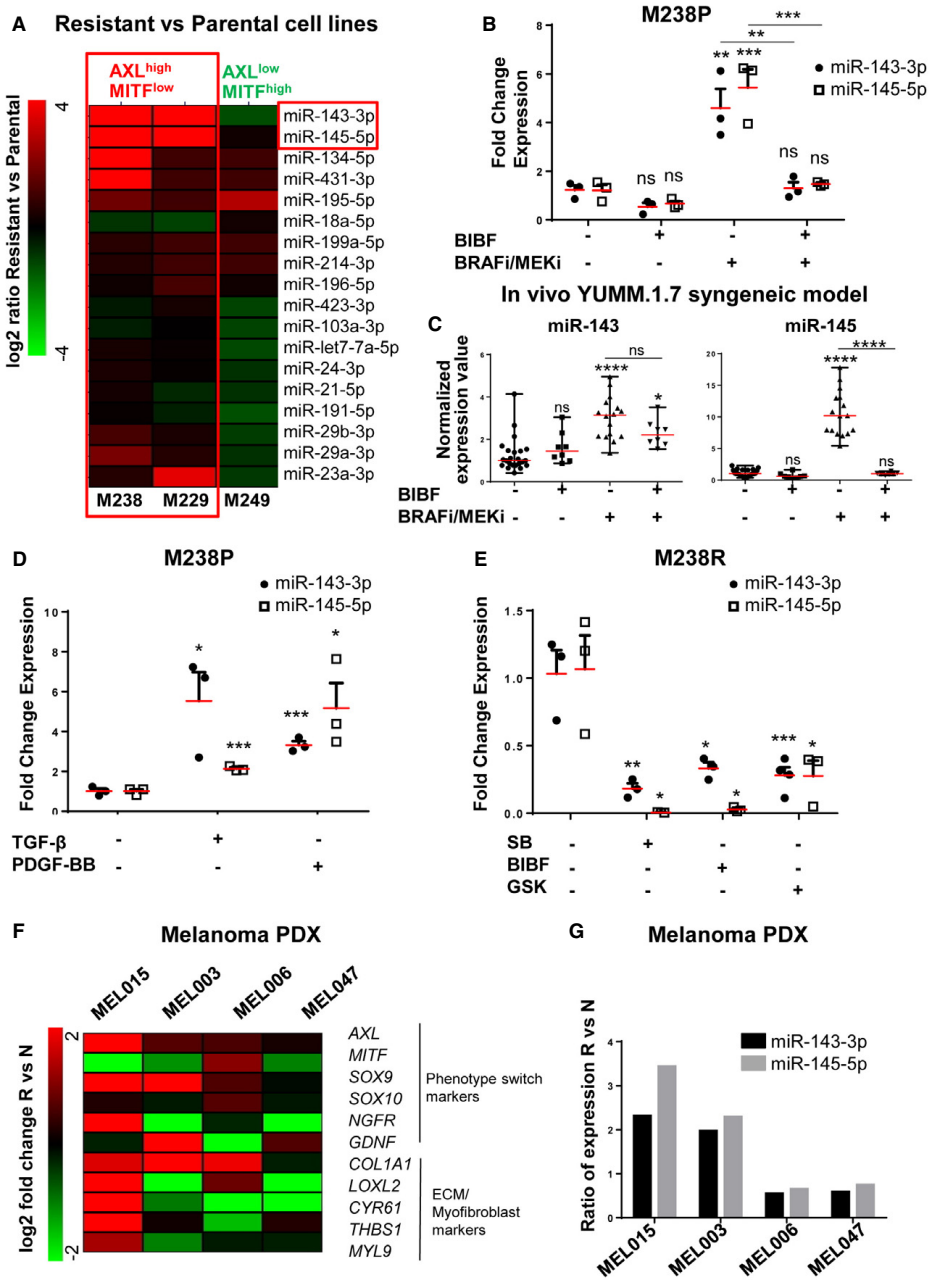
Expression of miR‐143/‐145 is correlated with the undifferentiated mesenchymal‐like MAPKi‐resistant phenotype and is negatively regulated by nintedanib/BIBF1120 AHeatmap showing the differential expression of a selection of miRNAs known as “FibromiRs” in human BRAF^V600E^ mutant melanoma cells sensitive to MAPK‐targeted therapies (M229, M238, M249) and the corresponding BRAFi‐resistant cells. Expression of indicated FibromiRs was evaluated by RT‐qPCR; log_2_ (resistant vs parental). Expression level of AXL and MITF phenotypic markers for each resistant cell line is indicated.BRelative miRNA expression levels were quantified in M238P cells treated for 72 h with BIBF1120 (BIBF, 2 μM), MAPKi (BRAFi, vemurafenib and MEKi, trametinib; 1 µM), or with MAPKi (1 μM) plus BIBF (2 µM) by RT‐qPCR and normalized to miR‐16‐5p. Data are represented as mean ± SEM from a triplicate representative of three independent experiments. One‐way ANOVA was used for statistical analysis. **P ≤ 0.01 and ***P ≤ 0.001. Significance was calculated against the control group. Statistical significance of BRAFi/MEKi vs BRAFi/MEKi + BIBF was also calculated.CExpression of miR‐143‐3p and miR‐145‐5p in control mice and mice treated with the indicated therapies (see legend of Fig 1 for details) was quantified by RT‐qPCR. Data are represented as mean ± SEM from two independent experiments performed on six mice, with two sites of injections. One‐way ANOVA was used for statistical analysis. *P ≤ 0.05 and ****P ≤ 0.0001. Significance was calculated against the control group. Statistical significance of BRAFi/MEKi vs BRAFi/MEKi + BIBF was also calculated.DRelative miRNA expression levels were quantified in M238P cells stimulated for 48 h with TGF‐β (10 ng/ml) or PDGF‐BB (20 ng/ml) by RT‐qPCR and normalized to miR‐16‐5p. Data are represented as mean ± SEM from a triplicate representative of three independent experiments. *P*‐values were calculated using paired Student's *t*‐test. *P ≤ 0.05 and ***P ≤ 0.001.ERelative miRNA expression levels were quantified in M238R cells treated for 48 h with the triple kinase inhibitor nintedanib/BIBF1120 (BIBF, 2 μM), the TGF‐β receptor kinase inhibitor SB431542 (SB, 10 µM), and the pan‐AKT inhibitor GSK690693 (GSK, 10 µM) by RT‐qPCR. Data are represented as mean ± SEM from a triplicate representative of three independent experiments. *P*‐values were calculated using paired Student's *t*‐test. *P ≤ 0.05, **P ≤ 0.01, and ***P ≤ 0.001.F, GPhenotype switch/invasive/ECM markers (F) and relative miRNAs expression levels (G) were quantified in therapy‐naïve (N) and therapy‐resistant (R) PDX samples. The log_2_ fold change or the ratio of the fold change R vs N is shown for each couple of samples. Source data are available online for this figure.

**Figure EV2:**
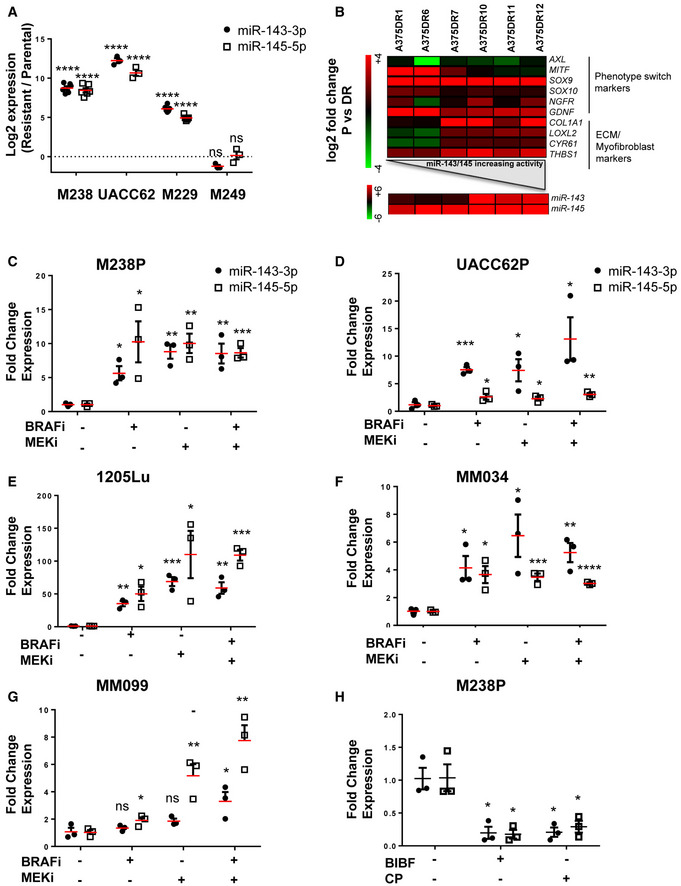
High expression of miR‐143/‐145 is correlated with an undifferentiated/mesenchymal‐like BRAFi‐resistant phenotype in melanoma cells ARelative miRNA expression levels were quantified in parental (P) and paired resistant (R) cells (M238, UACC62, M229, M249) by RT‐qPCR. Log_2_ (R/P) is shown for each cell line. Data are represented as mean ± SE from a triplicate representative of at least three independent experiments. Paired Student's *t*‐test was used for statistical analysis. ****P ≤ 0.0001.BHeatmap showing the expression of ECM, myofibroblast/CAF, and phenotype switch markers, and miRNAs by RT‐qPCR in A375 cells resistant to BRAFi and MEKi (cobimetinib 1 µM and trametinib 0.1 µM).C–GRelative miRNA expression levels were quantified in human melanoma cell lines (M238P, UACC62P, 1205Lu) or short‐term patient‐derived cell lines (MM034, MM099) treated or not for 72 h with MAPK‐targeted therapies BRAFi (vemurafenib 3 µM), MEKi (trametinib 1 µM), or BRAFi + MEKi (1 µM) by RT‐qPCR and normalized to miR‐16‐5p.HRelative miRNA expression levels were quantified in M238R cells treated or not for 72 h with BIBF1120 (2 µM) or CP673451 (2 µM). (C–H) Data are represented as mean ± SE from a triplicate representative of at least three independent experiments. Paired Student's *t*‐test was used for statistical analysis. *P ≤ 0.05, **P ≤ 0.01, ***P ≤ 0.001, and ****P ≤ 0.0001.

Next, we investigated the molecular mechanisms associated with the emergence of MAPKi‐induced mesenchymal and pro‐fibrotic phenotype and its inhibition by nintedanib/BIBF1120. Because several microRNAs (miRNAs), named FibromiRs, have been shown to play key roles in the initiation and progression of fibrotic processes in various organs (Ishida & Selaru, 2013; Pottier *et al*, 2014; Hanna *et al*, 2019; Savary *et al*, 2019), we performed an expression screening to compare the level of these FibromiRs in BRAFV600E mutant melanoma cells sensitive to MAPK‐targeted therapies (M229P, M238P, M249P) compared to their corresponding resistant counterparts (Nazarian *et al*, 2010). The screening identified miR‐143‐3p and miR‐145‐5p, localized within the miR‐143/145 cluster on chromosome 5 as the best hits with a strong upregulation in AXLhigh MITFlow mesenchymal‐like resistant M238R and M229R cells tested compared to parental cells (Figs 2A and EV2A). Similar results were obtained in the mesenchymal resistant UACC62R cells (Misek *et al*, 2020) (Fig EV2A). In contrast, acquisition of resistance through secondary NRAS mutation was not associated with increased expression of miR‐143‐3p and miR‐145‐5p in the non‐mesenchymal AXLlow–MITFhigh M249R cells (Figs 2A and EV2A). Upregulation of miR‐143/145 cluster expression was also observed in BRAFi/MEKi double‐resistant (DR) melanoma clones described in Shen *et al* (2019). Interestingly, expression levels of the two miRNAs were more pronounced in acquired DR melanoma cells displaying a mesenchymal‐like cell state (Fig EV2B). We next examined whether a treatment with BRAFi, MEKi, or a combination of both was able to modulate the expression of the cluster. The two drugs, alone or in combination, significantly increased miR‐143‐3p and miR‐145‐5p expression levels in all BRAFV600E mutant melanoma cells tested including patient‐derived short‐term melanoma cultures (Fig EV2C–G). This strong induction was abolished when the BRAFi/MEKi treatment was combined with BIBF1120, both in melanoma cell lines cultured *in vitro* (Fig 2B) and in the YUMM.1.7 syngeneic model (Fig 2C) presented in Fig 1. Overall, the expression of the miR‐143/‐145 cluster paralleled the phenotypic switch associated with a mesenchymal resistant phenotype.

Given the critical role of RTK upregulation such as PDGFRβ and of the pro‐fibrotic TGF‐β signaling pathway overactivation in mesenchymal resistance (Nazarian *et al*, 2010; Sun *et al*, 2014; Diazzi *et al*, 2020; Girard *et al*, 2020), we stimulated MAPKi‐sensitive melanoma cells with PDGF‐BB or with TGF‐β and analyzed miR‐143‐3p and miR‐145‐5p expression. Both TGF‐β and PDGF‐BB triggered a strong upregulation of miR‐143/‐145 expression in M238P cells (Fig 2D). Conversely, treatment of mesenchymal BRAFi‐resistant M238R cells with BIBF1120 but also with the TGF‐β receptor inhibitor SB431542, the pan‐AKT inhibitor GSK690693 or the PDGFR inhibitor CP673451 significantly decreased the expression of the two mature miRNAs (Figs 2E and EV2H), indicating that both PDGF and TGF‐β pathways control the expression of the miR‐143/‐145 cluster in melanoma cells.

Finally, we investigated the expression of these miRNAs in several patient‐derived xenograft (PDX) samples that acquired resistance to BRAFi/MEKi combotherapy and exhibited distinct phenotypic and molecular profiles (Fig 2F) (Marin‐Bejar *et al*, 2021). Upregulation of miR‐143/‐145 cluster between therapy naïve and resistant cells was observed in two different PDX samples, MEL015 and MEL003, with a predominant invasive/undifferentiated transcriptome profile (Fig 2F and G) (Marin‐Bejar *et al*, 2021). The MEL015‐resistant model also presented elevated expression of ECM remodeling, myofibroblast, and pro‐fibrotic markers such as COL1A1, LOXL2, CYR61, THBS1, and MYL9. In contrast, we did not observe an upregulation of the cluster in drug‐resistant lesions from the two additional PDX models, MEL006 and MEL047, in which the mesenchymal‐like signature is not overrepresented (Fig 2F and G). These data indicate that upregulation of the pro‐fibrotic miR‐143/‐145 cluster is also observed in PDX MAPKi‐resistant melanomas associated with an invasive/undifferentiated transcriptome profile.

### miR‐143/‐145 cluster promotes melanoma cell dedifferentiation toward a pro‐fibrotic mesenchymal‐like state and resistance to MAPK therapeutics

**Figure 3:**
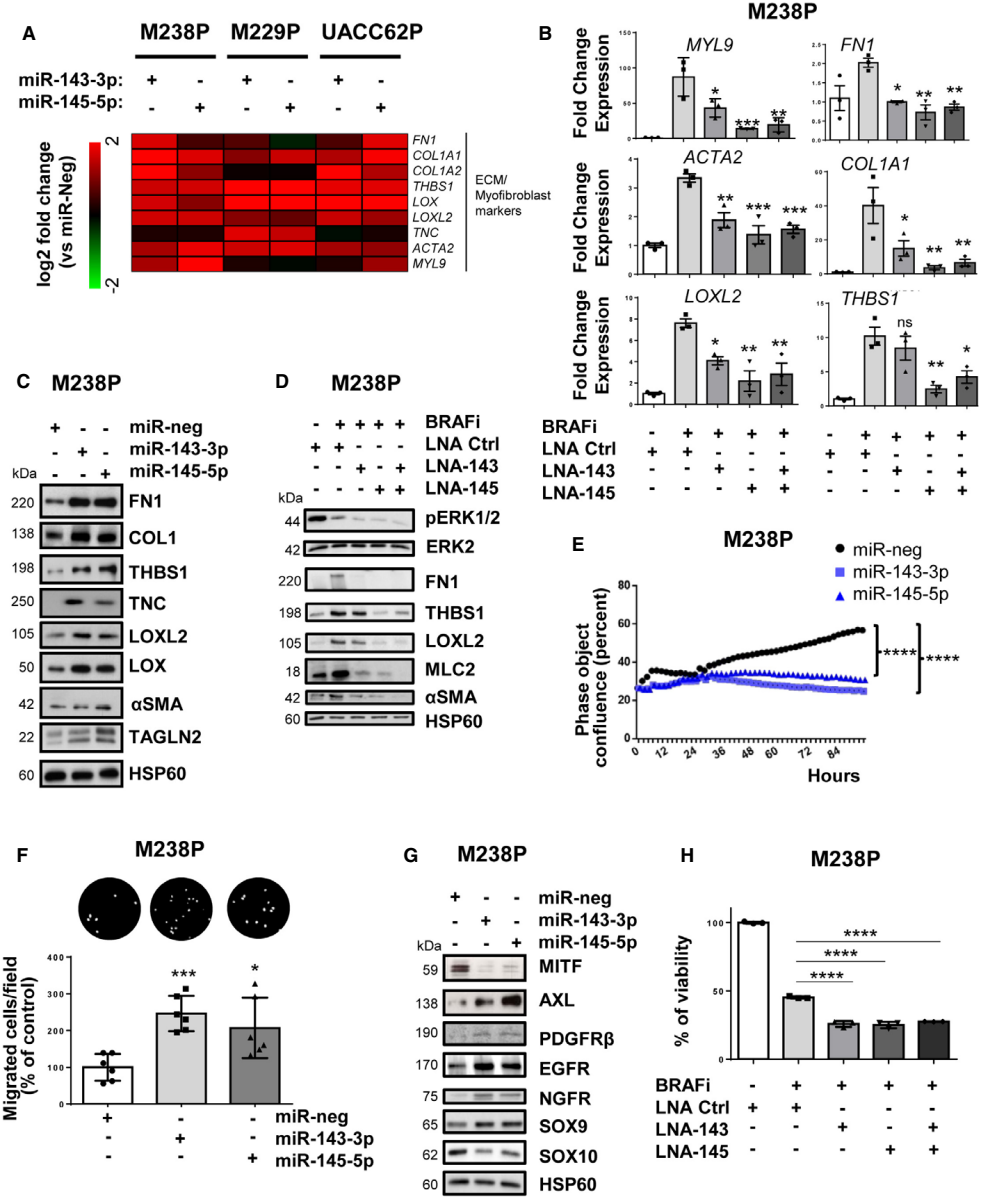
miR‐143/‐145 cluster promotes ECM reprogramming, melanoma cell dedifferentiation, and drug resistance Heatmap showing the differential expression of a selection of ECM‐related genes, cytoskeleton, and myofibroblast markers in three distinct cell lines (M238P, UACC62P, and M229P) transfected with the indicated mimics (control (miR‐neg), miR‐143 or miR‐145 mimics, 72 h, 30 nM), assessed by RT‐qPCR (*n* = 3).M238P cells were treated 72 h with BRAFi (vemurafenib, 3 μM) in the presence or the absence of LNA‐based anti‐miR‐143 (LNA‐143) or anti‐miR‐145 (LNA‐145; 50 nM) or a combination of the two. ECM marker RT‐qPCR data are represented as mean ± SD from a triplicate representative of at least three independent experiments. One‐way ANOVA was used for statistical analysis. *P ≤ 0.05, **P ≤ 0.01, and ***P ≤ 0.001.Western blot analysis of ECM remodeling markers on total cell lysates from M238P cells transfected as in (A).Western blot analysis of ECM remodeling markers on total cell lysates from cells treated with or without BRAFi and the indicated combination of inhibitors as in (B).Proliferation curves using time‐lapse analysis of cells with the IncuCyte system. Graph shows quantification of cell confluence. Two‐way ANOVA was used for statistical analysis. ****P ≤ 0.0001.Migration assay performed in Boyden chambers. Representative images showing migrating cells in the different conditions. The histogram represents the quantitative determination of mean ± SD from six independent fields representative of three independent experiments, using ImageJ software. Paired Student's *t*‐test was used for statistical analysis. *P ≤ 0.05 and ***P ≤ 0.001.Western blot analysis of phenotype switch markers on lysates from M238P cells treated as in (A).Crystal violet viability assay of M238P cells treated 72 h with the different combinations of inhibitors in the presence or not of BRAFi (vemurafenib, 3 μM). Data are represented as mean ± SD from a triplicate representative of at least three independent experiments. One‐way ANOVA was used for statistical analysis. ****P ≤ 0.0001. Source data are available online for this figure.

To confirm a potential link between the miR‐143/‐145 cluster and ECM reprogramming, we first used a gain‐of‐function approach consisting in the transient overexpression of miR‐143‐3p or miR‐145‐5p in various therapy‐naïve melanoma cells (Appendix Fig S1A). The results showed increased expression of transcripts related to ECM structure and remodeling, as well as myofibroblast/CAF markers in cells overexpressing either miRNA compared to miR‐neg control cells (Fig 3A). Conversely, we next tested whether miR‐143‐3p or miR‐145‐5p inhibition can reverse the phenotypic pro‐fibrotic response induced by oncogenic BRAF inhibition in M238P melanoma cells. BRAFi treatment was combined with locked nucleic acid (LNA)‐modified antisense oligonucleotides (ASOs) designed against miR‐143 (LNA‐143), miR‐145 (LNA‐145), or a control LNA ASO (LNA‐Ctrl). RT‐qPCR analysis showed that the BRAFi‐induced ECM‐ and myofibroblast/CAF‐related gene signature was significantly inhibited by LNA‐143 and LNA‐145 ASOs (Fig 3B). These results were confirmed at protein level by Western blot analysis of cell lysates and conditioned media of ECM proteins and cross‐linking enzymes, as well as myofibroblast/CAF markers using same gain‐ or loss‐of‐function approaches (Fig 3C–D and Appendix Fig S1B).

**Figure EV3:**
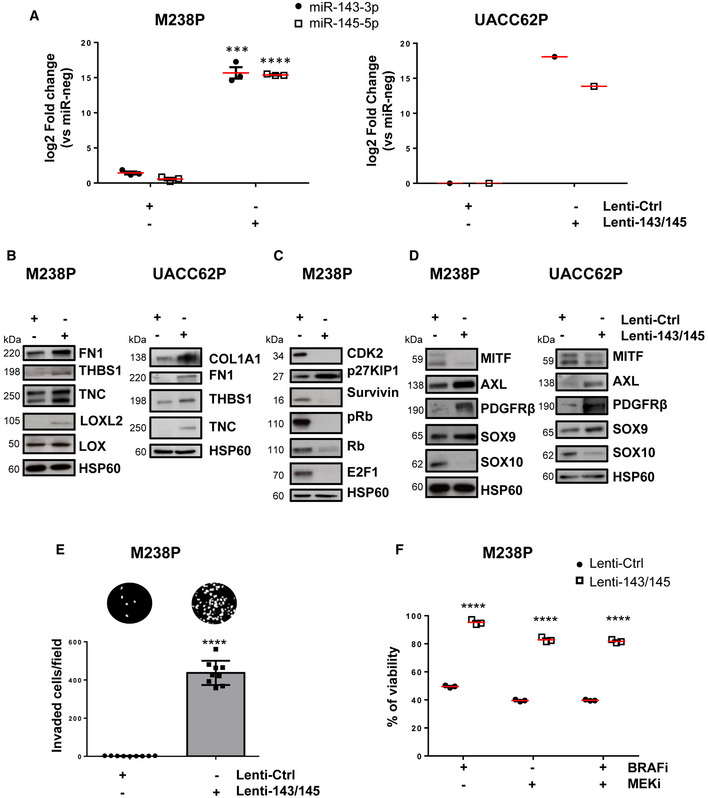
Stable expression of the miR‐143/‐145 cluster promotes ECM remodeling and drives melanoma cell dedifferentiation ART‐qPCR analysis showing the level of miR‐143‐3p and miR‐145‐5p expression after stable expression following lentivirus transduction of two melanoma cell lines (M238P, UACC62P). Data are represented as mean ± SE from a triplicate representative of at least three independent experiments. Paired Student's *t*‐test was used for statistical analysis. ***P ≤ 0.001 and ****P ≤ 0.0001.B–DWestern blot analysis of ECM remodeling (B), cell cycle (C), and phenotype switch markers (D) on total cell lysates from the different stable cell lines.EInvasion assay in Boyden chambers. Representative images show invasion in control and miR‐143/145 expressing cells (M238P). The bar graph represents the quantitative determination of data obtained using ImageJ software. Paired Student's *t*‐test was used for statistical analysis. ****P ≤ 0.0001.FViability of M238P cells transduced with a control or a miR‐143/‐145 cluster lentivirus was assessed by crystal violet staining upon MAPKi treatment (6 days, BRAFi, vemurafenib 3 µM, MEKi, trametinib, 3 µM or BRAFi + MEKi, 5 µM). Paired Student's *t*‐test was used for statistical analysis. ****P ≤ 0.0001.

We next investigated whether the cluster contributed to the acquisition of the slow cycling, undifferentiated, and invasive cell state. Melanoma cells experienced reduced cell proliferation after ectopic expression of miR‐143‐3p or miR‐145‐5p as visualized by Western blot analysis of cell cycle markers (Appendix Fig S2A) and by analysis of cell confluence by live‐cell imaging (Fig 3E and Appendix Fig S2B) with an accumulation of cells in the G0/G1 phase and a decreased percentage of cells in S phase (Appendix Fig S2C). Acquisition of the slow cycling cell state was not linked to cell death, as confirmed by annexin V/DAPI staining (Appendix Fig S2D). Inhibition of proliferation was also accompanied by enhancement of cell migratory abilities, as shown using Boyden chamber assays (Fig 3F and Appendix Fig S2E), as well as by the acquisition of a less differentiated phenotype, with decreased levels of MITF and SOX10, and increased levels of AXL, PDGFRβ, EGFR, NGFR, and SOX9 (Fig 3G and Appendix Fig S2F). Lentivirus‐mediated stable overexpression of the two miRNAs in two distinct melanoma cell lines reproduced increased ECM protein production, inhibition of cell proliferation, and transition to an undifferentiated/invasive phenotype (Fig EV3A–E) observed upon transient transfection. Acquisition of this features was also linked to a decreased intrinsic sensitivity to BRAFi/MEKi treatment, as measured by crystal violet survival assays performed on melanoma cells stably overexpressing miR‐143/‐145 cluster compared to control cells (Fig EV3F). Conversely, targeting the two miRNAs by ASOs in combination with BRAFi improved the efficacy of the targeted drug (Fig 3H and Appendix Fig S3A and B), demonstrating that miR‐143/‐145 cluster upregulation in response to BRAFV600E pathway inhibition represents a pivotal adaptive resistance mechanism to MAPK therapeutics. Of note, inhibition of miR‐145‐5p alone and combined inhibition of the two miRNAs also significantly decreased the viability of BRAFi‐resistant melanoma cells M238R (Appendix Fig S3C and D).

### Identification of miR‐143‐3p/miR‐145‐5p targets functionally associated with the undifferentiated mesenchymal‐like phenotype in melanoma cells

**Figure 4:**
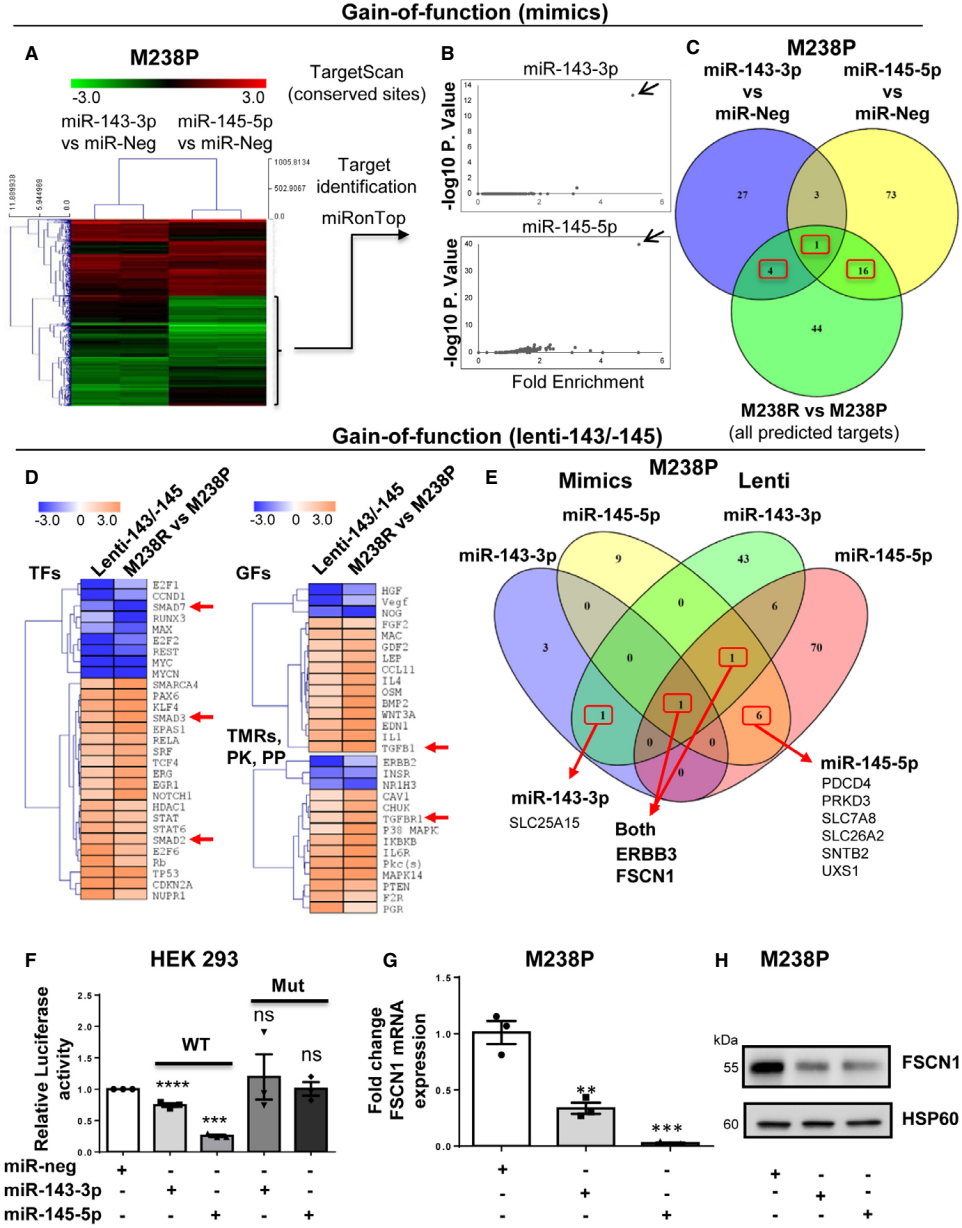
Identification of gene targets and cellular pathways functionally associated with the miR‐143/‐145 cluster‐mediated undifferentiated/mesenchymal‐like melanoma cell phenotype A–CM238P cells were transfected separately with miR‐143‐3p, miR‐145‐5p or a negative control (miR‐neg) mimics, and RNA content was analyzed using whole‐genome microarrays (dataset 1, *n* = 2). (A) Heatmap showing the genes differentially expressed after individual miRNA mimic overexpression. (B) Overrepresentation of miRNA predicted targets in the set of downregulated transcripts following miR‐143‐3p and miR‐145‐5p mimics transfection using miRonTop webtool. Each arrow indicates the corresponding overexpressed miRNA. (C) Venn diagram showing the selection of the best target candidates (red boxes) using miR‐143‐3p and miR‐145‐5p mimics transfection, as well as comparison of M238R and M238P transcriptomic profiles.D–EM238P cells were transduced with a miR‐143/‐145 construct and selected for stable expression of the cluster or transduced with a control vector, followed by RNA‐seq analysis (dataset 2, *n* = 2). (D) Heatmap highlighting the common predicted upstream regulators altered in cells stably expressing the miR‐143/‐145 cluster and M238R cells compared to control M238P cells. A subset of common regulators (out of the top 50 scores) corresponding to transcription factors (TFs), cytokines and growth factors (GFs), transmembrane receptors, kinases, and phosphatases is shown. Red arrows indicate annotations related to the TGF‐β pathway. (E) Venn diagram summarizing the comparison of the best‐predicted targets following the two gain‐of‐function approaches. Subsets of miR‐143‐3p and miR‐145‐5p predicted targets downregulated by both mimics and stable lentivirus expression are shown (red boxes).FLuciferase assay in HEK cells overexpressing miR‐143 or miR‐145 transfected with a plasmid harboring the WT or muted sequence of the miR‐143 and miR‐145 binding sites present in FSCN1 3′UTR. Each bar represents the mean ± SE of experiments performed at least in triplicate. ***P ≤ 0.001 and ****P ≤ 0.0001. *P*‐values were calculated using paired Student's *t*‐test.GRT‐qPCR analysis of FSCN1 expression in M238P cells transfected with the indicated mimics. Data are represented as mean ± SE from a triplicate representative of at least three independent experiments. Paired Student's *t*‐test was used for statistical analysis. **P ≤ 0.01 and ***P ≤ 0.001.HWestern blot analysis of FSCN1 expression in M238P cells transfected with the indicated mimics. Source data are available online for this figure.

To identify miR‐143‐3p and miR‐145‐5p targets associated with the resistant mesenchymal phenotype, we first combined *in silico* target prediction tools and experimental transcriptomic approaches using the miRonTop web tool (Le Brigand *et al*, 2010) in M238R or M238P cells following transient transfection of mimics (Fig 4A and B) or stable lentivirus transduction. Functional annotation of the gene expression profiles associated with miRNA overexpression showed a strong overlap in pathways associated with cell migration and invasion, cell cycle, and cytoskeleton organization (Appendix Table S1). The predicted targets for each of the mature miRNAs were significantly overrepresented among the downregulated genes in response to the corresponding mimics transfection (Fig 4B). A first set of target candidates were identified by crossing these predicted targets and the genes shown experimentally to be downregulated in resistant M238R cells compared to parental M238P cells (Fig 4C).

Second, RNAs from cells stably overexpressing the miR‐143/‐145 cluster were analyzed by RNA‐sequencing and processed through Ingenuity Pathway Analysis (IPA) to identify the common regulators (transcription factors, growth factors, cytokines, transmembrane receptors, kinases, and phosphatases) between parental cells overexpressing the cluster and resistant cells (Fig 4D). These analyses notably highlighted changes related to decreased cell proliferation, increased cell invasion, and fibrotic pathway activation. To narrow the best target candidates, we finally compared the best‐predicted targets based on the two different gain‐of‐function approaches (Appendix Tables S2 and S3). This strategy resulted in selecting one target candidate for miR‐143‐3p, 6 target candidates for miR‐145‐5p, and 2 target candidates for both miR‐143‐3p and miR‐145‐5p (Fig 4E). We started with investigations on the F‐acting bundling protein Fascin1 (FSCN1), a key regulator of cytoskeleton dynamics, previously associated with tumor growth, migration, invasion, and metastasis (Ma & Machesky, 2015). Using long‐read Nanopore sequencing data, we confirmed lower levels of FSCN1 transcript in M238R compared with M238P cells, while reads corresponding to the putative miR‐143/‐145 cluster primary transcript could be only detected in M238R cells (Appendix Fig S4A). The characterization of hFSCN1 3′UTR sequence revealed the presence of 2 miR‐143‐3p and 4 miR‐145‐5p binding sites. Validation of these sites was first performed using a luciferase reporter corresponding to the full 3′UTR FSCN1 harboring WT or a mutated sequence of the miRNA recognition elements (Fig 4F and Appendix Fig S4B). Finally, qPCR and Western blot analyses confirmed that FSCN1 was downregulated at both mRNA and protein levels upon miR‐143‐3p and miR‐145‐5p ectopic expression in various melanoma cells and in cells stably overexpressing the cluster (Fig 4G and H and Appendix Fig S4C and D).

### FSCN1 is a functional miR‐143/‐145 target contributing to the phenotypic switch toward the undifferentiated/mesenchymal‐like and resistant state

**Figure EV4:**
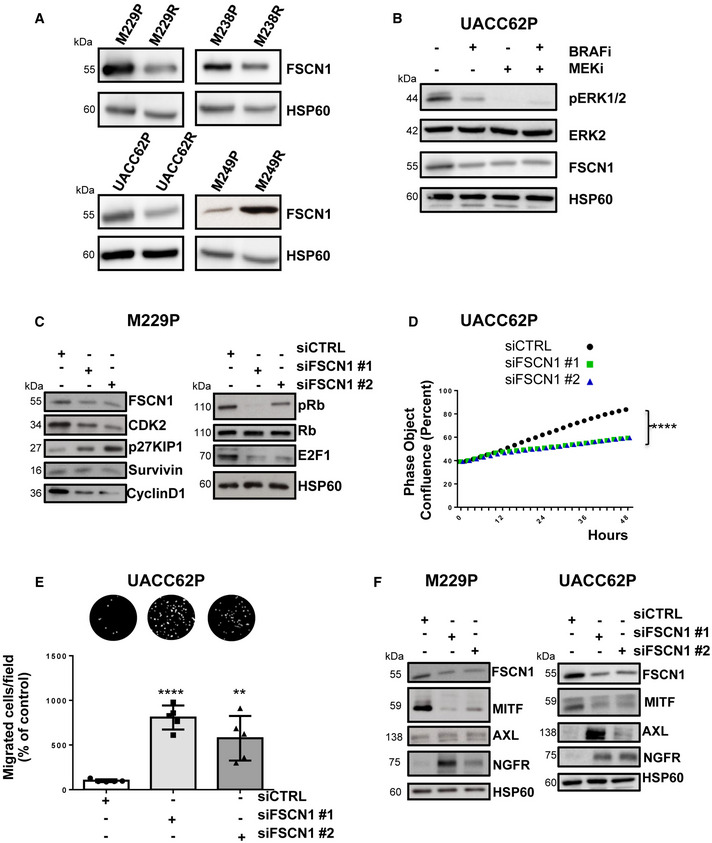
FSCN1 is a functional miR‐143/‐145 target contributing to the phenotypic switch toward an invasive dedifferentiated state AWestern blot analysis of FSCN1 expression in parental and paired resistant cells (M238, UACC62, M229, M249).BWestern blot analysis of FSCN1 levels in parental cells (UACC62P) treated with BRAFi, (vemurafenib, 3 µM), MEKi (trametinib, 1 µM), or BRAFi + MEKi (1 µM) for 72 h.C–FCells were transfected with two different sequences of siRNAs vs FSCN1 or with a control siRNA (100 nM). (C) Western blot analysis of cell cycle markers on cell lysates from M229P cells cultured for 72 h following transfection with the different siRNAs. (D) Proliferation curves using time‐lapse analysis of cells with the IncuCyte system. Graph shows quantification of cell confluence. Two‐way ANOVA was used for statistical analysis. ****P ≤ 0.0001. (E) Migration assay performed in Boyden chambers. Representative images showing migration of UACC62P cells in the indicated conditions. The bar graph represents the quantitative determination of data obtained using ImageJ software. Paired Student's *t*‐test was used for statistical analysis. **P ≤ 0.01 and ****P ≤ 0.0001. (F) Western blot analysis of phenotype switch markers on cell lysates from cells (M229P and UACC62P) transfected with the different siRNAs.

**Figure 5:**
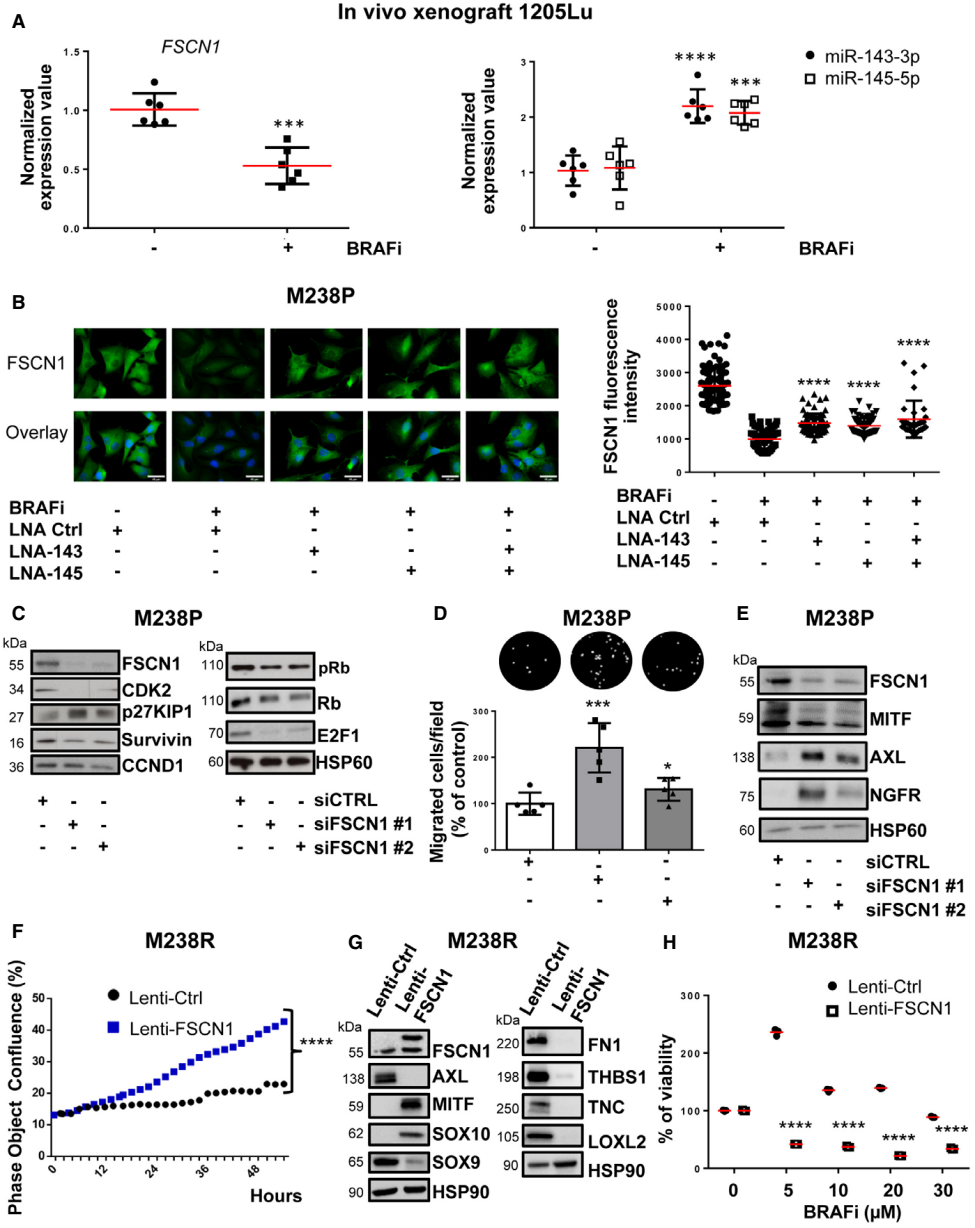
FSCN1 is a functional miR‐143/‐145 target contributing to the phenotypic switch toward the undifferentiated/mesenchymal‐like state AqPCR analysis of FSCN1, miR‐143, and miR‐145 expression in a 1205Lu xenograft nude mouse model treated with the BRAFi vemurafenib compared to control mice (*n* = 6). Paired Student's *t*‐test was used for statistical analysis. ***P ≤ 0.001 and ****P ≤ 0.0001.BFSCN1 immunofluorescent staining and quantification of fluorescence intensity in M238P cells treated or not with BRAFi (vemurafenib, 3 μM) in the presence or the absence of LNA‐based anti‐miR‐143 (LNA‐143) or anti‐miR‐145 (LNA‐145; 50 nM) or a combination of the two. Data are represented as scatter plots with mean ± SD from 10 independent fields representative of three independent experiments; the Mann–Whitney *U*‐test was used for statistical analysis. ****P ≤ 0.0001. Scale bar 40 μm.C–EM238P cells were transfected with two different sequences of siRNAs vs FSCN1 or with a control siRNA (72 h, 100 nM). (C) Western blot analysis of cell cycle markers on cell lysates from M238P cells cultured for 72 h following transfection with the indicated siRNAs. (D) Migration assay performed in Boyden chambers. Representative images showing migration of M238P cells treated with the indicated siRNAs. The bar graph represents the quantitative determination of data obtained of mean ± SD from 5 independent fields representative of three independent experiments, using ImageJ software. Paired Student's *t*‐test was used for statistical analysis. *P ≤ 0.05 and ***P ≤ 0.001. (E) Western blot analysis of phenotype switch markers on cell lysates from M238P cells transfected with the indicated siRNAs.F–HBRAFi‐resistant M238R cells overexpressing FSCN1 were obtained after transduction with a FSCN1 lentiviral construct. M238R transduced with a Ctrl lentivirus were used as control. (F) Effect of FSCN1 overexpression on cell proliferation assessed by time‐lapse analysis using the IncuCyte system. Graph shows quantification of cell confluence. Two‐way ANOVA was used for statistical analysis. ****P ≤ 0.0001. (G) Western blot analysis of FSCN1, phenotype switch markers, and ECM remodeling markers on cell lysates from control and FSCN1 overexpressing cells. (H) Crystal violet viability assay of M238R cells stably overexpressing FSCN1 during 6 days with the indicated doses of the BRAFi vemurafenib. Data are represented as mean ± SD from a triplicate representative of at least three independent experiments. Paired Student's *t*‐test was used for statistical analysis. ****P ≤ 0.0001. Source data are available online for this figure.

Considering the strong expression of the miR‐143/‐145 in BRAFV600E mutant mesenchymal‐like resistant cells compared to their parental counterparts, we compared FSCN1 expression levels in various pairs of resistant and sensitive melanoma cell lines. Western blot indicated that FSCN1 protein levels were lower in undifferentiated mesenchymal resistant cells compared to parental cells, while on the contrary, they were elevated in M249R melanoma cells acquiring genetic resistance compared to parental cells (Fig EV4A). We then confirmed the opposite regulation of FSCN1 and miR‐143/‐145 cluster expression upon BRAFi treatment both *in vivo* using xenografted nude mice (tumors analyzed at the endpoint, as described in Girard *et al* (2020)) and *in vitro* with different human BRAF mutant melanoma cells (Figs 5A and EV4B). Finally, FSCN1 levels were partially restored in M238P cells treated with BRAFi when vemurafenib was combined with the LNA‐miR‐143, LNA‐miR‐145, or a combination of the two, as visualized by immunofluorescence staining (Fig 5B), suggesting that FSCN1 downregulation upon BRAFi exposure is due to increased expression of miR‐143‐3p and miR‐145‐5p.

To evaluate the influence of FSCN1 downregulation among the various cellular effects mediated by miR‐143‐3p and miR‐145‐5p, we then performed a loss‐of‐function experiment using FSCN1‐specific siRNAs in BRAF‐mutant parental melanoma cells. Western blot analysis of cell cycle markers (Figs 5C and EV4C) and cell confluence analysis by live‐cell imaging (Fig EV4D) showed reduced proliferation after downregulation of FSCN1. This slow‐cycling state induced by FSCN1 silencing was accompanied by an enhancement in cell migratory abilities (Figs 5D and EV4E). Moreover, FSCN1 invalidation modulated melanoma cell differentiation state, inducing the switch to a poorly differentiated phenotype characterized by reduced levels of MITF and increased levels of AXL and NGFR (Figs 5E and EV4F).

Using the opposite strategy, we then asked whether ectopic expression of FSCN1 was able to revert the mesenchymal‐like phenotype and restore drug sensitivity in BRAFi‐resistant melanoma cells. Resistant cells transduced for stable FSCN1 overexpression displayed an increased proliferative rate compared to cells transduced with a control lentivirus (Fig 5F). This effect was linked to diminished migratory abilities (Appendix Fig S5A). This phenotypic transition was further confirmed by Western blot analysis of differentiation markers in various mesenchymal resistant cells, with increased expression of melanocytic markers (MITF, SOX10) and decreased levels of invasive markers (AXL, SOX9), as well as decreased production of ECM proteins and the ECM remodeling enzyme LOXL2 (Fig 5G and Appendix Fig S5B). Finally, mirroring the effect of miR‐143/‐145 ASOs, forced expression of FSCN1 in M238R cells decreased viability in the presence of BRAFi (Fig 5H). Altogether, these data underline the central function of the miR‐143/‐145/FSCN1 axis in the acquisition of a dedifferentiated, mesenchymal‐like cell state associated with therapy resistance.

### The miR‐143‐/145 cluster/FSCN1 axis regulates actin cytoskeleton dynamics and mechanopathways

**Figure 6:**
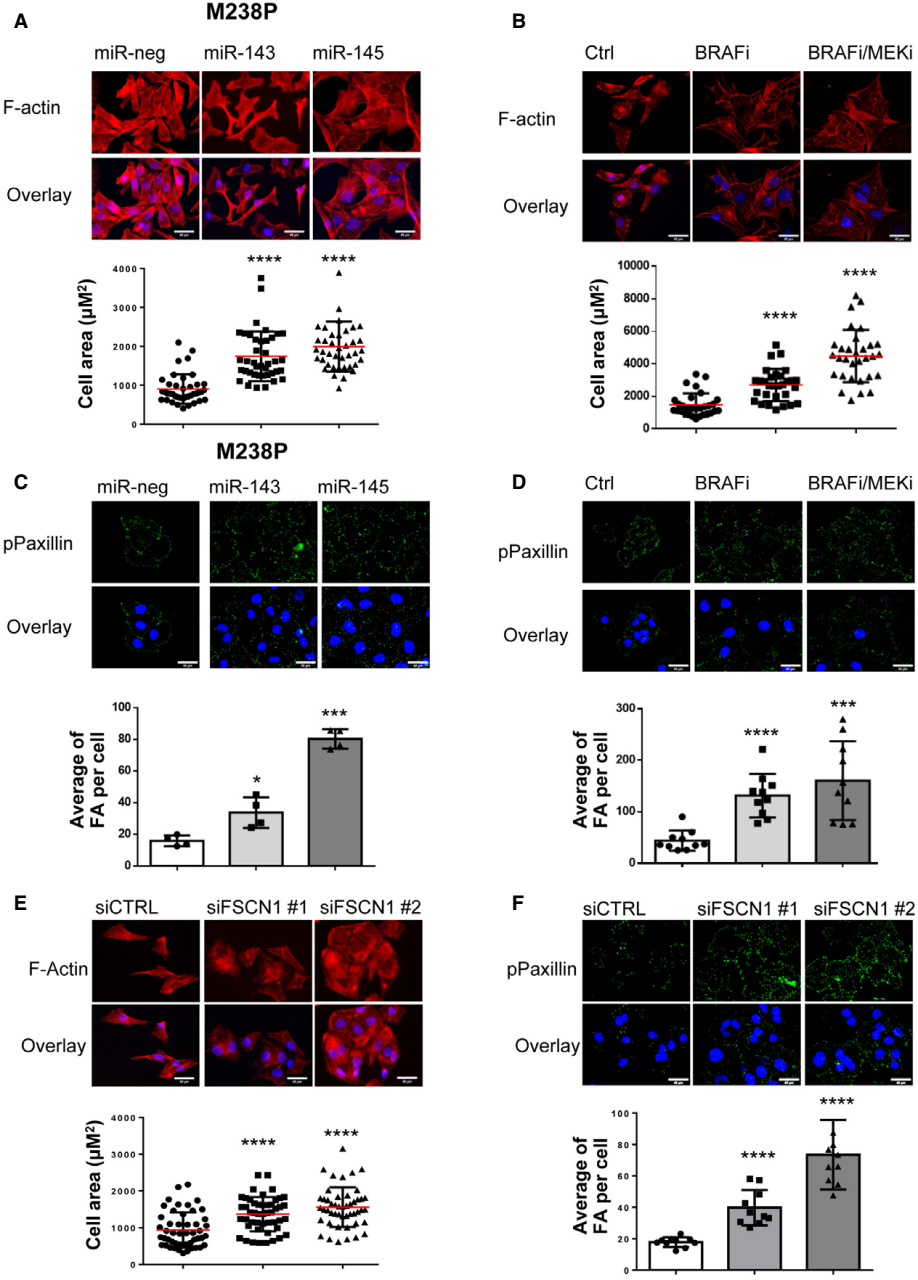
Regulation of actin cytoskeleton dynamics and focal adhesions by miR‐143/‐145 cluster /FSCN1 axis A–DM238P cells were transfected with miR‐143‐3p, miR‐145‐5p, or a control mimic (miR‐neg; 72 h, 30 nM).(B, D) M238P cells were treated 72 h with BRAFi (vemurafenib, 3 µM) or a combination of BRAFi (vemurafenib, 0.5 µM) and MEKi (trametinib, 1 µM). (A‐B) Images and quantification of cell area in cells stained for F‐actin (red) and nuclei (blue). Data are represented as scatter plot with mean ± SD (*n* ≥ 30 cells per condition). The Mann–Whitney *U*‐test was used for statistical analysis. ****P ≤ 0.0001. Scale bar 40 μm. (C, D) Images and quantification of focal adhesions number in cells stained for paxillin (green) and nuclei (blue). Focal adhesions (FA) number is represented as mean ± SD (*n* ≥ 30 cells per condition). Each point represents the average number of focal adhesions per cell calculated for each field. Paired Student's *t*‐test has been used for statistical analysis. *P ≤ 0.01, ***P ≤ 0.001, and ****P ≤ 0.0001. Scale bar 40 μm.E, FM238P cells were transfected with two different sequences of siRNAs vs FSCN1 or with a control siRNA (72 h, 100 nM). (E) Images and quantification of cell area in cells stained for F‐actin (red) and nuclei (blue). Data are represented as scatter plot with mean ± SD (*n* ≥ 30 cells per condition). The Mann–Whitney *U*‐test was used for statistical analysis. ****P ≤ 0.0001. Scale bar 40 μm. (F) Images and quantification of focal adhesions (FA) number in cells stained for pPaxillin (green) and nuclei (blue). Focal adhesions number is represented as mean ± SD (*n* ≥ 30 cells per condition). Each point represents the average number of focal adhesions per cell calculated for each field. Paired Student's *t*‐test was used for statistical analysis. ****P ≤ 0.0001. Scale bar 40 μm. Source data are available online for this figure.

Acquisition of the mesenchymal‐like resistant state implies a massive cytoskeletal rearrangement reflected by morphological changes with cells assuming a flattened and spindle‐like shape (Nazarian *et al*, 2010; Girard *et al*, 2020; Misek *et al*, 2020). Based on the key function of FSCN1 in F‐actin microfilament reorganization (Hashimoto *et al*, 2011; Jansen *et al*, 2011; Elkhatib *et al*, 2014), we specifically analyzed the contribution of the miR‐143/‐145 cluster/FSCN1 axis on actin cytoskeleton dynamics. Transient overexpression of miR‐143‐3p and miR‐145‐5p triggered morphological changes similar to the ones observed upon BRAFi alone or BRAFi plus MEKi administration, as shown by F‐actin staining and increased cell area (Fig 6A and B and Appendix Fig S6A and B). To better understand the crosstalk between ECM remodeling and rearranged actin dynamics, we performed immunofluorescent staining of focal adhesions, multi‐protein structures that connect ECM to the acto‐myosin cytoskeleton. An increased number of focal adhesions revealed by phospho‐paxillin staining characterized melanoma cells expressing miR‐143‐3p or miR‐145‐5p or melanoma cells upon BRAF pathway inhibition (Fig 6C and D and Appendix Fig S6C and D). Changes in focal adhesion dynamics following miR‐143 or miR‐145 overexpression were also confirmed by Western blot analysis of focal adhesion components such as phospho‐FAK and phospho‐SRC (Appendix Fig S6E). In addition, we observed an increase in phosphorylated and total forms of MLC2 and phosphorylated Signal Transducer and Activator of Transcription 3 (STAT3) upon cluster overexpression, suggesting the activation of the ROCK/JAK/STAT3 acto‐myosin contractility pathway by the two miRNAs. We then investigated whether FSCN1 downregulation produced a similar effect on actin dynamics. Indeed, FSCN1 knockdown led to actin cytoskeleton reorganization with a significant cell area increase (Fig 6E and Appendix Fig S6F), as well as an increased number of focal adhesions per cell (Fig 6F and Appendix Fig S6G).

**Figure 7:**
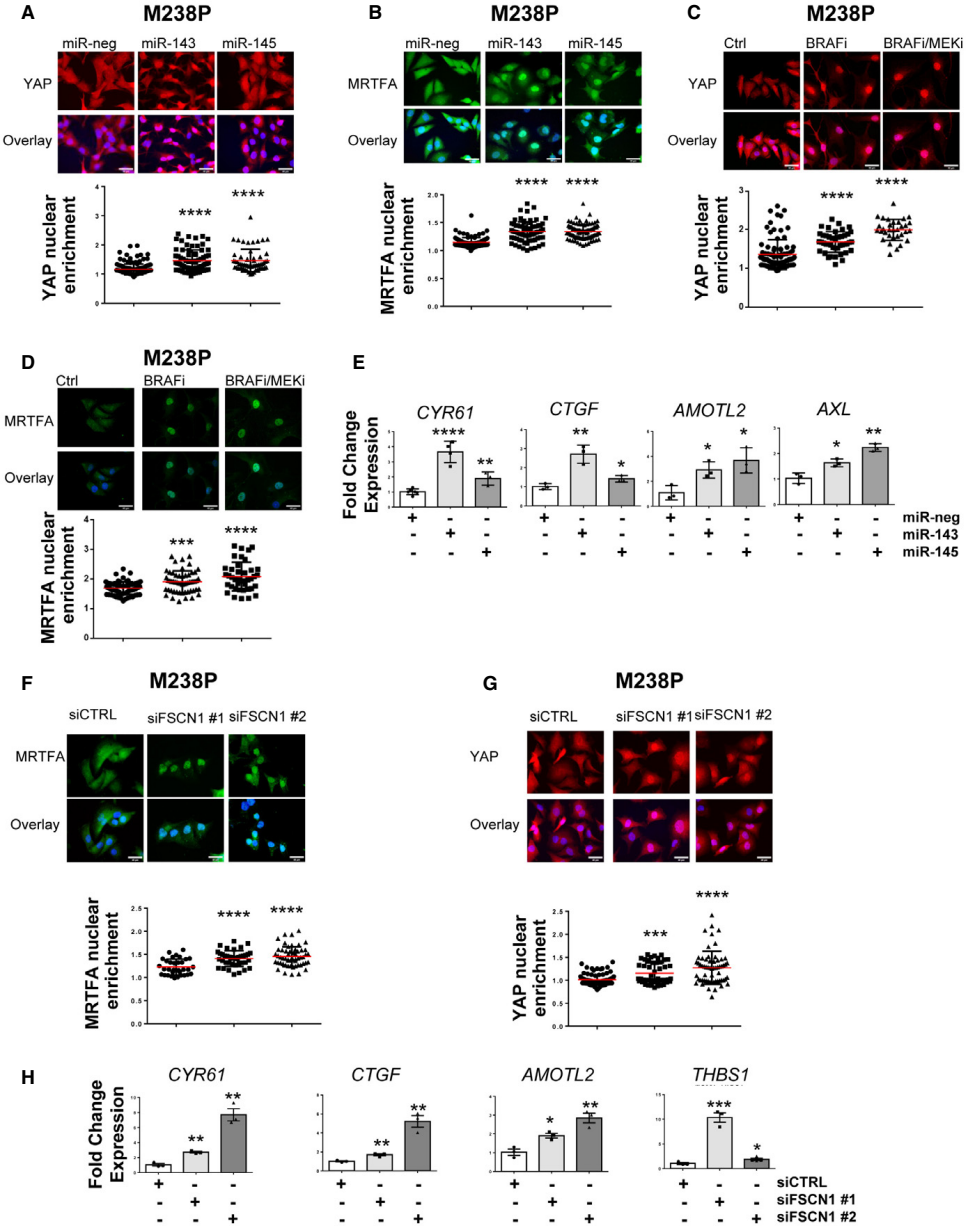
Regulation of mechanosensitive transcriptional coactivators YAP and MRTF by the miR‐143/‐145 cluster / FSCN1 axis A, BM238P cells were transfected with miR‐143‐3p, miR‐145‐5p, or a control mimic (miR‐neg; 72 h, 30 nM). Effect of miR‐143‐3p or miR‐145‐5p overexpression on YAP (A) and MRTFA (B) nuclear translocation by immunofluorescence. Data are represented as scatter plots with mean ± SD (*n* ≥ 30 cells per condition). Each point represents the nuclear/cytoplasm ratio. The Mann‐Whitney *U*‐test was used for statistical analysis. ****P ≤ 0.0001. Scale bar 40 μm.C, DM238P cells were treated 72 h with BRAFi (vemurafenib, 3 µM) or with a combination of BRAFi (vemurafenib, 0.5 µM) and MEKi (trametinib, 1 µM). Effect of BRAFi or BRAFi plus MEKi on YAP (C) and MRTFA (D) nuclear translocation by immunofluorescence. Data are represented as scatter plot with mean ± SD (*n* ≥ 30 cells per condition). Each point represents the nuclear/cytoplasm ratio. The Mann–Whitney *U*‐test was used for statistical analysis. ***P ≤ 0.001 and ****P ≤ 0.0001. Scale bar 40 μm.EEffect of miR‐143‐3p or miR‐145‐5p overexpression on the expression of YAP/MRTF target genes assessed by RT‐qPCR. Data are normalized to the expression in control cells. Data are represented as mean ± SD from a triplicate representative of at least 3 independent experiments. Paired Student's *t*‐test was used for statistical analysis. *P ≤ 0.05, **P ≤ 0.01, and ****P ≤ 0.0001.F–HM238P cells were transfected with two different sequences of siRNAs vs FSCN1 or with a control siRNA (72 h, 100 nM). Effect of FSCN1 downregulation on MRTFA (F) and YAP1 (G) nuclear translocation assessed by immunofluorescence in M238P. Data are represented as scatter plot with mean ± SD (*n* ≥ 30 cells per condition). Each point represents the nuclear/cytoplasm ratio. The Mann–Whitney *U*‐test was used for statistical analysis. ***P ≤ 0.001 and ****P ≤ 0.0001. Scale bar 40 μm. (H) RT‐qPCR analysis for the expression of MRTFA/YAP target genes in M238P cells transfected with the indicated siRNAs. Data are normalized to the expression in parental cells. Data are represented as mean ± SE from a triplicate representative of at least 3 independent experiments. Paired Student's *t*‐test was used for statistical analysis. *P ≤ 0.05, **P ≤ 0.01, and ***P ≤ 0.001. Scale bar 40 μm. Source data are available online for this figure.

**Figure EV5:**
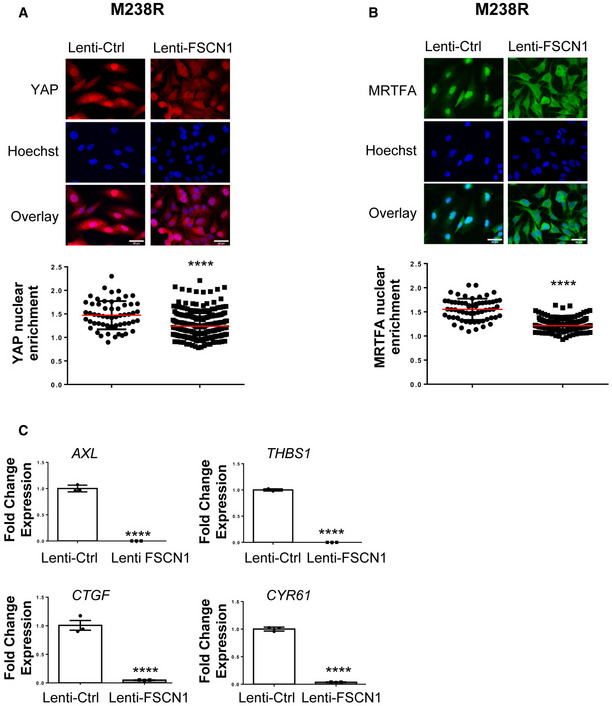
FSCN1 restoration impairs the activation of mechanopathways BRAFi‐resistant M238R cells overexpressing FSCN1 were obtained after transduction with a FSCN1 lentiviral construct. M238R cells transduced with a Ctrl lentivirus were used as control.
A, BEffect of FSCN1 overexpression on YAP (A) and MRTFA (B) nuclear translocation assessed by immunofluorescence in cells stained for YAP (red) or MRTFA (green) and nuclei (blue). Data are represented as scatter plot with mean ± SD (*n* ≥ 30 cells per condition). Each point represents the nuclear/cytoplasm ratio. The Mann–Whitney *U*‐test was used for statistical analysis. ****P ≤ 0.0001. Scale bar 40 μm.CRT‐qPCR analysis for the expression of YAP1/MRTFA target genes in M238R cells stably overexpressing FSCN1. Data are normalized to the expression in parental control cells. Data are represented as mean ± SE from a triplicate representative of at least 3 independent experiments. Paired Student's *t*‐test was used for statistical analysis. ****P ≤ 0.0001.

Acto‐myosin remodeling critically regulates the cellular localization of mechanotransducers such as the Hippo pathway transcriptional co‐activator YAP and the serum responsive factor co‐activator MRTFA, two factors previously associated with resistance to MAPK‐targeting therapies and pro‐fibrotic responses (Kim *et al*, 2016; Diazzi *et al*, 2020; Girard *et al*, 2020; Misek *et al*, 2020). Expression of miR‐143‐3p and miR‐145‐5p in therapy‐naïve melanoma cells enhanced YAP and MRTFA nuclear localization as shown by immunofluorescent staining (Fig 7A and B and Appendix Fig S7A and B). As previously described (Kim *et al*, 2016; Girard *et al*, 2020; Misek *et al*, 2020), similar observations were made upon administration of MAPKi to melanoma cells (Fig 7C and D and Appendix Fig S7C and D). Increased YAP and MRTF activity upon miR‐143/145 overexpression was also confirmed by upregulated expression of several target genes (CTGF, CYR61, AMOTL2, THBS1, AXL), as shown by RT‐qPCR analysis (Fig 7E and Appendix Fig S7E). Again, these changes in cytoskeleton organization were reproduced by FSCN1 knockdown, with nuclear translocation of MRTFA and YAP (Fig 7F and G and Appendix Fig S7F) and increased target gene expression (Fig 7H). Finally, using the opposite strategy, we tested whether ectopic expression of FSCN1 was able to revert the constitutive activation of mechanical pathways typical of this cell state. Indeed, forced expression of FSCN1 in mesenchymal resistant cells significantly attenuated nuclear localization of YAP and MRTFA, as well as their transcriptional activity (Fig EV5A–C). Overall, our data highlight the central function of the miR‐143/‐145/FSCN1 axis in the regulation of actin cytoskeleton dynamics and mechanopathways, leading to the acquisition of an undifferentiated and drug‐resistant mesenchymal‐like cell state.

## Discussion

**Figure 8:**
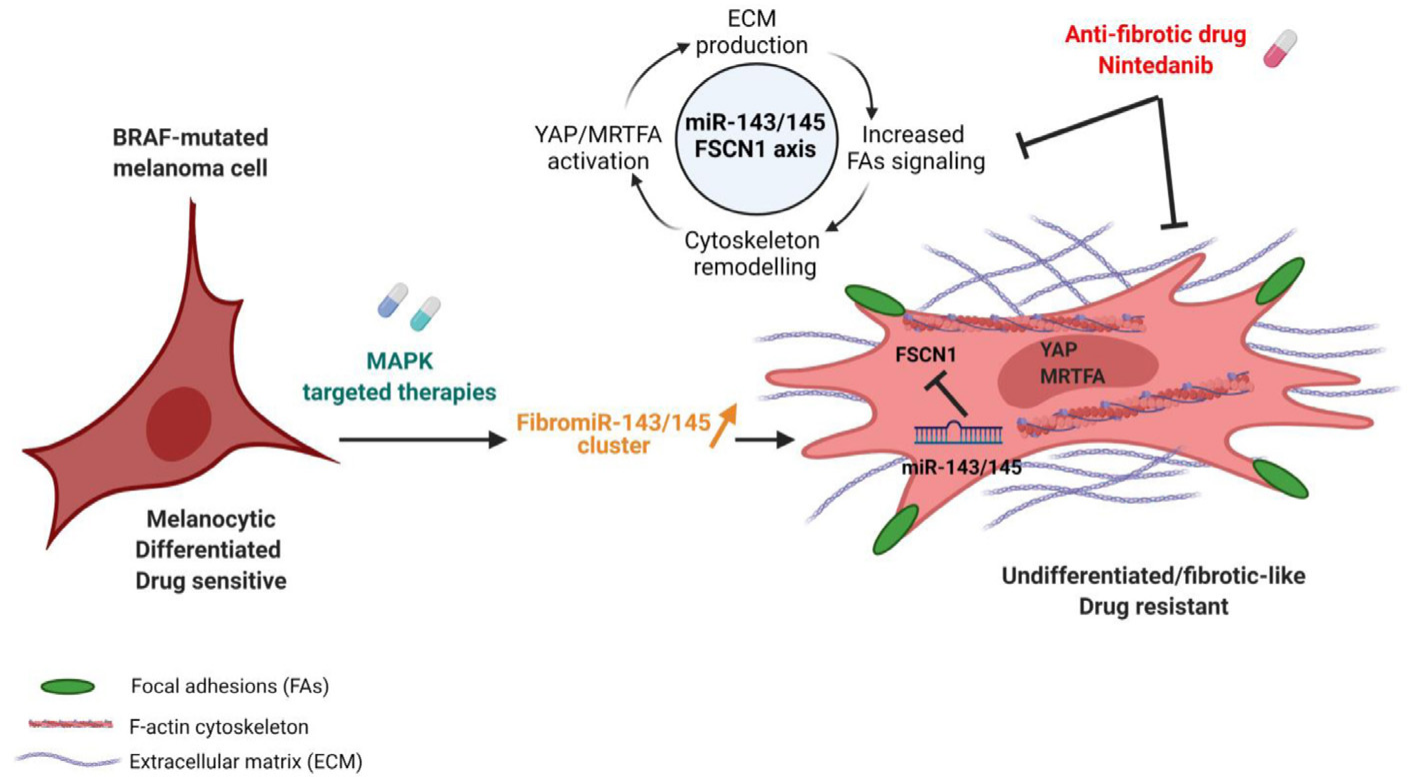
Proposed model for a role of the pro‐fibrotic miR‐143/‐145 cluster in phenotypic plasticity‐driven resistance induced by MAPK‐targeted therapies and its potential targeting by nintedanib BRAFi/MAPKi therapy of BRAF‐mutated drug‐sensitive melanoma cells induces upregulation of miR‐143/‐145 cluster expression levels. The two mature miRNAs generated from this cluster, miR‐143‐3p and miR‐145‐5p, collaborate to mediate phenotypic transition toward a drug‐resistant undifferentiated mesenchymal‐like state by targeting Fascin actin‐bundling protein 1 (FSCN1), increasing focal adhesion signaling (FAs), cytoskeleton remodeling, YAP/MRTFA mechanotransduction pathways activation and ECM production. This mechanism leading to an undifferentiated fibrotic‐like drug‐resistant phenotype can be targeted by the anti‐fibrotic drug nintedanib. Created with BioRender.com.

Treatments against advanced melanoma invariably end with therapy resistance and failure. Preventing resistance on therapies targeting the MAPK oncogenic pathway still remains a challenge in successful melanoma clinical management. Our present study reveals that combination of the anti‐fibrotic drug nintedanib with targeted therapy provides therapeutic benefit in preclinical models of melanoma. We showed that nintedanib is able to prevent the acquisition by melanoma cells of an undifferentiated mesenchymal‐like phenotype, an aggressive cell state previously shown to be associated with the expression of pro‐fibrotic markers, acquisition of myofibroblast/CAF‐like activities, and enhanced mechanosignaling and drug resistance (Diazzi *et al*, 2020; Girard *et al*, 2020). Importantly, we provided evidence that the triplet combination BRAFi, MEKi, and nintedanib is active to normalize the fibrous collagen network, delay the onset of resistance, and improve mice survival. We also confirmed the efficacy of this therapeutic combination in human BRAFV600E mutant melanoma cells and described its potential to impair phenotype switching and improve response to targeted therapy (Fig 8).

Nintedanib (BIBF1120) is a multiple tyrosine kinase inhibitor, targeting PDGFR (α and β), FGFR‐1, FGFR‐2, FGFR‐3, and FGFR‐4 and VEGFR‐1, VEGFR‐2, and VEGFR‐3, as well as several intracellular tyrosine kinases such as Src, Lck, or Lyn. It has been approved for the treatment of IPF following several clinical trials demonstrating clinical efficacy in slowing disease progression (Bonella *et al*, 2015). Nintedanib was shown to interfere with fundamental processes in lung fibrosis in a variety of *in vitro* assays performed on primary lung fibroblasts from patients with IPF, notably the inhibition of growth factor‐induced proliferation/migration and TGF‐β‐induced myofibroblast activation, as well as the downregulation of ECM proteins (Wollin *et al*, 2015). However, although substantial preclinical evidence demonstrates that nintedanib has anti‐fibrotic but also anti‐inflammatory and anti‐angiogenic activity, the exact contribution of inhibition of specific kinases to the activity of the drug in IPF has not been established and its precise anti‐fibrotic mechanism(s) of action is not known. In melanoma, the effects of nintedanib are likely achieved through the normalization of the fibrotic and drug‐protective ECM generated by melanoma cells upon MAPK‐targeted therapy exposure. Interestingly, our *in vitro* observations suggest that nintedanib's effect on melanoma cells cannot be solely explain through PDGFRβ inhibition. In addition, a direct effect of nintedanib on CAF *in vivo* is also possible regarding the described paradoxical activation of CAF upon BRAF inhibition in melanoma (Fedorenko *et al*, 2015; Hirata *et al*, 2015). In line with this hypothesis, nintedanib was shown to have inhibitory effects on CAF proliferation and activation in a murine model of melanoma (Kato *et al*, 2021).

Herein, we found that combined administration of nintedanib and MAPK‐targeted therapy dampens the increased miR‐143/‐145 cluster expression triggered by oncogenic BRAF pathway inhibition, suggesting that inhibition of ECM reprogramming in the presence of nintedanib is, at least partially, mediated by preventing upregulation of these two FibromiRs. Induction of the miR‐143/‐145 cluster paralleled the phenotypic switch associated with the dedifferentiated phenotype, and high expression levels of the two miRNAs are correlated with the mesenchymal MAPKi‐resistant phenotype in all BRAFV600E mutant human melanoma cell lines known to overexpress RTKs including the PDGFRβ. Analysis of PDX models confirmed that expression levels of miR‐143‐3p and miR‐145‐5p are associated with a predominant invasive/undifferentiated transcriptomic profile in resistant lesions. Clinically, our findings are supported *in silico* by the observation that the pri‐miR‐143/‐145 precursor, MIR143HG, was part of the specific mesenchymal signature of a subset of MAPKi‐resistant cells described in Song *et al* (2017). Elevated levels of these miRNAs following BRAFi/MEKi treatment are likely due primarily to the direct inhibition of the MAPK pathway, as oncogenic signals including activation of the MAPK pathway strongly inhibit expression of the cluster in several epithelial cancers (Kent *et al*, 2013). In addition, we have shown a positive regulation of the cluster by PDGF or TGF‐β signaling, as previously observed in the context of fibrosis and smooth muscle cell differentiation (Long & Miano, 2011; Yang *et al*, 2013). This observation supports the notion that pro‐fibrotic signaling pathways typical of the mesenchymal resistance drive expression of the miR‐143/‐145 cluster in melanoma cells. Besides, the AKT pathway could also upregulate expression levels of the two miRNAs. Accordingly, previous studies stated that *PTEN* deletion favors the onset of a fibrotic phenotype in lung fibrosis and increased Fibronectin deposition in melanoma (Kuwano, 2006; Fedorenko *et al*, 2016). The observation that nintedanib abrogated both AKT activation and miR‐143/‐145 expression in melanoma cells is in agreement with the importance of this pathway for acquisition and maintenance of drug resistance. Overall, our data indicate that nintedanib can target both pro‐fibrotic and survival pathways, mediated at least in part through PDGFRβ/AKT activation and converging to miR‐143/‐145 cluster expression.

The role of miR‐143 and miR‐145 in cancer has been widely debated in the last decade (Poli *et al*, 2020). The tumor suppressive role traditionally attributed to the cluster (Das & Pillai, 2015) has been challenged by recent genetic and cellular expression studies pointing mesenchymal cells as the main source of the cluster (Kent *et al*, 2014; McCall *et al*, 2017). In melanoma, we disclosed that miR‐143‐3p and miR‐145‐5p promote the acquisition of an invasive and mesenchymal‐like phenotype linked to drug adaptation and resistance. The importance of miR‐143/145 cluster in the acquisition of this dedifferentiated state is further highlighted with a loss‐of‐function approach showing that miR‐143‐3p and miR‐145‐5p inhibitors are able to limit ECM reprogramming and activation of mechanopathways, and improve anti‐BRAF treatment efficacy. While further work using a combination of ASOs directed against the two mature miRNAs or the primary transcript is necessary to confirm these promising results in a melanoma xenograft model, we propose that the miR‐143/‐145 cluster may represent a novel attractive therapeutic target to prevent cells from switching to a mesenchymal/invasive state and tumor relapse after targeted therapy.

Our study shows that mechanistically the miR‐143/‐145 cluster functions in melanoma cells through targeting the cytoskeletal regulator FSCN1, one of the best hits identified by our screening, confirming previous studies indicating that FSCN1 is a direct target of both mature miRNAs (Kano *et al*, 2010; Liu *et al*, 2012). FSCN1 has been widely studied in several malignancies for its role in promoting invasion and metastasis. However, a complete characterization of FSCN1 functions in melanoma is still missing and some published studies are controversial (Scott *et al*, 2011; Dynoodt *et al*, 2013b; Ma *et al*, 2013). Consistent with our study, FSCN1 downregulation was shown to inhibit melanoma cell proliferation (Ma *et al*, 2013) and to promote invasion (Dynoodt *et al*, 2013b). Interestingly, FSCN1 expression levels appear to be related to the differentiation stage of melanocytes and transient FSCN1 expression in melanoblast precursors is required for their proliferation and migration, with FSCN1 knockout resulting in hypopigmentation in adult mice (Ma *et al*, 2013). Notably, miR‐145‐5p is also considered as a key regulator of the pigmentary process in melanocytes, a role mediated by the downregulation of pigmentation genes and melanosome trafficking components, including FSCN1 (Dynoodt *et al*, 2013a). These findings are in line with our data showing that FSCN1 downregulation drives phenotypic transition to a poorly differentiated cell state associated with very low expression of the master regulator of melanocyte differentiation and function, MITF. FSCN1 downregulation may thus be exploited by melanoma cells to generate lineage plasticity and revert to a poorly differentiated phenotype during drug adaptation.

The miR‐143/‐145 FSCN1 axis also directly modulates the dynamic crosstalk between the actin cytoskeleton and the ECM through the regulation of focal adhesion dynamics. This process is known to promote melanoma survival through FAK signaling and the ROCK pathway to induce acto‐myosin‐mediated contractile forces (Fedorenko *et al*, 2015; Hirata *et al*, 2015; Orgaz *et al*, 2020). The involvement of the miR‐143/‐145 cluster is also linked to a fine‐tuning of mechanotransduction pathways. Enhanced YAP and MRTFA nuclear translocation reinforces the fibrotic‐like phenotype promoted by the cluster and probably facilitates resistance acquisition, as previously demonstrated for these mechanotransducers (Girard *et al*, 2020; Misek *et al*, 2020; Orgaz *et al*, 2020). Interestingly, MRTFA has been involved in the transcriptional regulation of miR‐143 and miR‐145 expression (Cordes *et al*, 2009; Xin *et al*, 2009; Long & Miano, 2011), suggesting that this transcriptional state might be further stabilized by a positive feedback loop. Such regulatory loops between miRNAs and transcription factors have been previously described in the establishment and maintenance of melanoma phenotypic states (Boyle *et al*, 2011; Li *et al*, 2020).

Despite the ability of FSCN1 downregulation to mimic the main functional effects observed by the ectopic expression of the miR‐143/‐145 cluster, we do not exclude the contribution of others targets in the acquisition of the mesenchymal resistant phenotype promoted by the cluster. FSCN1 knockdown failed to reproduce the global ECM signature reprogramming induced by the miR‐143/‐145 cluster. miRNA target prediction tools identified a plethora of genes involved in cell cycle regulation, DNA damage response, inflammatory pathways, and actin‐SRF regulatory network that need to be fully investigated in this context.

We conclude that our work opens new therapeutic avenues to prevent or delay the onset of resistance to targeted therapy in melanoma. Our findings provide a rationale for designing clinical trials with nintedanib and potentially other anti‐fibrotic agents to enhance treatment efficacy in BRAF‐mutated melanoma patients. We also bring an original mechanism of action directly linking the inhibition of the BRAF oncogenic pathway with the induction of the miR‐143/‐145 FibromiR cluster promoting the acquisition of a drug‐resistant, undifferentiated, and mesenchymal‐like cell state (Fig 8). Finally, we propose the cluster as a new promising biomarker or druggable target to overcome non‐genetic processes of phenotypic plasticity‐driven therapeutic resistance.

## Materials and Methods

### Cell lines and reagents

Isogenic pairs of vemurafenib‐sensitive and vemurafenib‐resistant melanoma cells (M229, M238, M249) were provided by R. Lo. UACC62 vemurafenib‐sensitive (UACC62P) and vemurafenib‐resistant melanoma cells (UACC62R) were provided by Neubig's laboratory. 1205Lu cells were obtained from Rockland. YUMM1.7 mouse melanoma cells were a kind gift from M. Bosenberg (Meeth *et al*, 2016). A375DR melanoma cells were provided by S. Shen. Cells were cultured in Dulbecco's modified Eagle's medium (DMEM) supplemented with 7% FBS (Hyclone) and 1% penicillin/streptomycin. Resistant cells were continuously exposed to 1 μM of vemurafenib. Cell lines were routinely tested for the absence of Mycoplasma by PCR.

Short‐term cultures of patient melanoma cells MM034 and MM099 were generated in the laboratory of Pr G. Ghanem. Culture reagents were purchased from Thermo Fisher Scientific. Vemurafenib (PLX4032), trametinib (GSK1120212), cobimetinib (SB431542, GSK690693), nintedanib (BIBF1120), and staurosporine were from Selleckchem. CP673451 was purchased from Tocris Bioscience. Recombinant human TGF‐β1 was obtained from ImmunoTools. Recombinant human PDGF‐BB was purchased from Peprotech.

Information on all reagents used is provided in Appendix Tables S4–S6.

### *In vivo* experiments

*In vivo* experiments were performed on 6‐week‐old female C57BL/6 mice (Janvier Labs: https://www.janvier‐labs.com/). Animal housing was carried out in the “Centre Méditerranéen de Médecine Moléculaire” in accordance with the Institutional Animal Care and the local ethical committee and within the context of approved project applications (CIEPAL‐Azur agreement NCE/2018‐483). 4 × 105 YUMM1.7 cells were injected in both flanks of C57BL/6 mice. Tumors were measured with caliper, and treatments were started when the tumors reached a volume of 0.1 cm^3^, after randomization of mice into control and test groups. Vemurafenib (30 mg/kg), trametinib (0.3 mg/kg), and nintedanib (50 mg/kg) were administered by oral gavage three times per week. Control mice were treated with vehicle only. Animals were sacrificed when the tumors reached a volume of 1 cm3. After animal sacrifice, tumors were dissected, weighed, and snap‐frozen in liquid nitrogen for RNA or protein extraction and immunofluorescence analysis (embedded in OCT from Tissue‐Tek). Tumors for picrosirius red staining were fixed in formalin. Melanoma cell‐derived xenograft experiments performed on 6‐week‐old female athymic nude nu/nu mice were described in Girard *et al* (2020). Melanoma patient‐derived xenograft models were established by TRACE (PDX platform; KU Leuven) using tissue from melanoma patients undergoing surgery at the University Hospitals KU Leuven. Written informed consent was obtained from all patients, and all procedures were approved by the UZ Leuven Medical Ethical Committee (S54185/S57760/S59199) and carried out in accordance with the principles set out in the WMA Declaration of Helsinki and the Department of Health and Human Services Belmont Report.

### Cell transduction

A DNA sequence containing the miR‐143/145 cluster was cloned into a pLX307 vector by Sigma‐Aldrich. The vector used for FSCN1 overexpression is described in Scott *et al*, 2011. Lentiviral particles were produced by the PVM Vectorology Platform in Montpellier, France. Melanoma cells were transduced as follows. After 20‐min incubation of melanoma cells with lentiviral particles diluted in OptiMEM, complete medium (7% FBS) was added to the cells. Forty‐eight hours after transduction, the process of antibiotic selection was started. For cells transduced for the miR‐143/‐145 cluster overexpression, 1 μg/ml of puromycin was administered every 48 h. For cells transduced for FSCN1 overexpression, 2 μg/ml of blasticidin was administered every 48 h. Experiments were performed starting 2 weeks after the beginning of antibiotic selection.

### RNAi studies

Non‐targeting control and FSCN1 siRNA duplexes were designed by Sigma‐Aldrich and used at a final concentration of 100 nM. Transfection was performed using Lipofectamine RNAiMAX (Life Technologies), according to the manufacturer's instructions. Cells were analyzed 72 h post‐transfection.

### miRNA overexpression and inhibition

Pre‐miRNA‐143‐3p and pre‐miRNA‐145‐5p and control miRNA (miR‐neg#1) were purchased from Ambion. LNA‐based miRNA inhibitors vs. miR‐143‐3p and miR‐145‐5p and the respective control (negative control A) were purchased from Qiagen. Pre‐miRNAs were used at a final concentration of 30 nM, and LNA inhibitors, at a final concentration of 50 nM. Transfection was performed using Lipofectamine RNAiMAX (Life Technologies), according to the manufacturer's instructions. Cells were analyzed 72 h post‐transfection unless otherwise stated.

### Luciferase assay

Molecular constructs for luciferase assay were made in psiCHECK‐2 vectors from Promega by cloning upstream of the Renilla luciferase gene annealed oligonucleotides based on the 3′UTR of target genes. HEK239 cells were plated on 96‐well plates and co‐transfected with 0.2 μg of psiCHECK‐2 plasmid constructs and 10 nM of pre‐miRNAs (miR‐143‐3p, miR‐145‐5p) or control pre‐miRNA. Transfections were performed using Lipofectamine 3000, following the manufacturer's instructions. Firefly and Renilla luciferase activities were measured using the Dual‐Glo Luciferase Assay Kit by Promega 48 h after transfection.

### Conditioned medium preparation

Medium conditioned by melanoma cells was harvested, centrifuged for 5 min at 2,500 *g*, and filtered with 0.22‐μm filters to eliminate cell debris.

### Tumor and cell RNA extraction

Total RNA was extracted from tumors and cell samples with the miRNeasy Mini Kit (Qiagen) according to the manufacturer's instructions.

### Real‐time quantitative PCR

#### Gene expression

Protocol using the StepOne (Applied Biosystems): 1 μg of extracted RNA was reverse‐transcribed into cDNA using the Multiscribe reverse transcriptase kit provided by Applied Biosystems. Primers were designed using PrimerBank or adopted from published studies. Gene expression levels were measured using Platinum SYBR Green qPCR Supermix (Fisher Scientific) and Step One thermocycler. Results from qPCR were normalized using the reference gene RPL32, and relative gene expression was quantified with the ΔΔCt method. Heatmaps describing gene expression fold changes were prepared using MeV software.

#### Protocol using the biomark HD system analysis (Fluidigm Corporation, USA)

cDNAs were prepared from 100 ng of RNA using Fluidigm Reverse Transcription Master Mix (Fluidigm PN 100‐647297). Following a pre‐amplification step (Fluidigm^®^ PreAmp Master Mix and DELTAgene™ Assay kits) and exonuclease I treatment, samples diluted in Eva‐Green^®^ Supermix with Low ROX were loaded with primer reaction mixes in 96.96 Dynamic Array™ IFCs. Gene expression was then assessed on a Fluidigm BioMark HD instrument. Data were analyzed with real‐time PCR analysis software (Fluidigm Corporation) and presented as relative gene expression according to the ΔΔ*C*_t_ method. Heatmaps depicting fold changes of gene expression were prepared using MeV software.

#### miRNAs expression

20 ng of extracted RNA was reverse‐transcribed into cDNA using the miRCURY LNA RT Kit (Qiagen). Mature miRNA expression levels were measured using the miRCURY LNA SYBR Green PCR Kit (Qiagen). Results from qPCR were normalized using miR‐16‐5p and relative gene expression was quantified with the ΔΔCt method. miRCURY LNA miRNA PCR assays for detecting miR‐143, miR‐145, and miR‐16 were purchased by Qiagen.

Information on primer sequences used in this study is provided in Appendix Tables S4 and S5.

### Western Blot analysis and antibodies

Whole‐cell lysates were prepared using RIPA buffer supplemented with protease and phosphatase inhibitors (Pierce, Fisher Scientific), briefly sonicated and centrifuged for 20 min, 4°C at 14,000 rpm. Whole‐cell lysates and conditioned media were separated using SDS‐PAGE and transferred into PVDF membranes (GE Healthcare Life Science) for Western blot analysis. Incubation of membranes with primary antibody was performed overnight. After washing, membranes were incubated with the peroxidase‐conjugated secondary antibody. A chemiluminescence system (GE Healthcare Life Science) was used to develop blots. HSP60 or HSP90 was used as loading control. For Western blot analysis of conditioned media experiments, Ponceau red staining was used as loading control.

Information on antibodies used in this study is provided in Appendix Table S6.

### Immunofluorescence and microscopy

Cell monolayers were grown on glass coverslips or collagen‐coated coverslips (10 μg/ml). After the indicated treatments, cells were washed in PBS, fixed in 4% PFA, permeabilized in PBS 0.3% Triton, and blocked in PBS 5% goat serum. Coverslips were then incubated overnight at 4°C with primary antibody diluted in PBS 5% goat serum. Following 1‐h incubation with Alexa Fluor‐conjugated secondary antibody, coverslips were mounted with Prolong antifade mounting reagent (Thermo Fisher Scientific). Nuclei were stained with Hoechst 33342 (Life Technologies). F‐actin was stained with Alexa Fluor 488 phalloidin (Fisher Scientific) or phalloidin‐iFluor 594 (Abcam) reagents. Coverslips were imaged using a wide‐field Leica DM5500B microscope.

### Fibrillar collagen imaging

Collagen in paraffin‐embedded tumors was stained with picrosirius red using standard protocols. Tumor sections were analyzed by polarized light microscopy as described in (Girard *et al* (2020). Images were acquired under polarized illumination using a light transmission microscope (Zeiss PALM, at 10× magnification). Fiber thickness was analyzed by the change in polarization color. Birefringence hue and amount were quantified as a percent of total tissue area using ImageJ software.

### Viability assay

After the indicated treatments, cells were stained with 0.04% crystal violet, 20% ethanol in PBS for 30 min. Following accurate washing of the plate, representative photographs were taken. The crystal violet dye was solubilized by 10% acetic acid in PBS and measured by absorbance at 595 nm.

### Proliferation assay

For real‐time analysis of cell proliferation, 3 × 10^4^ cells were plated in complete medium in triplicates on 12‐well plates. The IncuCyte ZOOM imaging system (Essen Bioscience) was used. Phase‐contrast pictures were taken every hour. Proliferation curves were generated using the IncuCyte cell proliferation assay software based on cell confluence.

### Cell cycle analysis

Cell cycle analysis was performed by flow cytometry analysis of cells stained with propidium iodide. After fixation in ice‐cold 70% ethanol, cells were stained with 40 μg/ml propidium iodide in PBS with 100 μg/ml RNAse A. The samples were then analyzed on a BD FACSCanto cytometer.

### Analysis of apoptosis by flow cytometry

Cell death was evaluated by flow cytometry following staining with Annexin V‐FITC and DAPI (eBioscience) according to the manufacturer's instructions. The samples were then analyzed on a BD FACSCanto cytometer.

### Migration and invasion assays

Migration properties of melanoma cells were tested using Boyden chambers containing polycarbonate membranes (8 μm pores transwell from Corning). After overnight starvation, 1 × 10^4^ cells were seeded on the upper side of the chambers placed on 24‐well plates containing 10% FBS medium for 24 h, unless otherwise stated, at 37°C in 5% CO_2_. At the end of the experiment, cells migrated on the lower side of the chambers were fixed in 4% paraformaldehyde, stained for 15 min with Hoechst, and imaged at the microscope (five random fields per well). Nuclei counting was performed using the ImageJ software. To assess invasion properties of melanoma cells, transwells were coated with Matrigel (1 mg/ml) and cell solution was added on the top of the matrigel coating to simulate invasion through the extracellular matrix.

### Immunofluorescence analysis

Cell area was measured on cells stained for F‐Actin using ImageJ. The nuclear/cytosolic ratio of YAP or MRTF was quantified by measuring the nuclear and cytosolic fluorescence intensity using ImageJ. The Hoechst staining was used to define nuclear versus cytosolic regions. Focal adhesions were quantified using ImageJ. Pictures were subjected to background subtraction (rolling: 10) before analysis; then, “default threshold” was applied, followed by “analyze particles of object with a size 0.20 and infinity” to analyze the number of objects and their area. The number of focal adhesions was normalized to the total cell area.

### Microarray gene expression analysis

Total RNA integrity was tested with the Agilent BioAnalyser 2100 (Agilent Technologies). After labeling RNA samples with the Cy3 dye using the low RNA input QuickAmp Kit (Agilent) following the manufacturer's instruction, labeled cRNA probes were hybridized on 8 × 60K high‐density SurePrint G3 gene expression human Agilent microarrays.

### RNA‐sequencing

Short reads: Libraries were generated from 500 ng of total RNAs using TruSeq Stranded Total RNA Kit (Illumina). Libraries were then quantified with KAPA Library Quantification Kit (Kapa Biosystems) and pooled. 4 nM of this pool was loaded on a high output flow cell and sequenced on an Illumina NextSeq500 sequencer using 2 × 75 bp paired‐end chemistry. Reads were aligned to the human genome release hg38 with STAR 2.5.2a as previously described (Savary *et al*, 2019).

Nanopore long reads: libraries were prepared according to the PCR‐cDNA Barcoding protocol (SQK – PCB109). Briefly, 50 ng of total RNA was reverse‐transcribed, barcoded, and amplified by PCR (17 cycles) and sequencing adapters were added. The two barcoded libraries (CARMN RNA 238R and CARMN RNA 238S) were mixed 1:1, and 110 fmol was loaded on a PromethION flow cell (FLO‐PRO002). Reads were processed with the FLAIR pipeline (https://doi.org/10.1038/s41467‐020‐15171‐6). Raw reads were aligned to hg38 with minimap2 (version 2.17‐r941). Misaligned splice sites were corrected according to the GENCODE v.35 annotations. High‐confidence isoforms were defined after grouping corrected reads of all samples sharing same unique splice junctions, by selecting for each group a representative isoform with confident TSS/TES and supported by more than three reads. Selected isoforms were quantified using minimap2 in each sample. Differential isoform expression and alternative splicing event significance were tested without replicates using ad‐hoc scripts provided on the Brook's lab GitHub (https://github.com/BrooksLabUCSC/FLAIR).

Statistical analysis and biological theme analysis: Microarray data analyses were performed using R (http://www.r‐project.org/). Quality control of expression arrays was performed using the Bioconductor package arrayQualityMetrics and custom R scripts. Additional analyses of expression arrays were performed using the Bioconductor package limma. Briefly, data were normalized using the quantile method. No background subtraction was performed. Replicated probes were averaged after normalization and control probes removed. Statistical significance was assessed using the limma moderated *t*‐statistic. Quality control of RNA‐seq count data was assessed using in‐house R scripts. Normalization and statistical analysis were performed using Bioconductor package DESeq2. All *P*‐values were adjusted for multiple testing using the Benjamini–Hochberg procedure, which controls the false discovery rate (FDR). Differentially expressed genes were selected based on an adjusted *P*‐value below 0.05. Enrichment in biological themes (molecular function, upstream regulators, and canonical pathways) was performed using Ingenuity Pathway Analysis software (http://www.ingenuity.com/).

### miRNA target analysis

MiRonTop is an online java web tool (http://www.genomique.info/) (Le Brigand *et al*, 2010) integrating whole‐transcriptome expression data to investigate whether specific miRNAs are involved in a specific biological system. MiRonTop classifies transcripts into two categories (“Upregulated” and “Downregulated”), based on thresholds for expression level, differential expression, and statistical significance. It then analyzes the number of predicted targets for each miRNA, according to the prediction software selected (Targetscan, exact seed search, TarBase).

### Statistical analysis

Statistical analysis was performed using GraphPad Prism. Unpaired two‐tailed Student's *t*‐test or the unpaired two‐tailed Mann–Whitney test was used for statistical comparison between two groups. For comparisons between multiple groups, one‐way ANOVA followed by Bonferroni's *post hoc* tests was used. For statistical analysis of cell confluence live imaging, two‐way ANOVA was used. For statistical analysis of Kaplan–Meier curves, the log rank (Mantel–Cox) test was used. Results are given as mean ± SEM or mean ± SD. Exact *P*‐values obtained for the statistical analyses provided in the main figures are available in Appendix Table S7.

## Author contributions

**Serena Diazzi:** Conceptualization; Formal analysis; Investigation; Methodology; Writing—original draft; Writing—review and editing. **Alberto Baeri:** Formal analysis; Investigation; Methodology. **Julien Fassy:** Formal analysis; Investigation; Methodology. **Margaux Lecacheur:** Investigation; Methodology. **Oskar Marin‐Bejar:** Data curation; Investigation. **Christophe A Girard:** Investigation; Methodology. **Lauren Lefevre:** Investigation. **Caroline Lacoux:** Investigation. **Marie Irondelle:** Formal analysis. **Carine Mounier:** Investigation. **Marin Truchi:** Formal analysis. **Marie Couralet:** Investigation. **Mickael Ohanna:** Investigation. **Alexandrine Carminati:** Investigation. **Ilona Berestjuk:** Investigation. **Frederic Larbret:** Investigation. **David Gilot:** Resources; Writing—review and editing. **Georges Vassaux:** Investigation; Writing—review and editing. **Jean‐Christophe Marine:** Resources; Writing—review and editing. **Marcel Deckert:** Conceptualization; Formal analysis; Supervision; Funding acquisition; Methodology; Writing—review and editing. **Bernard Mari:** Conceptualization; Formal analysis; Supervision; Funding acquisition; Methodology; Writing—original draft; Project administration; Writing—review and editing. **Sophie Tartare‐Deckert:** Conceptualization; Formal analysis; Supervision; Funding acquisition; Methodology; Writing—original draft; Project administration; Writing—review and editing.

In addition to the CRediT author contributions listed above, the contributions in detail are:

SD, MD, BM, and ST‐D conceived and designed the study. SD, AB, JF, CAG, ML, GV, MD, BM, and ST‐D developed methodology. SD, AB, JF, ML, OM‐B, CG, LL, CL, CM, MT, MC, MO, AC, IB, FL, GV, and J‐CM acquired data. SD, AB, JF, MI, FL, MD, BM, and ST‐D analyzed and interpreted the data. SD, BM, and ST‐D wrote the original draft. SD, DG, GV, J‐CM, MD, BM, and ST‐D wrote, reviewed, and edited the manuscript. MD, BM, and ST‐D provided administrative, technical, or material support. MD, BM, and ST‐D supervised the study.

## Supporting information



Appendix

Expanded View Figures PDF

Source Data for Figure 1

Source Data for Figure 2

Source Data for Figure 3

Source Data for Figure 4

Source Data for Figure 5

Source Data for Figure 6

Source Data for Figure 7

## Data Availability

Expression datasets that support the findings of this study have been deposited in the Gene Expression Omnibus SuperSerie record GSE171883 (http://www.ncbi.nlm.nih.gov/geo/query/acc.cgi?acc=GSE171883) containing 3 distinct datasets under the following accession codes:
‐ Dataset 1: GSE171880 (http://www.ncbi.nlm.nih.gov/geo/query/acc.cgi?acc=GSE171880). Effect of miR‐143‐3p or miR‐145‐5p mimics overexpression in M238P cells (microarrays). ‐ Dataset 2: GSE171881 (http://www.ncbi.nlm.nih.gov/geo/query/acc.cgi?acc=GSE171881). RNA‐Seq analysis of M238P stably expressing miR‐143/‐145 cluster. ‐ Dataset 3: GSE171882 (http://www.ncbi.nlm.nih.gov/geo/query/acc.cgi?acc=GSE171882). Transcriptome analysis of M238R versus M238P using nanopore long‐read sequencing.
