# Supplementary material for: Blockade of the pro‐fibrotic reaction mediated by the miR‐143/‐145 cluster enhances the responses to targeted therapy in melanoma
Source: EMBO Mol Med. 2022 Feb 14;14(3):e15295. doi: 10.15252/emmm.202115295 (PMC8899916; doi:10.15252/emmm.202115295)
Supplement: Supplementary file 1 — Appendix [file EMMM-14-e15295-s001.pdf]

Appendix

Table of contents

Appendix Figure S1
Appendix Figure S2
Appendix Figure S3
Appendix Figure S4
Appendix Figure S5
Appendix Figure S6
Appendix Figure S7
Appendix Table S1
Appendix Table S2
Appendix Table S3
Appendix Table S4
Appendix Table S5
Appendix Table S6
Appendix Table S7
References

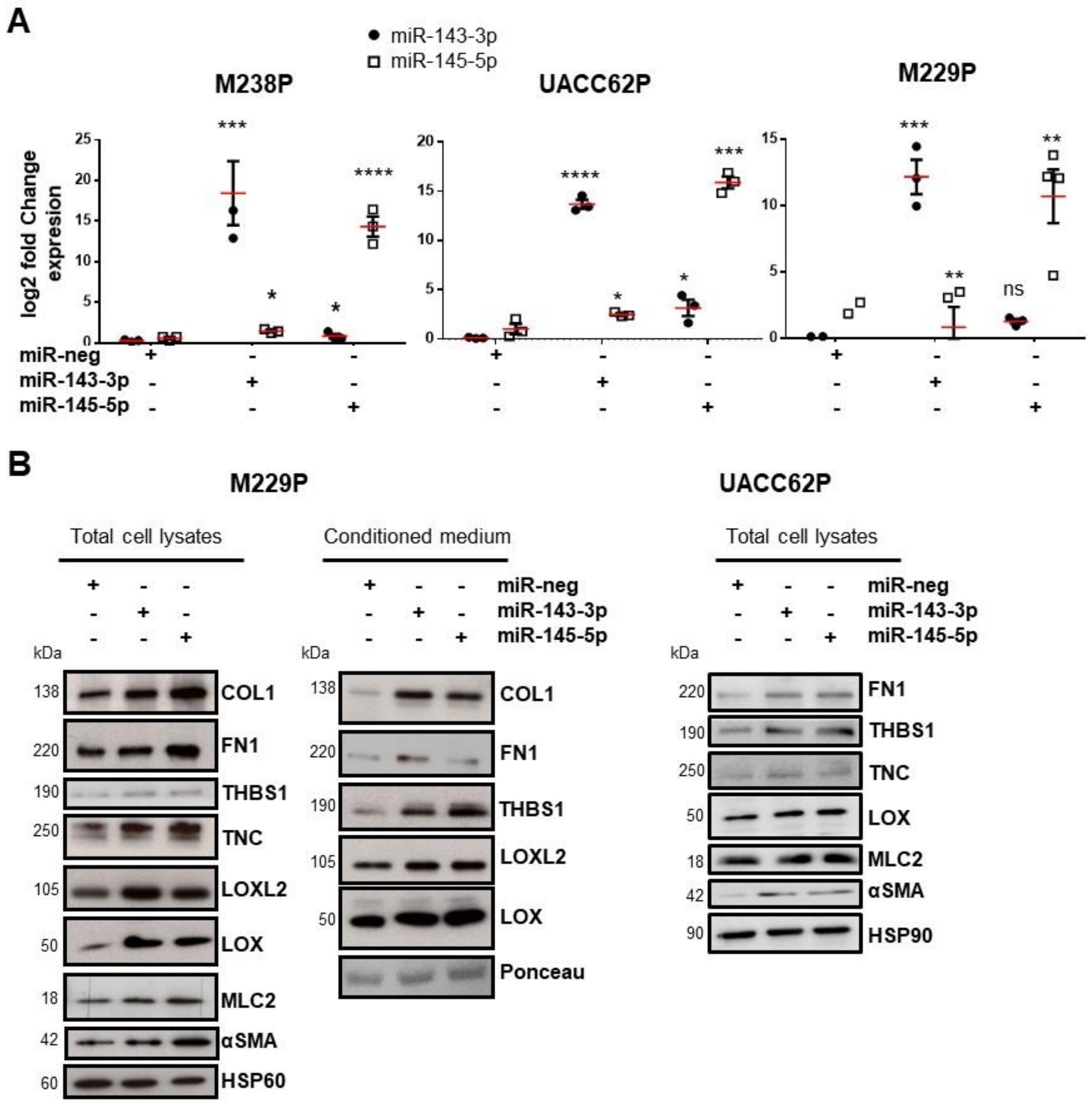

Appendix Figure S1. The miR-143/-145 cluster plays a role in ECM reprogramming. (A) qPCR analysis showing the level of miR-143-3p and miR-145-5p expression after transient transfection of miRNAs mimics (72 h, 30 nM) in 3 distinct melanoma cell lines (M238P, UACC62P, M229P). Data is represented as mean ± SE from a triplicate representative of at least 3 independent experiments. Paired Student t-test has been used for statistical analysis. *P≤0.05 **P≤0.01 ***P≤0.001 ****P≤0.0001 (**B**) Immunoblot analysis of ECM remodeling markers on total cell lysates (M229P and UACC62P) or conditioned medium (M229P) from parental cells transfected with the indicated mimics.

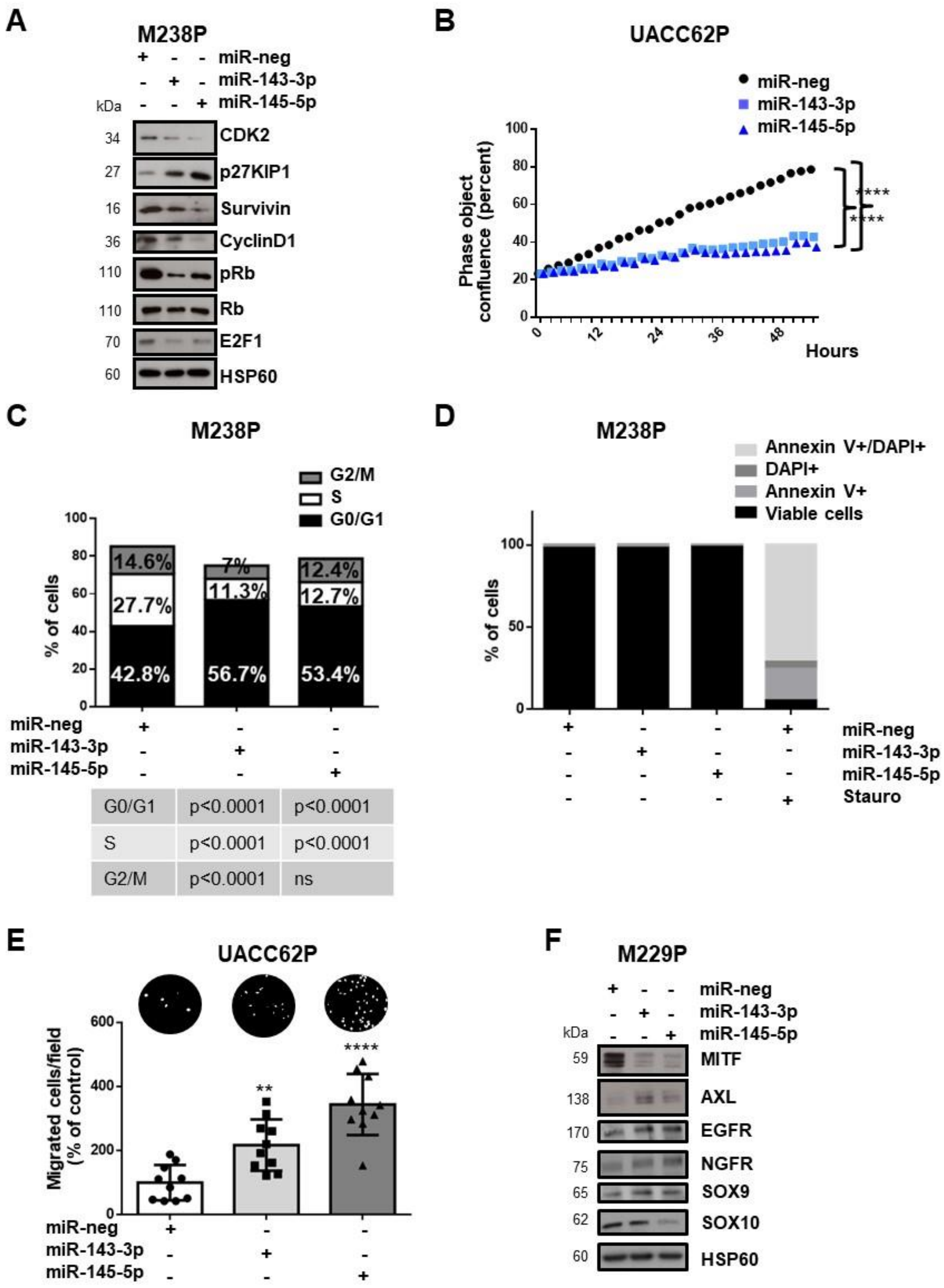

Appendix Figure S2. The miR-143/-145 cluster drives melanoma cell plasticity and dedifferentiation. Melanoma cells were transfected with control (miR-neg), miR-143 or miR-145 mimics (72 h, 30 nM). **(A)** Immunoblot analysis of cell cycle markers on lysates from M238P cells. **(B)** Proliferation curves of parental cells (UACC62P) following transient transfection with miRNA mimics. Time-lapse analysis of cells has been performed with the IncuCyte system. Graph shows quantification of cell confluence. Two-way ANOVA analysis has been used for statistical analysis. ****$P \leq 0.0001$. **(C)** Cell cycle distribution of cells cultured in the different conditions. Histograms represent the percentage of cells in different phases of the cell cycle. **(D)** Flow cytometry analysis of cell death (Annexin V/DAPI labelling) in M238P cells following transient transfection with miRNAs mimics or staurosporine treatment (Stauro, 1 µg/mL, 24 h). Histograms represent the percentage of non-stained/stained cells. **(E)** Migration assay of melanoma cells (UACC62P) following transient transfection with miRNA mimics in Boyden chambers. Representative images show migration in control and miR-143-3p or miR-145-5p transfected cells. The histogram represents the quantitative determination of data obtained using ImageJ software. Paired Student t-test has been used for statistical analysis. **$P \leq 0.01$, ****$P \leq 0.0001$. **(F)** Immunoblot analysis of phenotype switch markers on lysates from M229P cells transfected with the indicated mimics.

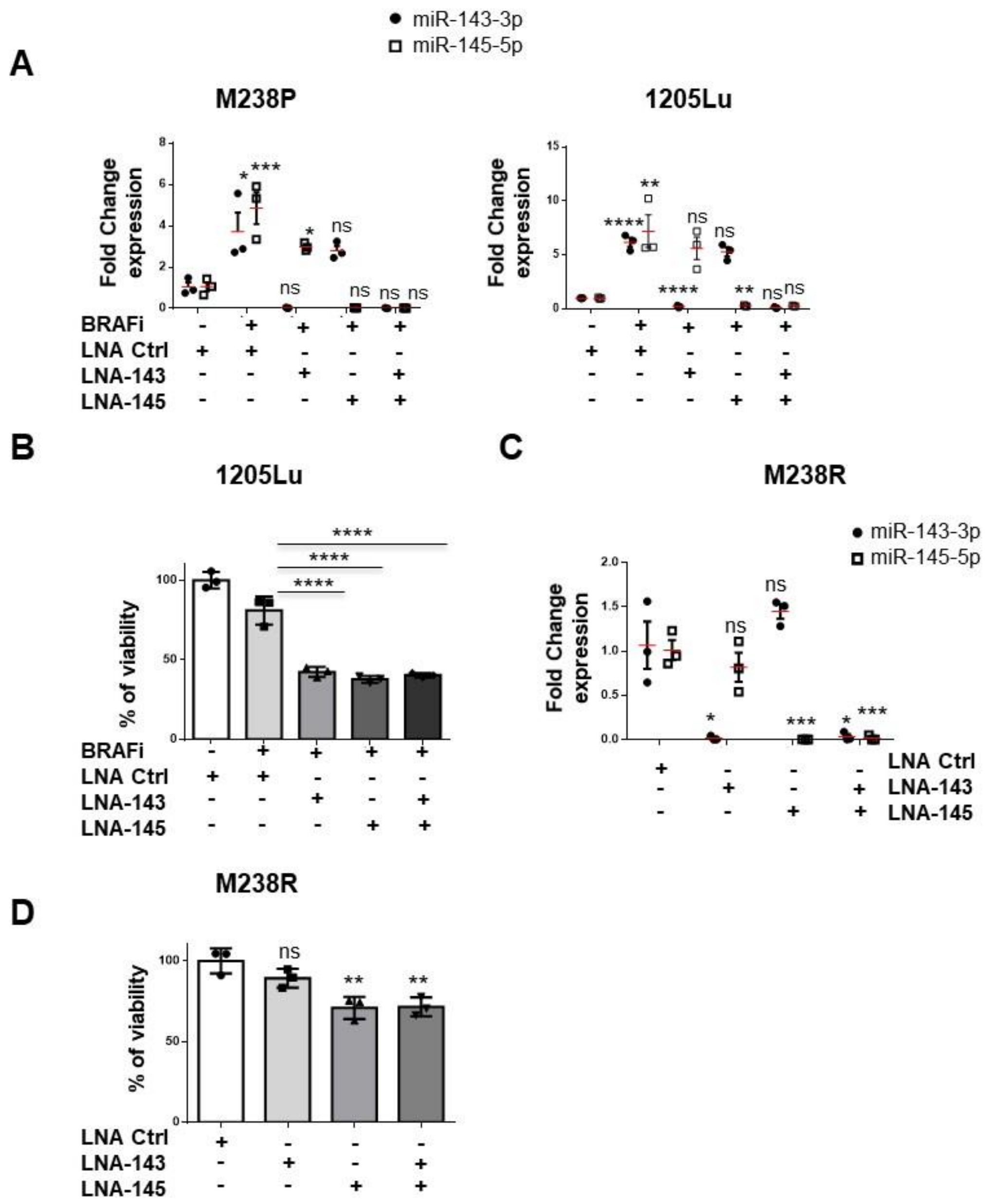

Appendix Figure S3. miR-143 and miR-145 inhibition reverses the adaptive response of melanoma cells to MAPK pathway inhibition. Cells were treated with BRAFi (Vemurafenib, 3 μM) in the presence or the absence of anti-miR inhibitors (50 nM) for 72 h. **(A)** RT-qPCR analysis of miR-143-3p and miR-145-5p expression in parental cells (M238P and 1205Lu) treated with the different combinations of inhibitors. Data is represented as mean ± SE from a triplicate representative of at least 3 independent experiments. Paired Student t-test has been used for statistical analysis**.** *P≤0.05, **P≤0.01, ***P≤0.001, ****P≤0.0001. **(B)** Crystal violet viability assay of 1205Lu cells treated 72 h with the indicated combinations of inhibitors. Data is represented as mean ± SD from a triplicate representative of at least 3 independent experiments. One-way ANOVA has been used for statistical analysis. ****P≤0.0001. **(C)** RT-qPCR analysis of miR-143-3p and miR-145-5p expression in resistant M238R cells treated with the different combinations of inhibitors. Data is represented as mean ± SE from a triplicate representative of at least 3 independent experiments. Paired Student t-test has been used for statistical analysis**.** *P≤0.05, ***P≤0.001. **(D)** Crystal violet viability assay of M238R cells treated 72 h with the indicated combinations of inhibitors (50 nM). Data is represented as mean ± SD from a triplicate representative of at least 3 independent experiments. One-way ANOVA has been used for statistical analysis. **P≤0.01.

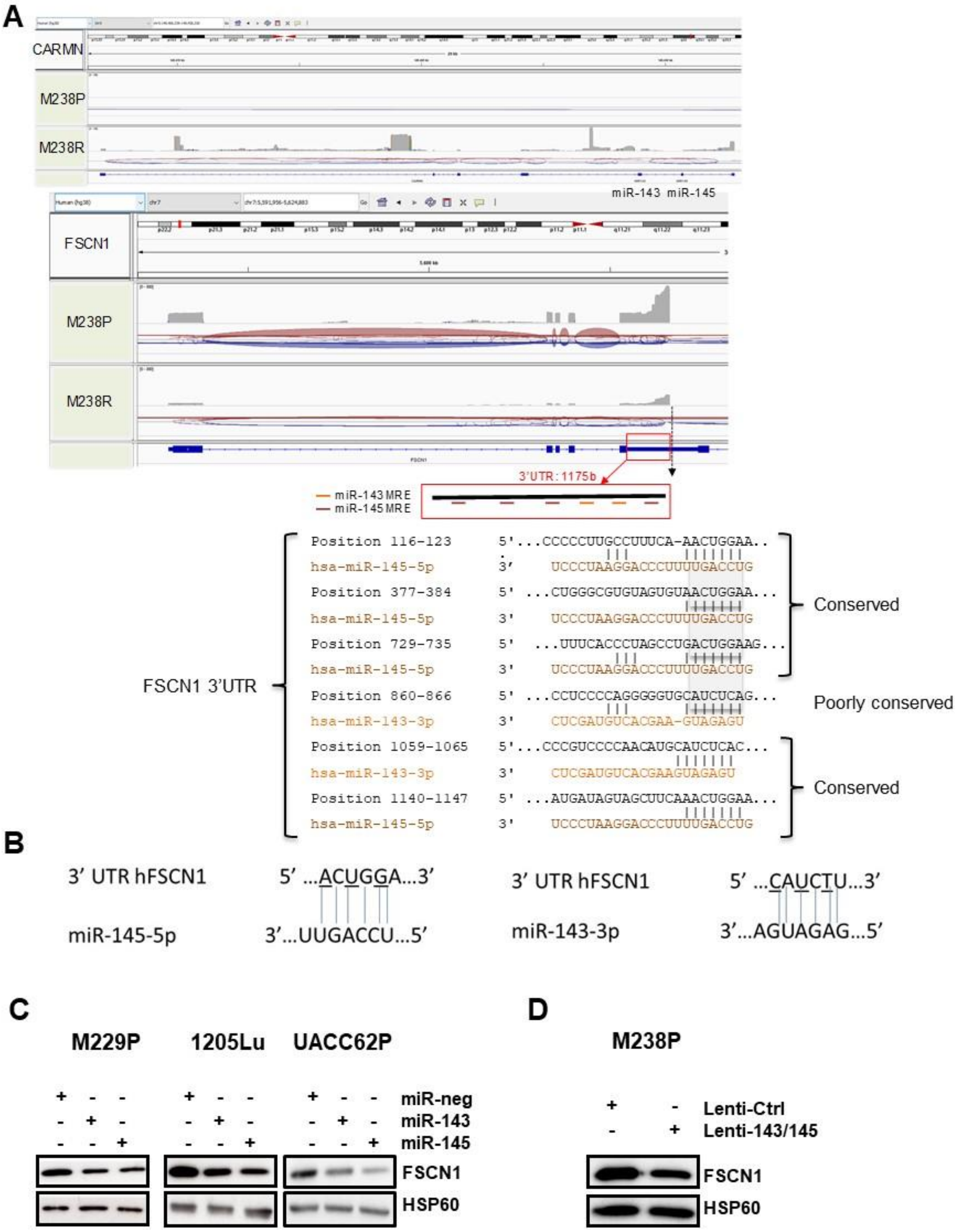

Appendix Figure S4. Characterization of the transcripts produced from the miR-143/-145 cluster and FSCN1 loci in parental and mesenchymal resistant melanoma cells. (A) Screenshot from Integrative Genomic Viewer (IGV) displaying nanopore long-reads RNA-Seq data of the miR-143/-145 cluster / CARMN and FSCN1 loci in M238P and M238R cells (dataset 3, n=1). A strong increase of total reads associated with the CARMN transcripts in M238R compared to M238P cells is shown while the FSCN1 transcript shows the opposite pattern. The red box highlights the FSCN1 3'UTR containing 2 and 4 predicted sites for miR-143-3p and miR-145-5p, respectively. The sequence, pairing and conservation are shown for each predicted site. **(B)** Sequence of h*FSCN1* 3'UTR miR-143 or miR-145 recognition elements and pairing with miR-143 or miR-145 seeds. Bases mutated in the plasmid used for luciferase assay are underlined. **(C-D)** Western Blot analysis of FSCN1 expression in M229P, 1205Lu, and UACC62P cells transfected with the indicated miRNA mimics (72 h, 30 nM) (**C**) and in M238P cells transduced with the indicated construct **(D)**.

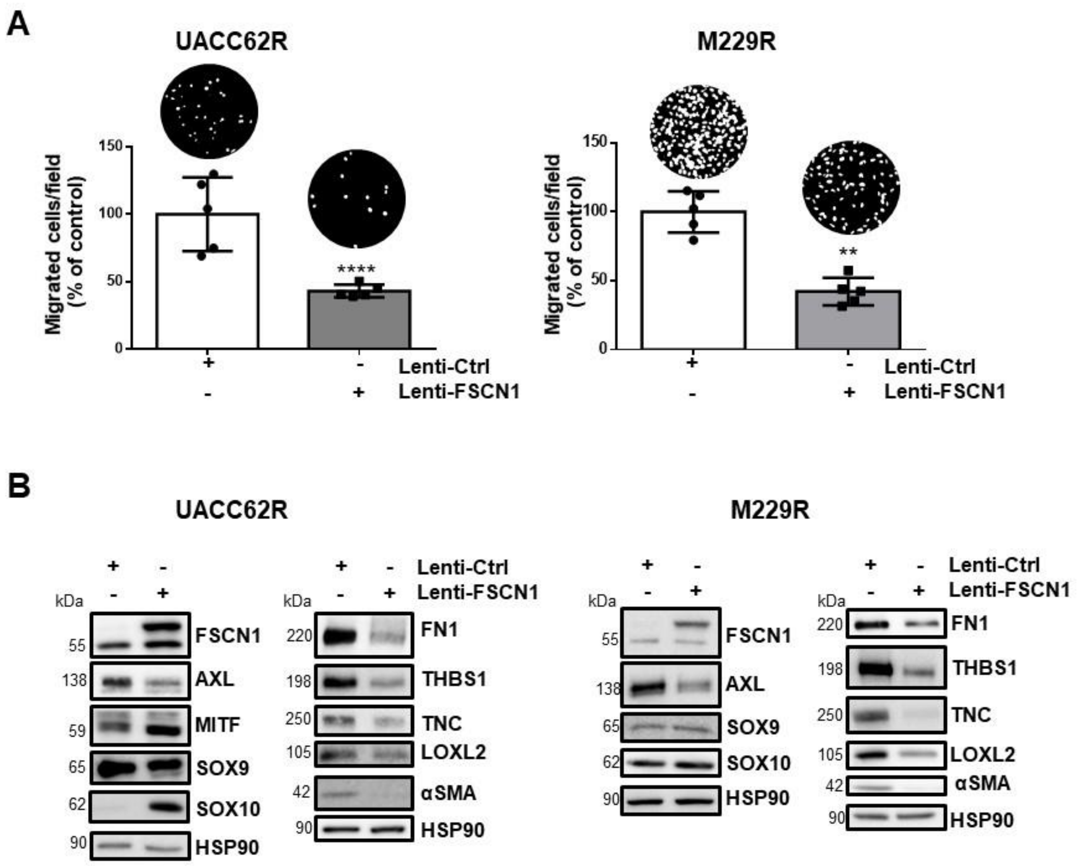

Appendix Figure S5. FSCN1 restoration promotes the switch of melanoma cells toward a differentiated cell state. (A-B) Cells were transduced with a control or a FSCN1 lentiviral construct. **(A)** Effect of FSCN1 overexpression on cell migration (Boyden chambers). Representative images and quantitative determination of data obtained using ImageJ software. Paired Student t-test has been used for statistical analysis. **P≤0.01, ****P≤0.0001. **(B)** Immunoblot analysis of phenotype switch markers and ECM remodeling markers on cell lysates from control and FSCN1 overexpressing resistant cells (UACC62R, M229R).

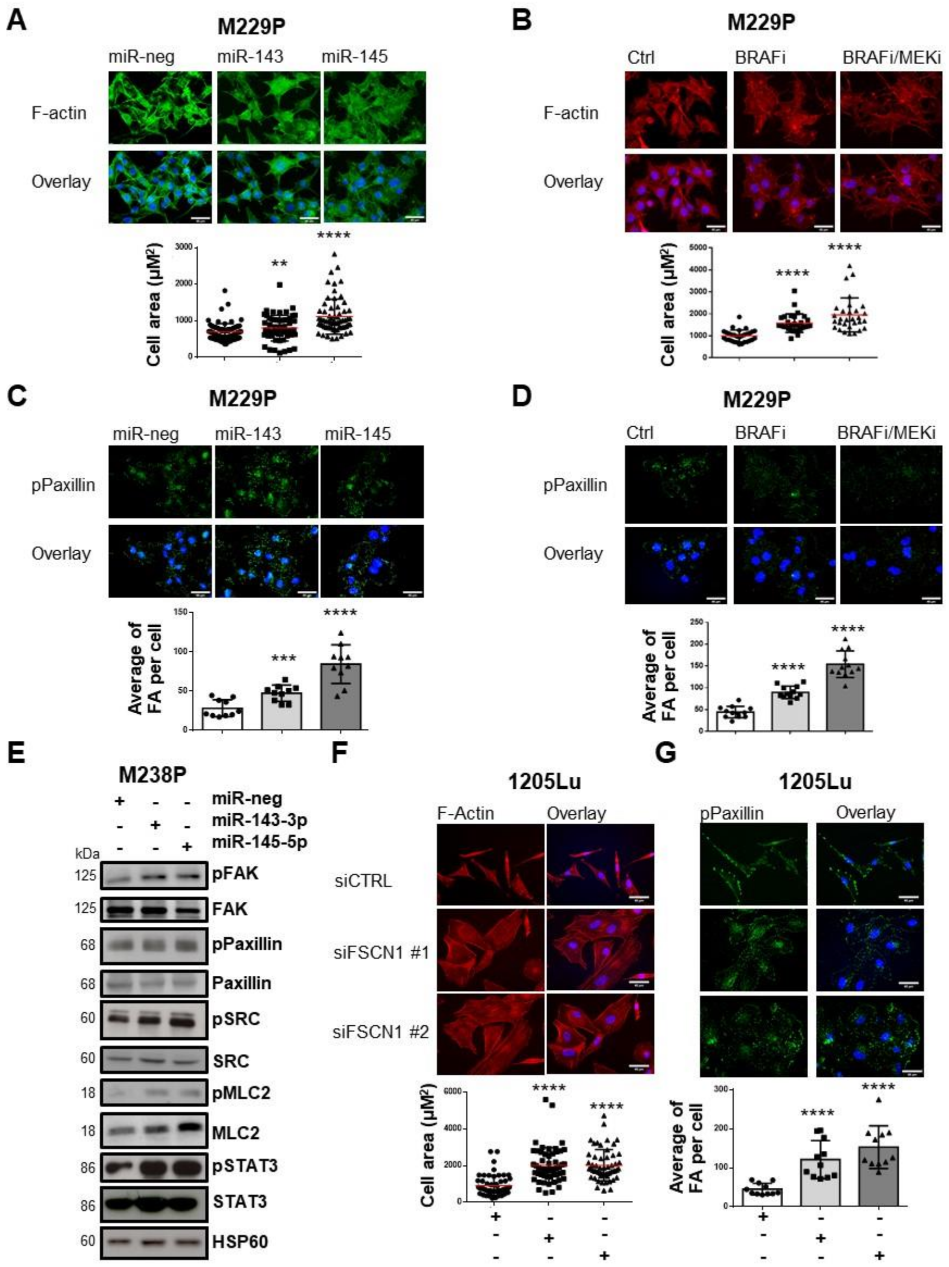

Appendix Figure S6. The miR-143-/-145 cluster / FSCN1 axis regulates actin cytoskeleton dynamics. (A-C) M229P cells were transfected with miR-143-3p, miR-145-5p or a control mimic (72 h, 30nM). **(B-D)** M229P cells were treated 72 h with BRAFi (Vemurafenib 3 µM) or a combination of BRAFi (0.5 µM) and MEKi (Trametinib, 1 µM). **(A-B)** Images and quantification of cell area in cells stained for F-actin (green) and nuclei (blue). Data is represented as scatter plot with mean ± SD (n≥30 cells per condition). Mann-Whitney U test has been used for statistical analysis. **P≤0.01, ****P≤0.0001. Scale bar 40 µm. **(C-D)** Images and quantification of focal adhesions (FA) number in cells stained for pPaxillin (green) and nuclei (blue). Focal adhesions number is represented as mean ± SD (n≥30 cells per condition). Each point represents the average number of focal adhesions per cell calculated for each field. Paired Student t-test has been used for statistical analysis. ***P≤0.001, ****P≤0.0001. Scale bar 40 µm. **(E)** Immunoblot analysis of focal adhesion components and cytoskeleton-related pathways in M238P cells transfected with the different mimics. (30 nM, 72 h). **(F-G)** 1205Lu cells were transfected with two different sequences of siRNAs vs FSCN1 or with a control siRNA (72 h, 100 nM). **(F)** Images and quantification of cell area in cells stained for F-Actin (red) and nuclei (blue). Data is represented as scatter plot with mean ± SD. (n≥30 cells per condition). Mann-Whitney U test has been used for statistical analysis. ****P≤0.0001. Scale bar 40 µm. **(G)** Images and quantification of focal adhesions (FA) number in cells stained for pPaxillin (green) and nuclei (blue). Focal adhesions number is represented as mean ± SD (n≥30 cells per condition). Each point represents the average number of focal adhesions per cell calculated for each field. Mann-Whitney U test has been used for statistical analysis. ****P≤0.0001. Scale bar 40 µm.

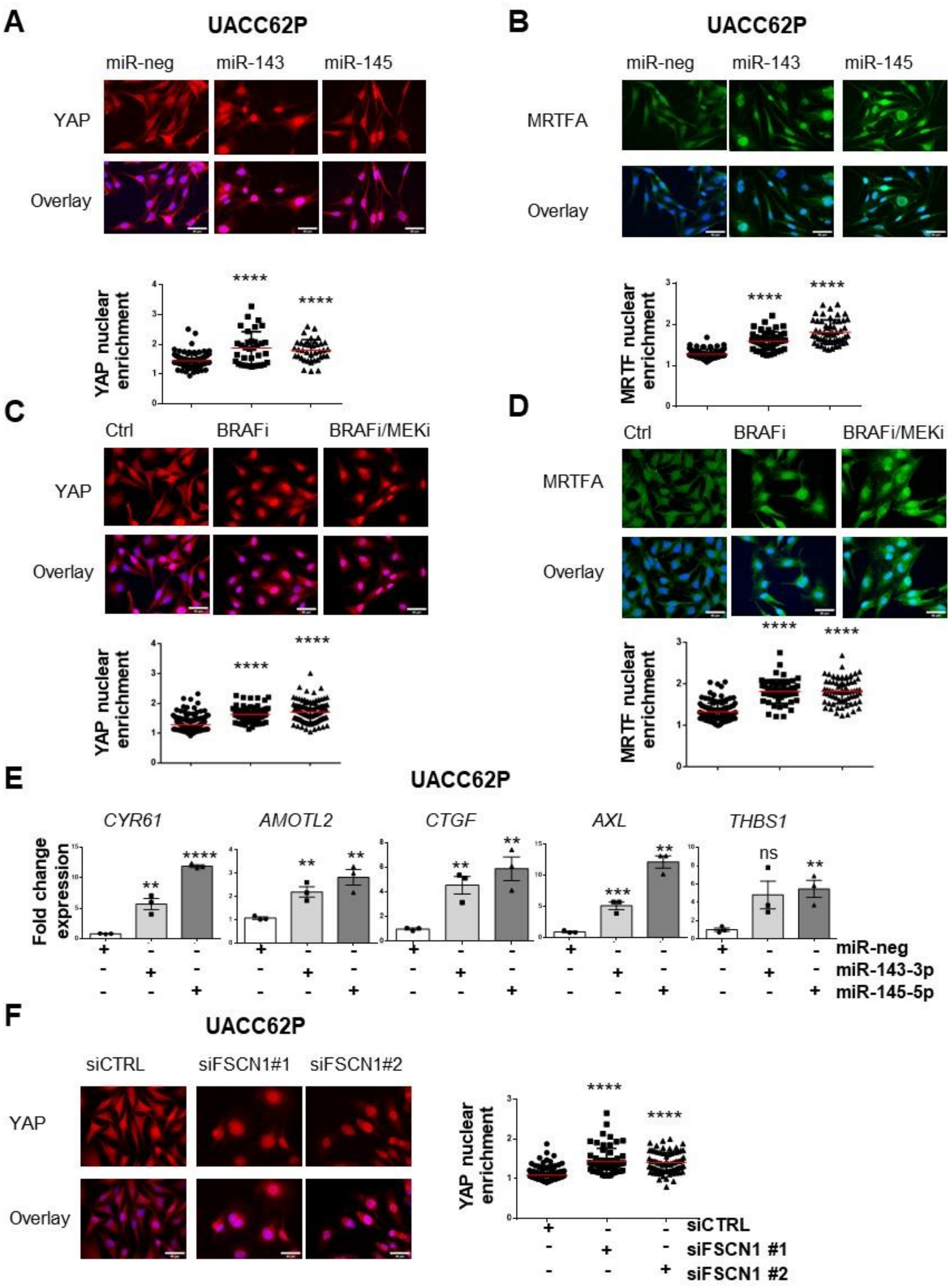

Appendix Figure S7. The miR-143-/-145 cluster / FSCN1 axis regulates mechanopathways. (A-B) UACC62P cells were transfected with miR-143-3p, miR-145-5p or a control mimic (72 h, 30 nM). Effect of miR-143 or miR-145 overexpression on YAP **(A)** or MRTFA **(B)** nuclear translocation assessed by immunofluorescence. Cells were stained for YAP (red) or MRTFA (green) and nuclei (blue). **(C-D)** UACC62P cells were treated 72 h with BRAFi (Vemurafenib, 3 µM) or a combination of BRAFi (0.5 µM) and MEKi,(Trametinib, 1 µM). Effect of MAPKi on YAP **(C)** or MRTFA **(D)** nuclear translocation by immunofluorescence. Cells were stained for YAP (red) or MRTFA (green) and nuclei (blue). **(A-B-C-D)** Data are represented as scatter plot with mean ± SD (n≥30 cells per condition). Each point represents the nuclear/cytoplasm ratio. Mann-Whitney U test has been used for statistical analysis. ****P≤0.0001. Scale bar 40 µm. **(E)** Effect of miR-143 or miR-145 overexpression (72h, 30 nM) on the expression of MRTFA/YAP target genes assessed by RT-qPCR. Data are normalized to the expression in control cells. Data is represented as mean ± SE from a triplicate representative of at least 3 independent experiments. Paired Student t-test has been used for statistical analysis. **P≤0.01**,** ***P≤0.001, ****P≤0.0001**.** **(F)** Effect of FSCN1 knockdown on YAP nuclear translocation assessed by immunofluorescence in UACC62P cells transfected with the indicated siRNA. Cells were stained for YAP (red) and nuclei (blue). Data are represented as scatter plot with mean ± SD (n≥30 cells per condition). Each point represents the nuclear/cytoplasm ratio. Mann-Whitney U test has been used for statistical analysis. ****P≤0.0001.

Appendix Table S1. List of themes corresponding to "Molecular functions" annotations associated with miR-143-3p and miR-145-5p mimics overexpression in human M238P melanoma cells identified by Ingenuity Pathway Analysis.

M238P cells were transfected with miR-143, miR-145 or a control mimic (72 h, 30 nM). Expression profiles were determined with Agilent whole genome microarrays (Dataset 1). Z-scores calculated for each pathway (miR-143-3p or miR-145-5p vs miR-Neg) are indicated. Significant pathways are shown in progressively brighter shades of blue (repression) and orange (activation) according to their significance.

Molecular Functions	- Log10 (P Val)	
	miR-143-3p	miR-145-5p
Migration of cells	12.13	15.13
Cell movement	11.08	14.26
Cell proliferation of tumor cell lines	11.80	11.09
Invasion of cells	10.24	8.80
Migration of tumor cell lines	10.46	7.65
Cell movement of tumor cell lines	8.93	9.02
Organization of cytoplasm	8.27	9.26
Organization of cytoskeleton	6.44	10.13
Necrosis	10.62	5.59
Apoptosis	10.07	5.72
Cell death of tumor cell lines	10.28	4.99
Invasion of tumor cell lines	9.31	5.86
Cell survival	8.75	5.23
Cell viability	8.90	4.96
Microtubule dynamics	4.54	9.04
Cell cycle progression	8.08	4.99
Apoptosis of tumor cell lines	6.70	4.56
Cell viability of tumor cell lines	6.02	4.61
Phosphorylation of protein	3.60	6.83
Sprouting	3.61	6.61
Formation of cellular protrusions	0.00	10.05
Colony formation of cells	6.12	3.70
Biosynthesis of amide	3.80	5.76
Migration of cancer cells	0.00	9.02
Migration of tumor cells	0.00	8.84
Cell movement of tumor cells	0.00	8.82
Interphase	8.15	0.00
DNA replication	7.64	0.00
Transmigration of cells	0.00	7.62
Arrest in interphase	6.86	0.00
G1/S phase transition	6.81	0.00
G1 phase	6.70	0.00
Mitosis	6.58	0.00
Metabolism of DNA	6.21	0.00
Synthesis of DNA	6.20	0.00
Damage of chromosomes	6.00	0.00
Metabolism of reactive oxygen species	5.44	0.00
Chromosomal aberration	5.37	0.00
Entry into interphase	5.30	0.00
Chromosomal instability	5.23	0.00
Arrest in mitosis	5.23	0.00
Synthesis of glycosaminoglycan	0.00	5.19
Secretion of molecule	0.00	5.16
Synthesis of lipid	0.00	5.16

Appendix Table S2. List of the main predicted targets for miR-143-3p and miR-145-5p significantly downregulated following mimics overexpression in human M238P melanoma cells using the bioinformatics tool miRonTop.

M238P cells were transfected with miR-143, miR-145 or a control mimic (48 h, 30 nM). Expression profiles were determined with Agilent whole genome microarrays (Dataset 1). Predicted targets were obtained using the bioinformatics tool miRonTop (https://www.genomique.info:8443/merge/index) (Le Brigand et al., 2010) based on the Targetscan database (conserved sites) (www.targetscan.org) (Agarwal et al., 2015). Av.Exp: logarithm (base 2) of the average intensity ; logFC: logarithm (base 2) of the ratio of miR-143-3p or miR-145-5p vs miR-Neg; Adj.pVal: Benjamini-Hochberg adjusted pValue. The thresholds used for the analysis are: Av.Exp >6, LogFC<-1 and adj.pVal <0.05.

miR-143-3p predicted targets			
Symbol	Av.Exp	LogFC	Adj.pVal
ABHD14A	11.92	-1.27	5.62E-05
ADD3	9.14	-1.20	3.48E-05
ASAP3	6.68	-1.29	3.10E-05
ATP6V1A	12.26	-1.17	4.49E-05
BCL2	6.29	-1.14	1.07E-04
CDK1	9.61	-1.14	5.44E-05
CNNM3	6.94	-1.32	3.35E-05
EFS	10.95	-1.35	2.40E-05
ERBB3	9.88	-1.12	1.56E-04
GFPT2	9.78	-1.81	1.72E-06
GHR	6.97	-1.38	2.74E-05
GOLM1	8.60	-1.84	4.79E-06
HK2	10.12	-1.32	5.80E-05
KCNC1	6.84	-1.21	4.94E-05
KIF3B	9.68	-1.25	3.70E-05
LRRC8B	6.49	-1.06	1.14E-04
MAD2L1	9.21	-1.03	1.26E-04
MSI2	9.99	-1.19	5.05E-05
PC	10.53	-1.19	4.41E-05
PDPR	8.45	-1.55	1.05E-05
PNPO	10.74	-1.00	4.15E-03
QDPR	8.99	-1.04	4.98E-04
RARG	10.30	-1.59	6.68E-06
SERPINE1	6.66	-1.43	3.70E-05
SLC25A15	8.77	-1.06	9.14E-05
SLC35F1	7.39	-1.48	7.75E-06
SLC39A10	8.40	-1.52	7.75E-06
SLC39A11	7.93	-1.26	3.35E-05
TERT	7.44	-1.07	3.18E-04
TPM3	12.64	-2.03	7.16E-07
TUB	6.61	-1.64	4.79E-06
UBE2E3	9.79	-1.04	1.60E-04
UBXN2B	7.60	-1.06	8.51E-05
VASH1	10.23	-1.74	4.79E-06
WWC3	9.75	-1.04	1.12E-04

miR-145-5p predicted targets			
Symbol	Av.Exp	LogFC	Adj.pVal
ABCE1	9.99	-1.25	1.09E-04
ADAM19	10.63	-1.52	1.07E-05
ADD3	9.14	-1.87	6.14E-07
ADPGK	9.60	-1.73	1.37E-06
AKAP12	7.28	-1.64	3.69E-06
AKAP9	10.57	-1.03	1.06E-04
APH1A	8.43	-1.17	5.44E-05
ARF6	7.76	-1.82	1.57E-06
ASAP2	10.19	-1.23	2.71E-05
BIRC2	10.41	-1.06	6.93E-05
BLOC1S2	11.06	-1.10	6.93E-05
BRCC3	10.32	-1.00	8.85E-04
C5ORF15	11.31	-2.00	5.42E-07
CABLES1	8.01	-1.11	6.54E-05
CBFB	10.17	-2.24	1.98E-07
CREB5	8.62	-1.13	9.63E-05
DPYSL2	9.81	-1.28	1.68E-05
EIF4EBP2	8.75	-1.43	5.75E-06
ERBB3	9.88	-1.41	1.74E-05
ETNK1	9.13	-1.09	4.90E-05
EXOC2	6.74	-1.01	1.51E-04
FAM174B	8.86	-1.69	1.40E-06
FMNL2	9.74	-1.57	8.25E-06
FSCN1	13.75	-3.24	3.35E-08
G3BP1	12.80	-2.19	4.63E-07
GABARAPL2	9.73	-1.93	1.10E-06
GALNT1	8.59	-1.34	1.28E-05
GMFB	8.71	-1.82	1.22E-06
GRB10	11.17	-1.39	6.25E-05
HLTF	9.16	-1.26	1.77E-05
HMGB3	7.94	-2.13	3.61E-07
HS6ST1	8.38	-1.29	1.78E-05
ITGB8	9.52	-1.63	1.82E-06
ITPRIPL2	11.56	-1.20	2.81E-05
IVNS1ABP	10.64	-1.43	6.30E-06
KIAA0319L	8.36	-1.03	8.13E-05
KIFAP3	12.32	-1.04	1.07E-04
LHFPL2	11.21	-1.04	8.13E-05
LMNB2	7.96	-1.11	4.69E-05
MAP3K1	12.26	-1.18	2.77E-05
MAP3K11	12.01	-1.13	4.56E-04
MBD2	10.53	-1.11	5.48E-05

MDFIC	7.89	-1.73	2.26E-06
MED13	10.11	-1.25	2.32E-05
MGAT4B	11.98	-1.69	2.59E-06
MOCS2	8.77	-1.12	8.38E-05
MPP5	9.88	-1.18	3.85E-05
MRPL17	11.19	-1.50	3.69E-06
MYO5A	9.72	-2.07	2.49E-06
NAP1L1	11.37	-1.50	6.77E-06
NDFIP2	10.04	-1.44	5.23E-06
NDUFA4	15.07	-1.88	8.46E-07
NFIB	8.57	-1.08	9.54E-05
NUAK1	8.39	-1.22	2.43E-05
NUDT3	8.25	-1.29	1.55E-05
OTOR	14.05	-1.60	2.40E-06
PAFAH1B2	10.81	-1.63	2.45E-06
PCBP2	7.63	-1.82	1.22E-06
PDCD4	8.79	-1.77	1.40E-06
PODXL	9.07	-1.12	8.56E-05
PPIP5K2	8.19	-1.07	6.75E-05
PPP3CA	8.43	-1.22	3.00E-05
PPP6C	6.49	-1.01	1.38E-04
PRKD3	9.26	-1.82	7.71E-07
RIN2	8.53	-1.38	1.12E-05
RNF170	7.87	-1.54	3.82E-06
SAP30L	7.20	-1.10	4.57E-05
SERINC5	6.96	-1.47	4.83E-05
SERPINE1	6.66	-2.09	1.16E-06
SIRPA	7.15	-1.69	1.64E-06
SLC26A2	13.07	-2.69	5.27E-08
SLC35B3	7.46	-1.35	1.77E-05
SLC7A8	8.83	-1.21	1.89E-04
SNTB2	10.06	-1.20	4.68E-05
SOX11	10.83	-1.20	4.01E-05
ST3GAL6	8.87	-1.11	4.71E-05
STC1	7.97	-1.08	8.01E-05
SWAP70	8.11	-2.48	8.21E-08
TAGLN2	10.49	-1.15	6.26E-05
TGFBR2	11.53	-2.13	3.61E-07
TPM3	12.64	-1.65	1.72E-06
TRIM2	8.90	-2.47	8.21E-08
TRIM44	10.01	-1.16	8.07E-05
TSPAN6	8.93	-1.39	7.83E-06
TULP4	12.85	-1.04	7.27E-05
UBXN4	8.00	-1.03	1.35E-04
UHMK1	10.08	-1.97	7.41E-07
USP13	8.44	-1.02	3.89E-04
UXS1	10.78	-1.30	1.56E-05
XRN1	8.28	-1.08	1.00E-04
YES1	8.72	-1.07	1.07E-04
ZBTB33	8.15	-1.14	4.07E-05
ZNF395	10.43	-1.45	2.81E-05

Appendix Table S3. List of the main predicted targets for miR-143-3p and miR-145-5p significantly downregulated following stable overexpression of the miR-143/145 cluster in human M238P melanoma cells using the bioinformatics tool miRonTop.

M238P melanoma cells were transduced with a lentivirus containing the sequence of the miR-143/-145 cluster or a control vector. Expression profiles were determined with RNA-seq (Dataset 2). Predicted targets were obtained using the bioinformatics tool miRonTop (https://www.genomique.info:8443/merge/index) (Le Brigand et al., 2010) based on the Targetscan database (conserved sites) (www.targetscan.org) (Agarwal et al., 2015) . Av.Exp: logarithm (base 2) of the base mean ; logFC: logarithm (base 2) of the ratio of miR-143/145 vs miR-Neg; Adj.pVal: Benjamini-Hochberg adjusted pValue. The thresholds used for the analysis are: Av.Exp >6, LogFC<-1 and adj.pVal <0.05.

miR-143-3p predicted targets			
**Symbol**	**Av.Exp**	**LogFC**	**Adj.pVal**
ABHD14A	7.25	-1.14	6.76E-05
ADD3	10.82	-2.09	4.98E-30
ARHGEF1	11.64	-1.82	1.81E-16
ARID3B	7.13	-1.37	7.38E-04
ASAP3	7.48	-2.16	1.00E-14
ATP10A	8.13	-2.71	1.28E-23
C6ORF62	12.36	-1.57	1.26E-11
CACHD1	8.72	-1.04	2.04E-05
CBFB	11.14	-1.21	1.62E-10
CBX5	11.96	-1.31	9.33E-12
CCDC58	9.61	-1.14	1.59E-11
CDK1	11.27	-6.95	7.39E-63
CHEK2	9.20	-1.42	9.90E-08
CREBL2	10.44	-1.87	3.55E-35
CRELD1	9.77	-1.33	4.44E-13
DLK1	11.42	-1.82	4.37E-20
EFS	8.79	-4.13	1.86E-15
ENPP1	7.91	-1.06	1.13E-03
EPM2AIP1	10.44	-1.28	6.68E-15
ERBB3	11.63	-1.80	1.48E-10
FSCN1	12.78	-4.76	5.76E-117
GOLM1	11.67	-1.01	5.90E-08
GPR124	10.05	-1.01	2.90E-04
GXYLT1	9.35	-2.11	5.74E-18
HRK	6.05	-1.30	1.85E-02
KCNC1	7.55	-1.13	3.12E-02
MAD2L1	10.49	-5.17	1.12E-45
MLLT3	8.38	-1.23	4.52E-05
MRC2	13.56	-1.24	2.51E-20
MYBL2	10.77	-5.37	1.21E-56
NIPSNAP1	11.31	-1.74	3.66E-33
PDGFRA	9.39	-1.60	9.54E-17
PGK1	14.62	-1.51	2.97E-11
POC1A	8.81	-2.07	9.44E-07
QDPR	10.05	-1.79	1.57E-27
RARG	10.58	-1.68	3.60E-16
SERPINE1	11.06	-1.86	4.90E-08
SLC25A15	9.46	-1.01	1.69E-07
SMARCD2	11.49	-1.49	1.55E-19
SOBP	8.58	-1.05	1.95E-03
STOX2	7.49	-1.39	3.71E-04

miR-145-5p predicted targets			
**Symbol**	**Av.Exp**	**LogFC**	**Adj.pVal**
ADAM19	10.14	-1.89	4.41E-06
ADD3	10.82	-2.09	4.98E-30
ANGEL2	10.17	-1.20	2.09E-14
APH1A	11.72	-1.51	3.50E-11
ARHGAP6	9.18	-1.54	5.81E-16
ARL5B	10.12	-1.04	4.18E-03
BIRC2	11.56	-1.01	6.51E-03
BRI3BP	10.47	-1.33	1.31E-06
CACHD1	8.72	-1.04	2.04E-05
CBFB	11.14	-1.21	1.62E-10
CCDC25	10.50	-1.14	2.07E-13
CDCA3	9.95	-5.20	4.22E-10
CIRBP	12.27	-1.08	3.68E-08
CTNNBIP1	10.23	-1.52	3.10E-07
DUT	10.91	-1.70	7.63E-15
EBF3	10.22	-1.48	7.80E-24
EFNA3	6.54	-1.12	1.93E-02
EFNB3	7.73	-1.35	7.93E-07
ERBB3	11.63	-1.80	1.48E-10
ERMP1	9.53	-1.22	6.96E-06
ESCO2	8.87	-4.77	2.89E-42
FANCA	9.74	-3.04	4.08E-18
FSCN1	12.78	-4.76	5.76E-117
GABARAPL2	10.90	-1.13	7.04E-11
GGT7	10.85	-1.17	2.88E-14
GINS2	9.33	-3.03	1.66E-26
GJA5	6.41	-1.48	3.18E-03
GMFB	10.50	-1.75	3.72E-12
GOPC	10.81	-1.01	1.37E-10
GPRC5A	12.72	-1.54	1.85E-09
GXYLT1	9.35	-2.11	5.74E-18
H2AFX	11.41	-3.11	7.87E-16
HELLS	9.70	-2.23	3.78E-24
HHEX	8.15	-3.93	9.24E-09
HMGB3	10.48	-1.52	5.46E-06
HOMER2	8.19	-1.04	5.66E-06
KCNMB4	6.38	-1.32	8.59E-04
KIAA1586	8.57	-1.25	1.60E-07
LDLRAD3	10.33	-1.48	6.52E-16
LMNB2	12.26	-1.95	1.32E-09
MAP2K6	6.84	-1.81	3.16E-06

TERT	6.91	-4.77	2.12E-15
TIGD5	8.57	-1.78	3.22E-07
TMEM134	9.10	-1.02	1.36E-05
TNFRSF11A	6.19	-1.42	5.56E-04
TPM3	13.36	-2.41	1.19E-67
TUB	8.43	-3.22	2.37E-32
UCK2	11.33	-1.22	5.63E-15
VASH1	9.32	-1.10	8.57E-07
VWA1	10.50	-1.30	5.68E-11
YIF1B	9.88	-2.92	4.96E-25
PHF6	10.39	-1.88	5.45E-23

MAP3K11	11.00	-1.48	1.71E-09
MDFIC	10.26	-1.42	1.48E-07
MEST	6.30	-2.13	1.36E-02
MGAT4B	12.19	-1.01	2.66E-11
MRPL17	10.93	-1.13	2.99E-14
NET1	10.54	-1.69	3.35E-26
NFIA	7.40	-1.18	4.77E-05
NINL	6.07	-1.30	8.01E-03
NUCKS1	13.57	-1.42	3.35E-11
NUDT3	10.73	-1.49	3.70E-24
OSBPL1A	10.04	-1.10	7.70E-09
P4HA1	12.29	-1.09	5.23E-11
PAN2	10.47	-1.19	1.33E-10
PCYT1B	7.18	-1.49	2.16E-02
PDCD4	11.46	-1.09	3.03E-16
PHB2	13.07	-1.12	3.78E-09
PI4K2B	9.26	-1.17	2.27E-08
PRKD3	12.05	-1.40	1.57E-27
PXN	13.96	-1.03	7.17E-08
RAD18	10.23	-1.29	1.18E-16
RNF170	8.88	-1.57	1.30E-09
RPA1	12.02	-1.06	6.40E-09
SCARB1	11.49	-2.17	1.46E-23
SERPINE1	11.06	-1.86	4.90E-08
SH3BP1	9.11	-1.35	2.43E-09
SIRPA	7.96	-3.37	2.27E-28
SLC25A36	11.80	-1.03	7.87E-07
SLC26A2	13.29	-1.34	3.31E-07
SLC7A8	10.72	-1.70	1.04E-09
SNTB2	11.57	-1.22	2.64E-06
SPTLC2	10.40	-1.00	3.04E-09
ST6GALNAC3	7.01	-2.34	2.17E-12
SWAP70	10.23	-1.41	5.23E-18
TBC1D14	11.97	-1.58	3.82E-29
TOMM40	11.53	-1.01	5.41E-06
TPM3	13.36	-2.41	1.19E-67
UBE2W	9.58	-1.17	8.90E-04
UBTF	11.92	-1.07	8.03E-08
USP13	10.13	-1.35	7.70E-09
UXS1	11.65	-1.00	1.44E-03
VASN	6.38	-1.92	7.67E-07
ZBTB8A	6.47	-1.30	1.37E-03
ZNF395	9.84	-1.46	4.73E-11

Appendix Table S4. Human primers sequences used in the study.

Gene	Forward	Reverse
ACTA2	CTGTTCCAGCCATCCTTCAT	TCATGATGCTGTTGTAGGTGGT
AMOTL2	GCGACTGTCAGAACAACTGC	GCACCTTTAACCTGCTTTCCA
AXL	GTGGGCAACCCAGGGAATATC	GTACTGTCCCGTGTCGGAAAG
COL1A1	GGGATTCCCTGGACCTAAAG	GGAACACCTCGCTCTCCA
COL1A2	GTTGCTGCTTGCAGTAACCTT	AGGGCCAAGTCCAACTCCTT
CTGF	ACCGACTGGAAGACACGTTTG	CCAGGTCAGCTTCGCAAGG
CYR61	TGAAGCGGCTCCCTGTTTT	CGGGTTTCTTTCACAAGGCG
FN1	TGTTATGGAGGAAGCCGAGGTT	GCAGCGGTTTGCGATGGT
FSCN1	CCAGCTGCTACTTTGACATCGA	GCTCTGAGTCCCCTGCTGTCT
GDNF	GGCAGTGCTTCCTAGAAGAGA	AAGACACAACCCCGGTTTTTG
LOX	CGACCCTTACAACCCCTACA	AAGTAGCCAGTGCCGTATCC
LOXL2	CCTGGGGAGAGGACATACAA	CTCGCAGGTGACATTCTTCA
MYL9	CATCCATGAGGACCACCTCCG	CTGGGGTGGCCTAGTCGTC
RPL32	CCTTGTGAAGCCCAAGATCG	TGCCGGATGAACTTCTTGGT
TAGLN2	ATGGCACGGTGCTATGTGAG	CCCACCCAGATTCATCAGCG
THBS1	AGACTCCGCATCGCAAAGG	TCACCACGTTGTTGTCAAGGG
TNC	TCCCAGTGTTCGGTGGATCT	TTGATGCGATGTGTGAAGACA

Appendix Table S5. Mouse primers sequences used in the study.

Gene	Forward	Reverse
Acta2	CCCAGACATCAGGGAGTAATGG	TCTATCGGATACTTCAGCGTCA
Amotl2	AGGGACAATGAGCGATTGCAG	CCTCACGCTTGGAAGAGGT
Col1a1	GCTCCTCTTAGGGGCCACT	ATTGGGGACCCTTAGGCCAT
Ctgf	GGCCTCTTCTGCGATTTCG	GCAGCTTGACCCTTCTCGG
Cyr61	TAAGGTCTGCGCTAAACAACTC	CAGATCCCTTTCAGAGCGGT
Fn1	ATGTGGACCCCTCCTGATAGT	GCCCAGTGATTTCAGCAAAGG
Myl9	AGAGGGCTACGTCCAATGTCT	CTCCAGATACTCGTCTGTGGG
Rpl32	AAAAACAGACGCACCATCGAA	TTCAGGTGACCACATTCAGGG
Tagln2	GCTATGGCATTAACACCACGG	CCCAGGTTCATTAGTGTCCGC
Tnc	TTTGCCCTCACTCCCGAAG	AGGGTCATGTTTAGCCCACTC

Appendix Table S6. List of antibodies used in the study.

Primary Antibody	Company	Catalog Number	Dilution
p-AKT (Ser473)	Cell Signaling	9271	WB 1:1000
AKT (pan)	Cell Signaling	4691	WB 1:1000
αSMA	Abcam	ab5694	WB 1:1000
AXL	Cell Signaling	4566	WB 1:1000
CDK2	Santa Cruz	sc-6248	WB 1:500
COL1	Abcam	ab34710	WB 1:3000
CCND1	BD Biosciences	556470	WB 1:1000
E2F1	Cell Signaling	3742	WB 1:1000
EGFR	Santa Cruz	sc-373746	WB 1:500
p-ERK1/2 (Thr202/Tyr204)	Cell Signaling	9101	WB 1:1000
ERK2	Santa Cruz	sc-1647	WB 1:500
FAK	Upstate	05-182	WB 1:1000
p-FAK (Tyr397)	Cell Signaling	3283	WB 1:500
FN1	Santa Cruz	sc-8422	WB 1:500, IF 1:100
FSCN1	Santa Cruz	sc-21743	IF 1:100
FSCN1	Proteintech	66321-1-Ig	WB 1:1000
HSP60	Santa Cruz	sc-57840	WB 1:500
HSP90	Santa Cruz	sc-13119	WB 1:500
LOX	Novus Biologicals	NB100-2527SS	WB 1:1000
LOXL2	R&D Systems	AF2639	WB 1:1000
MITF	Sigma	HPA003259	WB 1:1000
MLC2	Cell Signaling	3672	WB 1:1000
p-MLC2 (Thr18/Ser19)	Cell Signaling	3674	WB 1:500
MRTFA	Santa Cruz	sc-390324	IF 1:100
NGFR (p75NTR)	Cell Signaling	8238	WB 1:1000
p27 Kip1	Cell Signaling	3686	WB 1:1000
Paxillin	BD Biosciences	P13520	WB 1:3000
p-Paxillin (Tyr118)	Cell Signaling	2541	WB 1:1000 IF 1:50
p-PDGFRβ (Tyr1009)	Cell Signaling	3124	WB 1:500
PDGFRβ	Santa Cruz	sc-374573	WB 1:500
p-Rb	Cell Signaling	9308	WB 1:1000
Rb	Cell Signaling	9309	WB 1:1000
p-SMAD3 (Ser433/435)/SMAD1 (Ser463/465)	Cell Signaling	9514	WB 1:1000
SMAD1/2/3	Santa Cruz	sc-7960	WB 1:500
SOX9	Santa Cruz	sc-166505	WB 1:500
SOX10	Cell Signaling	89356	WB 1:1000
pSrc family (Tyr416)	Cell Signaling	6943	WB 1:1000
Src	Cell Signaling	2109	WB 1:1000
STAT3	Cell Signaling	9139	WB 1:1000
p-STAT3 (Tyr705)	Cell Signaling	9145	WB 1:1000
Survivin	Cell Signaling	2808	WB 1:1000
TAGLN2	Genetex	GTX115082	WB 1:1000
THBS1	Santa Cruz	sc-393504	WB 1:500
TNC	R&D Systems	AF3358	WB 1:1000
YAP	Cell Signaling	14074	IF 1:200

**Secondary Antibody**	**Company**	**Catalog Number**	**Dilutions**
Anti-mouse IgG, HRP-linked antibody	Cell Signaling	7076	WB 1:2000
Anti-rabbit IgG, HRP-linked antibody	Cell Signaling	7074	WB 1:2000
Anti-goat IgG, HRP-linked antibody	Santa Cruz	sc-2354	WB 1:5000
Goat- anti-mouse, Alexa Fluor® 488	Invitrogen	A11001	IF 1:200
Goat anti-mouse, Alexa Fluor® 594	Invitrogen	A11005	IF 1:200
Goat anti-rabbit, Alexa Fluor® 594	Invitrogen	A11012	IF 1:200

Appendix Table S7. Exact P Values obtained for the statistical analyses provided in the main figures.

Figure 1B:

BRAFi/MEKi vs BRAFi/MEKi + BIBF	0.0023

Figure 1C:

BRAFi/MEKi vs BRAFi/MEKi + BIBF	<0.0001

Figure 1F:

BRAFi/MEKi vs Ctrl	<0.0001
BRAFi/MEKi + BIBF vs Ctrl	0.0001
BRAFi/MEKi + BIBF vs BRAFi/MEKi	0.0015

Figure 1J:

BRAFi/MEKi vs Ctrl	<0.0001
BIBF vs Ctrl	<0.0001
BRAFi/MEKi + BIBF vs Ctrl	<0.0001
BRAFi/MEKi + BIBF vs BRAFi/MEKi	<0.0001

Figure 2B:

miR-143 fold change expression BIBF vs Ctrl	0.6855
miR-143 fold change expression BRAFi/MEKi vs Ctrl	0.0026
miR-143 fold change expression BRAFi/MEKi+ BIBF vs Ctrl	0.9995
miR-143 fold change expression BRAFi/MEKi+ BIBF vs BRAFi/MEKi	0.0029
miR-145 fold change expression BIBF vs Ctrl	0.7698
miR-145 fold change expression BRAFi/MEKi vs Ctrl	0.0003
miR-145 fold change expression BRAFi/MEKi+ BIBF vs Ctrl	0.9651
miR-145 fold change expression BRAFi/MEKi+ BIBF vs BRAFi/MEKi	0.0005

Figure 2C:

miR-143 fold change expression BIBF vs Ctrl	0.7423
miR-143 fold change expression BRAFi/MEKi vs Ctrl	<0.0001
miR-143 fold change expression BRAFi/MEKi+ BIBF vs Ctrl	0.0237
miR-143 fold change expression BRAFi/MEKi+ BIBF vs BRAFi/MEKi	0.2027
miR-145 fold change expression BIBF vs Ctrl	0.9601
miR-145 fold change expression BRAFi/MEKi vs Ctrl	<0.0001
miR-145 fold change expression BRAFi/MEKi+ BIBF vs Ctrl	0.9997
miR-145 fold change expression BRAFi/MEKi+ BIBF vs BRAFi/MEKi	<0.0001

Figure 2D:

miR-143 fold change expression TGFβ vs Ctrl	0.0344
miR-143 fold change expression PDGF-BB vs Ctrl	0.0006
miR-145 fold change expression TGFβ vs Ctrl	0.0006
miR-145 fold change expression PDGF-BB vs Ctrl	0.0305

Figure 2E:

miR-143 fold change expression SB vs Ctrl	0.0089
miR-143 fold change expression BIBF vs Ctrl	0.0177
miR-143 fold change expression GSK vs Ctrl	0.0004
miR-145 fold change expression SB vs Ctrl	0.0129
miR-145 fold change expression BIBF vs Ctrl	0.0138
miR-145 fold change expression GSK vs Ctrl	0.028

Figure 3B:

MYL9 BRAFi LNA-143 vs BRAFi	0.0245
MYL9 BRAFi LNA-145 vs BRAFi	0.0007
MYL9 BRAFi LNA-143/145 vs BRAFi	0.0013
FN1 BRAFi LNA-143 vs BRAFi	0.0168
FN1 BRAFi LNA-145 vs BRAFi	0.0035
FN1 BRAFi LNA-143/145 vs BRAFi	0.0075
ACTA2 BRAFi LNA-143 vs BRAFi	0.0038
ACTA2 BRAFi LNA-145 vs BRAFi	0.0004
ACTA2 BRAFi LNA-143/145 vs BRAFi	0.0009
COL1A1 BRAFi LNA-143 vs BRAFi	0.0430
COL1A1 BRAFi LNA-145 vs BRAFi	0.0043
COL1A1 BRAFi LNA-143/145 vs BRAFi	0.0075
LOXL2 BRAFi LNA-143 vs BRAFi	0.0295
LOXL2 BRAFi LNA-145 vs BRAFi	0.0017
LOXL2 BRAFi LNA-143/145 vs BRAFi	0.0041
THBS1 BRAFi LNA-143 vs BRAFi	0.7806
THBS1 BRAFi LNA-145 vs BRAFi	0.0036
THBS1 BRAFi LNA-143/145 vs BRAFi	0.0200

Figure 3E:

miR-143-3p vs miR-neg	<0.0001
miR-145-5p vs miR-neg	<0.0001

Figure 3F:

miR-143-3p vs miR-neg	0.0001
miR-145-5p vs miR-neg	0.0153

Figure 3H:

BRAFi vs LNA Ctrl	<0.0001
BRAFi LNA-143 vs BRAFi	<0.0001
BRAFi LNA-145 vs BRAFi	<0.0001
BRAFi LNA-143/145 vs BRAFi	<0.0001

Figure 4F:

WT miR-143 vs miR-neg	0.0010
WT miR-145 vs miR-neg	<0.0001
Mut miR-143 vs miR-neg	0.5301
Mut miR-145 vs miR-neg	0.2430

Figure 4G:

miR-143 vs miR-neg	0.0041
miR-145 vs miR-neg	0.0007

Figure 5A:

FSCN1 BRAFi vs Ctrl	0.0002

BRAFi vs Ctrl miR-143-3p	<0.0001
BRAFi vs Ctrl miR-145-p	0.0003

Figure 5B:

BRAFi LNA-143 vs BRAFi	<0.0001
BRAFi LNA-145 vs BRAFi	<0.0001
BRAFi LNA-143/145 vs BRAFi	<0.0001

Figure 5D:

siFSCN1#1 vs siCTRL	0.0009
siFSCN1#2 vs siCTRL	0.0397

Figure 5F:

Lenti-FSCN1 vs Lenti-Ctrl	<0.0001

Figure 5H:

Lenti-FSCN1 vs Lenti-Ctrl 5 µM	<0.0001
Lenti-FSCN1 vs Lenti-Ctrl 10 µM	<0.0001
Lenti-FSCN1 vs Lenti-Ctrl 20 µM	<0.0001
Lenti-FSCN1 vs Lenti-Ctrl 30 µM	<0.0001

Figure 6A:

miR-143-3p vs miR-neg	<0.0001
miR-145-5p vs miR-neg	<0.0001

Figure 6B:

miR-143-3p vs miR-neg	<0.0001
miR-145-5p vs miR-neg	<0.0001

Figure 6C:

miR-143-3p vs miR-neg	0.0393
miR-145-5p vs miR-neg	<0.0001

Figure 6D:

miR-143-3p vs miR-neg	<0.0001
miR-145-5p vs miR-neg	0.0002

Figure 6E:

miR-143-3p vs miR-neg	<0.0001
miR-145-5p vs miR-neg	<0.0001

Figure 6F:

miR-143-3p vs miR-neg	<0.0001
miR-145-5p vs miR-neg	<0.0001

Figure 7A:

miR-143-3p vs miR-neg	<0.0001
miR-145-5p vs miR-neg	<0.0001

Figure 7B:

BRAFi vs Ctrl	<0.0001
BRAFi/MEKi vs Ctrl	<0.0001

Figure 7C:

miR-143-3p vs miR-neg	<0.0001
miR-145-5p vs miR-neg	<0.0001

Figure 7D:

BRAFi vs Ctrl	0.0002
BRAFi/MEKi vs Ctrl	<0.0001

Figure 7E:

CYR61 miR-143-3p vs miR-neg	<0.0001
CYR61 miR-145-5p vs miR-neg	0.0034
CTGF miR-143-3p vs miR-neg	0.0043
CTGF miR-145-5p vs miR-neg	0.0378
AMOTL2 miR-143-3p vs miR-neg	0.0218
AMOTL2 miR-145-5p vs miR-neg	0.0180
AXL miR-143-3p vs miR-neg	0.0158
AXL miR-145-5p vs miR-neg	0.0013

Figure 7F:

siFSCN1#1 vs siCtrl	<0.0001
siFSCN1#2 vs siCtrl	<0.0001

Figure 7G:

siFSCN1#1 vs siCtrl	0.0004
siFSCN1#2 vs siCtrl	<0.0001

Figure 7H:

CYR61 siFSCN1#1 vs siCtrl	0.0012
CYR61 siFSCN1#2 vs siCtrl	0.0013
CTGF siFSCN1#1 vs siCtrl	0.0048
CTGF siFSCN1#2 vs siCtrl	0.0023
AMOTL2 siFSCN1#1 vs siCtrl	0.0128
AMOTL2 siFSCN1#2 vs siCtrl	0.0039
THBS1 siFSCN1#1 vs siCtrl	0.0007
THBS1 siFSCN1#2 vs siCtrl	0.0453

References:

Agarwal V, Bell GW, Nam JW, Bartel DP. Predicting effective microRNA target sites in mammalian mRNAs. Elife. 2015;4.

Le Brigand K, Robbe-Sermesant K, Mari B, Barbry P. MiRonTop: mining microRNAs targets across large scale gene expression studies. Bioinformatics. 2010;26(24):3131-2.
