# Supplementary material for: Blockade of the pro‐fibrotic reaction mediated by the miR‐143/‐145 cluster enhances the responses to targeted therapy in melanoma
Source: EMBO Mol Med. 2022 Feb 14;14(3):e15295. doi: 10.15252/emmm.202115295 (PMC8899916; doi:10.15252/emmm.202115295)
Supplement: Supplementary file 2 — Expanded View Figures PDF [file EMMM-14-e15295-s005.pdf]

Expanded View Figures

Figure EV1. Administration of nintedanib/BIBF1120 resensitizes melanoma cells to MAPK-targeted therapies, delays tumor relapse, and normalizes MAPKi-induced ECM remodeling and miR-143/-145 expression.

A, B YUMM1.7 cells were subcutaneously inoculated into C57BL/6 mice, and when tumors reached 100 mm^3, mice were treated with the indicated therapies. (A) Individual graphics showing tumor growth following treatment. (B) Normalized expression of myofibroblast/CAF and ECM-related genes assessed by RT-qPCR in individual tumors treated as indicated. Data are represented as median with range ($n = 5$). One-way ANOVA was used for statistical analysis. $*P \leq 0.05$, $**P \leq 0.01$, and $****P \leq 0.0001$. Significance was calculated against the control group. Statistical significance of BRAFi/MEKi vs BRAFi/MEKi + BIBF was also calculated.

C–E Human M238P cells were treated with BRAFi (vemurafenib) + MEKi (trametinib; 1 μM), BIBF1120 (2 μM) or BRAFI + MEKi (1 μM) plus BIBF (2 μM) for 72 h. (C) Heatmap showing the expression of ECM, myofibroblast/CAF markers, and phenotype switch markers by RT-qPCR ($n = 3$). (D) Crystal violet viability assay of M238P cells treated with MAPK-targeted therapies as above. Paired Student's t-test was used for statistical analysis. $****P \leq 0.0001$. Data are represented as mean ± SD from a triplicate representative of three independent experiments. (E) Western blot showing the expression of ECM, myofibroblast/CAF markers, and activation levels of signaling pathways (AKT and ERK1/2) in the different conditions.

F–H Human M238R cells were treated with BIBF1120 (2 μM) or with CP673451 (2 μM) for 72 h. (F) Western blot showing activation levels of signaling pathways (PDGFR and AKT) in the different conditions. (G) Heatmap showing the expression of ECM, myofibroblast/CAF markers, and phenotype switch markers by RT-qPCR in M238R cells treated with the indicated inhibitors ($n = 3$). (H) Crystal violet viability assay of M238R cells treated with the indicated inhibitors. Paired Student's t-test was used for statistical analysis. $****P \leq 0.0001$. Data are represented as mean ± SD from a triplicate representative of three independent experiments.

© 2022 The Authors

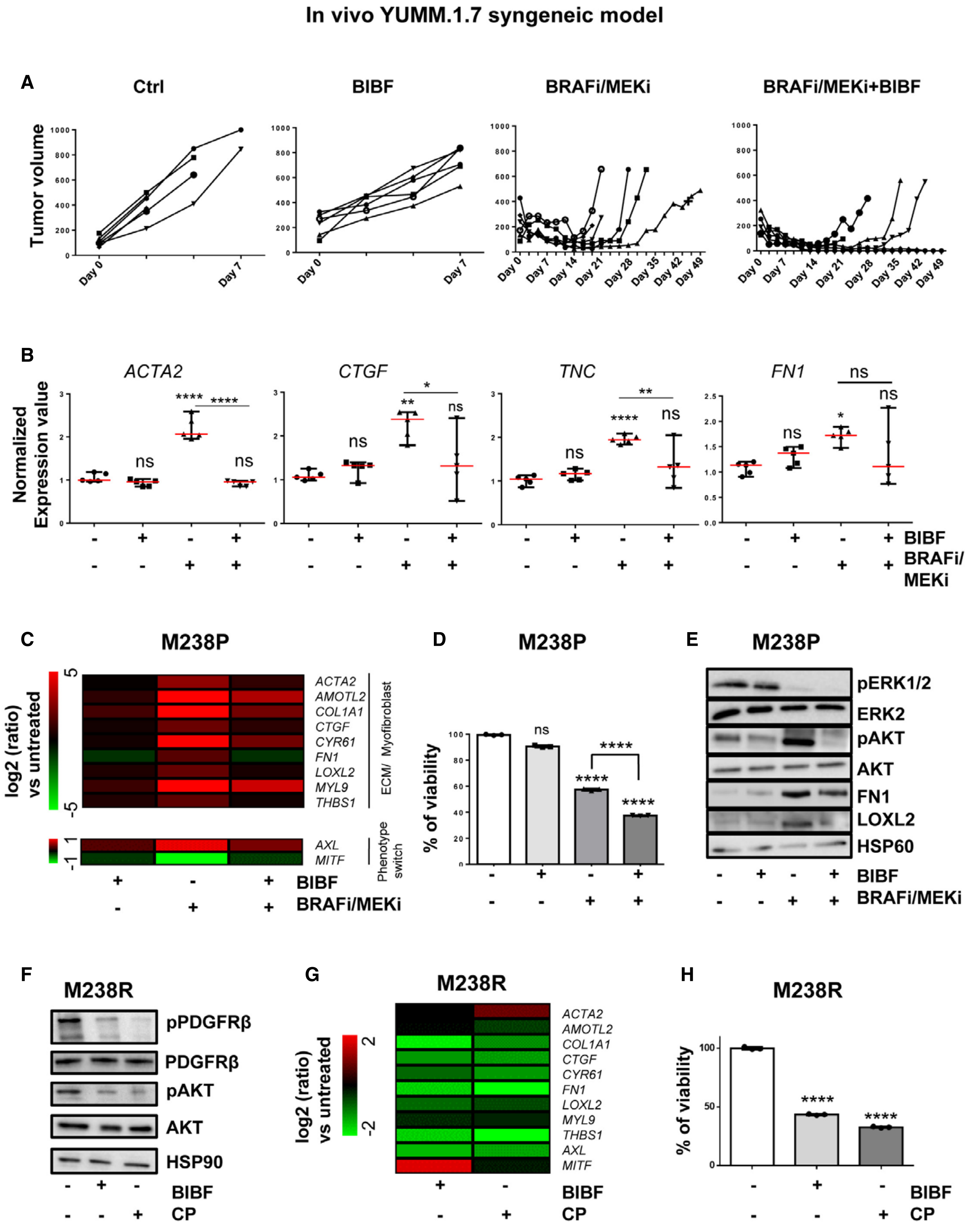

Figure EV1.

 © 2022 The Authors

Figure EV2. High expression of miR-143/-145 is correlated with an undifferentiated/mesenchymal-like BRAFi-resistant phenotype in melanoma cells.

A Relative miRNA expression levels were quantified in parental (P) and paired resistant (R) cells (M238, UACC62, M229, M249) by RT-qPCR. Log_2 (R/P) is shown for each cell line. Data are represented as mean ± SE from a triplicate representative of at least three independent experiments. Paired Student's *t*-test was used for statistical analysis. ****$P \leq 0.0001$.

B Heatmap showing the expression of ECM, myofibroblast/CAF, and phenotype switch markers, and miRNAs by RT-qPCR in A375 cells resistant to BRAFi and MEKi (cobimetinib 1 μM and trametinib 0.1 μM).

C–G Relative miRNA expression levels were quantified in human melanoma cell lines (M238P, UACC62P, 1205Lu) or short-term patient-derived cell lines (MM034, MM099) treated or not for 72 h with MAPK-targeted therapies BRAFi (vemurafenib 3 μM), MEKi (trametinib 1 μM), or BRAFi + MEKi (1 μM) by RT-qPCR and normalized to miR-16-5p.

H Relative miRNA expression levels were quantified in M238R cells treated or not for 72 h with BIBF1120 (2 μM) or CP673451 (2 μM). (C–H) Data are represented as mean ± SE from a triplicate representative of at least three independent experiments. Paired Student's *t*-test was used for statistical analysis. *$P \leq 0.05$, **$P \leq 0.01$, ***$P \leq 0.001$, and ****$P \leq 0.0001$.

© 2022 The Authors

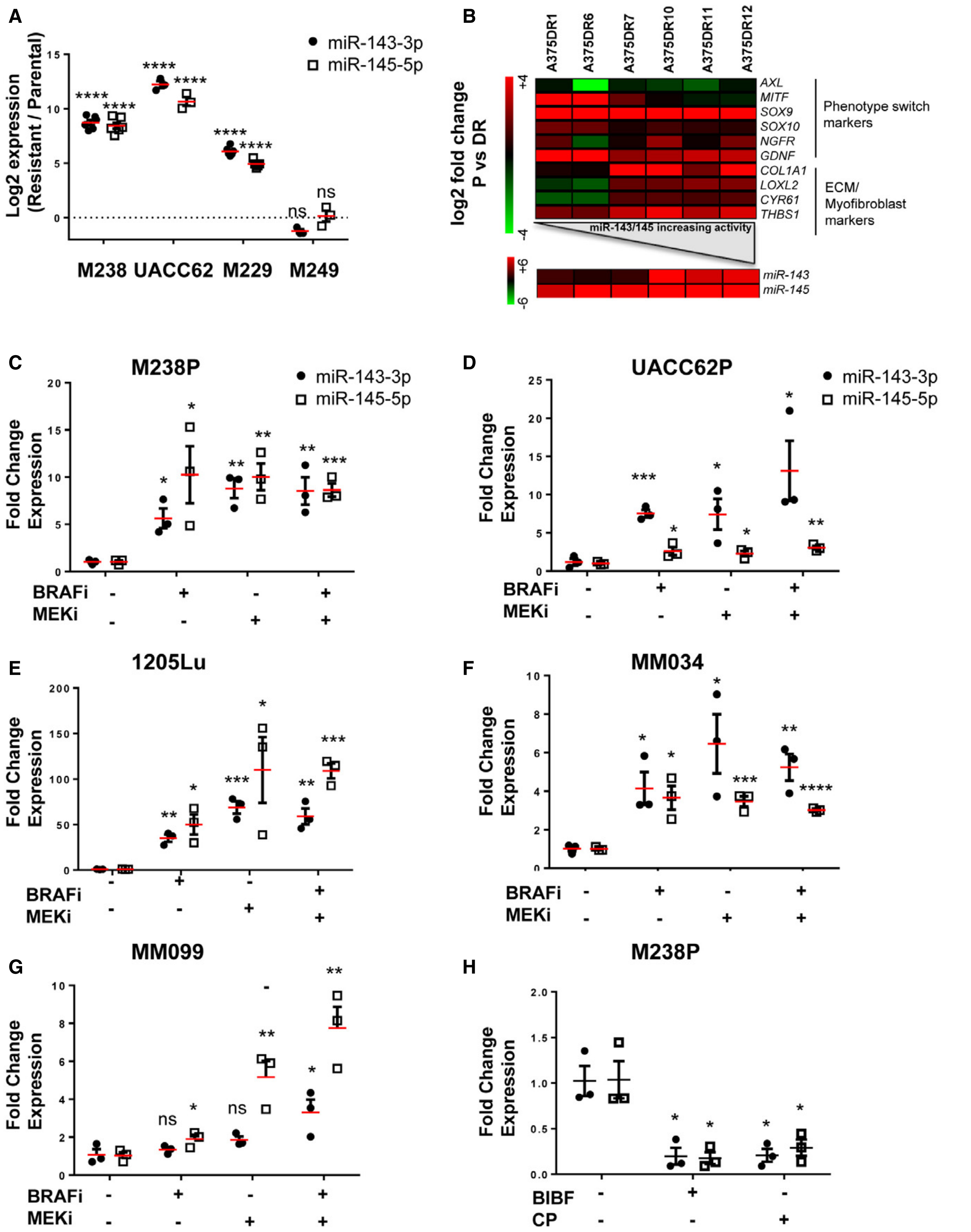
A
B
C
M238P
D
UACC62P
E
1205Lu
F
MM034
G
MM099
H
M238P
miR-143-3p
miR-145-5p
Log2 expression (Resistant / Parental)
M238 UACC62 M229 M249
log2 fold change P vs DR
A375DR1 A375DR6 A375DR7 A375DR10 A375DR11 A375DR12
AXL
MITF
SOX9
SOX10
NGFR
GDNF
COL1A1
LOXL2
CYR61
THBS1
Phenotype switch markers
ECM/ Myofibroblast markers
miR-143/145 increasing activity
miR-143
miR-145
Fold Change Expression
BRAFi
MEKi
BIBF
CP

Figure EV2.

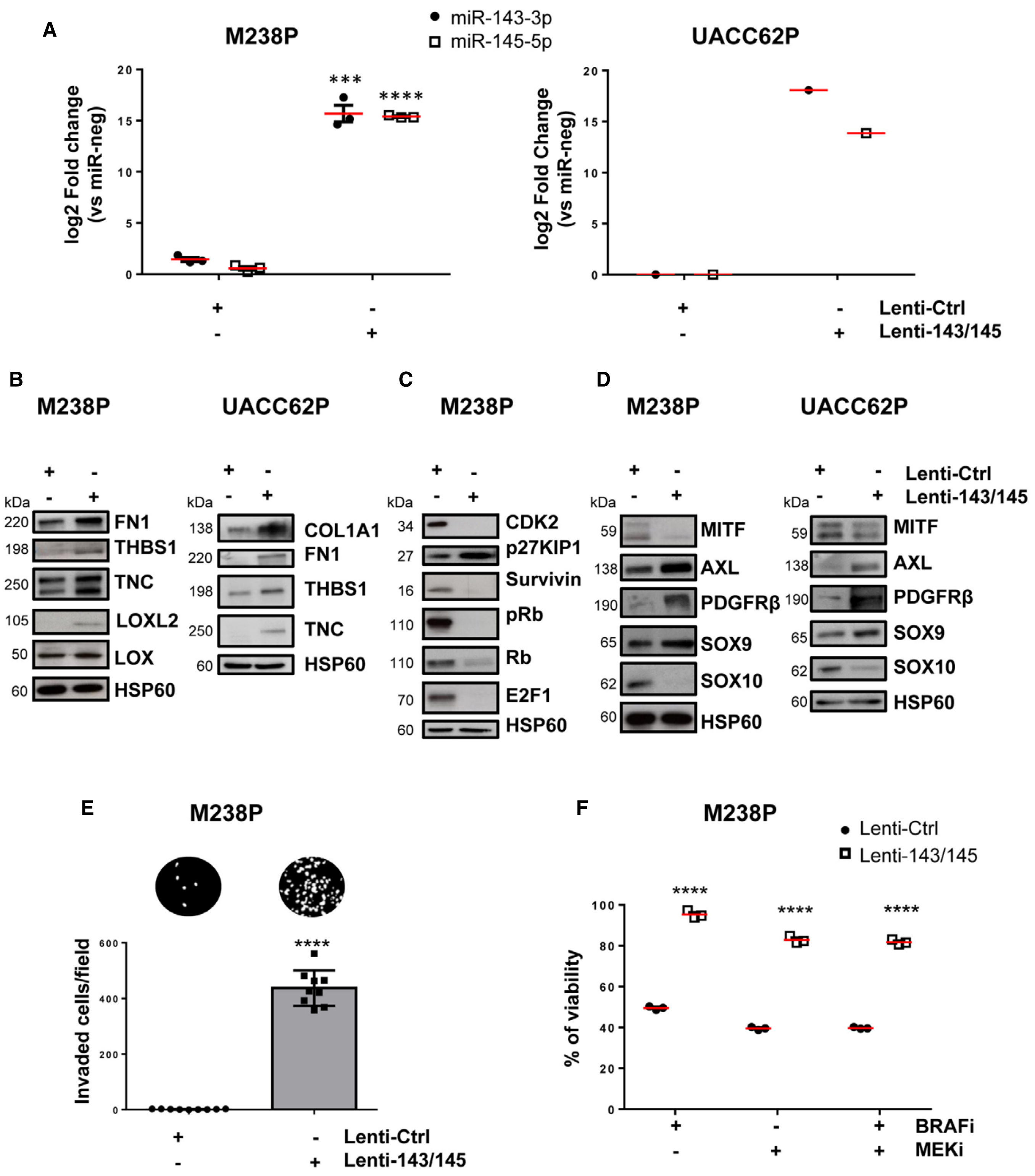
A
M238P
miR-143-3p
miR-145-5p
UACC62P
log2 Fold change (vs miR-neg)
log2 Fold Change (vs miR-neg)
Lenti-Ctrl
Lenti-143/145
B
M238P
UACC62P
kDa
FN1
THBS1
TNC
LOXL2
LOX
HSP60
COL1A1
C
CDK2
p27KIP1
Survivin
pRb
Rb
E2F1
D
MITF
AXL
PDGFRβ
SOX9
SOX10
E
Invaded cells/field
F
% of viability
BRAFi
MEKi

Figure EV3.

Figure EV3. Stable expression of the miR-143/-145 cluster promotes ECM remodeling and drives melanoma cell dedifferentiation.

A RT-qPCR analysis showing the level of miR-143-3p and miR-145-5p expression after stable expression following lentivirus transduction of two melanoma cell lines (M238P, UACC62P). Data are represented as mean ± SE from a triplicate representative of at least three independent experiments. Paired Student's *t*-test was used for statistical analysis. ***$P \leq 0.001$ and ****$P \leq 0.0001$.

B–D Western blot analysis of ECM remodeling (B), cell cycle (C), and phenotype switch markers (D) on total cell lysates from the different stable cell lines.

E Invasion assay in Boyden chambers. Representative images show invasion in control and miR-143/145 expressing cells (M238P). The bar graph represents the quantitative determination of data obtained using ImageJ software. Paired Student's *t*-test was used for statistical analysis. ****$P \leq 0.0001$.

F Viability of M238P cells transduced with a control or a miR-143/-145 cluster lentivirus was assessed by crystal violet staining upon MAPKi treatment (6 days, BRAFi, vemurafenib 3 μM, MEKi, trametinib, 3 μM or BRAFi + MEKi, 5 μM). Paired Student's *t*-test was used for statistical analysis. ****$P \leq 0.0001$.

Figure EV4. FSCN1 is a functional miR-143/-145 target contributing to the phenotypic switch toward an invasive dedifferentiated state.

A Western blot analysis of FSCN1 expression in parental and paired resistant cells (M238, UACC62, M229, M249).

B Western blot analysis of FSCN1 levels in parental cells (UACC62P) treated with BRAFi, (vemurafenib, 3 μM), MEKi (trametinib, 1 μM), or BRAFi + MEKi (1 μM) for 72 h.

C–F Cells were transfected with two different sequences of siRNAs vs FSCN1 or with a control siRNA (100 nM). (C) Western blot analysis of cell cycle markers on cell lysates from M229P cells cultured for 72 h following transfection with the different siRNAs. (D) Proliferation curves using time-lapse analysis of cells with the IncuCyte system. Graph shows quantification of cell confluence. Two-way ANOVA was used for statistical analysis. ****$P \leq 0.0001$. (E) Migration assay performed in Boyden chambers. Representative images showing migration of UACC62P cells in the indicated conditions. The bar graph represents the quantitative determination of data obtained using ImageJ software. Paired Student's *t*-test was used for statistical analysis. **$P \leq 0.01$ and ****$P \leq 0.0001$. (F) Western blot analysis of phenotype switch markers on cell lysates from cells (M229P and UACC62P) transfected with the different siRNAs.

 © 2022 The Authors

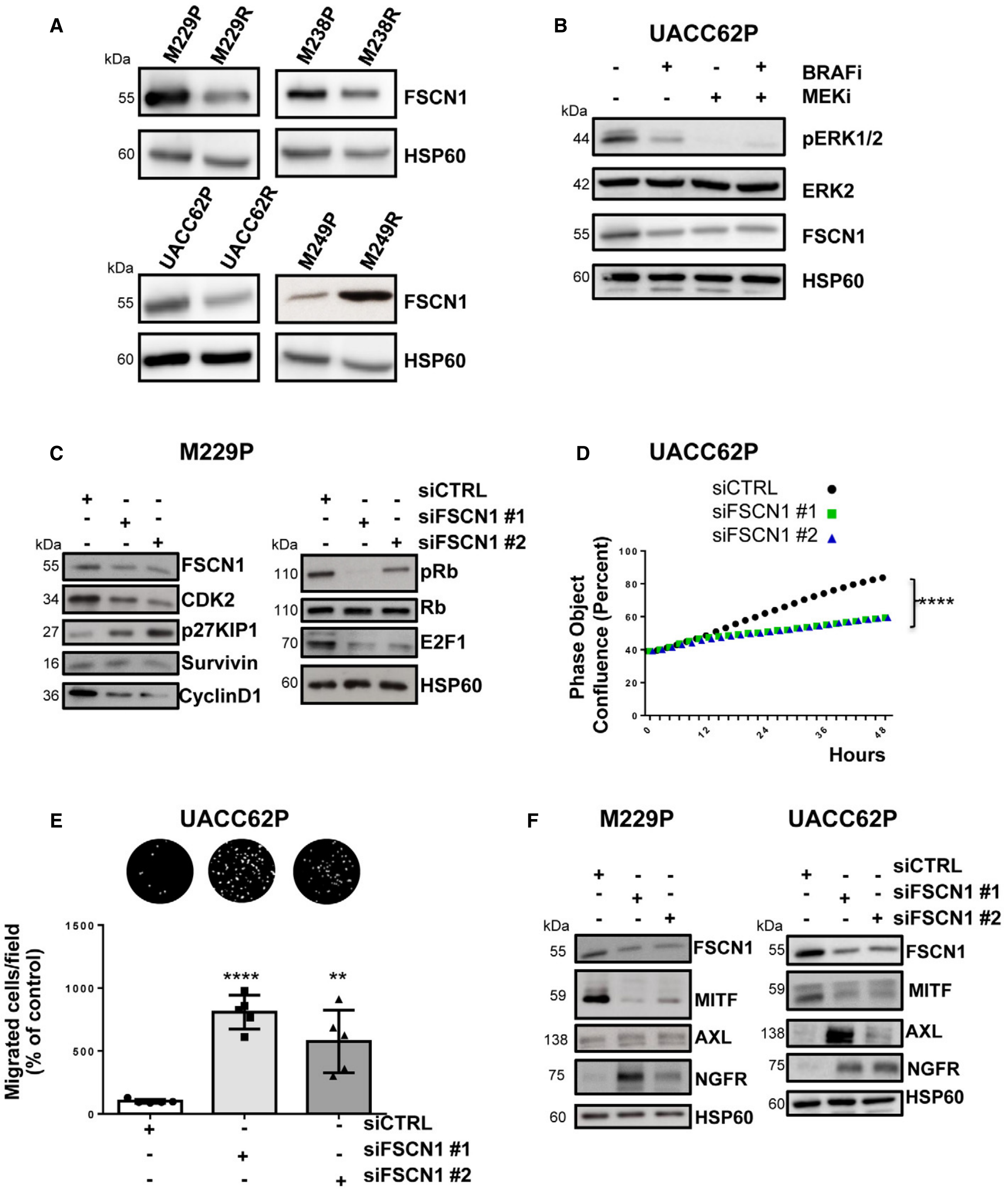
A
M229P
M229R
M238P
M238R
kDa
55
60
FSCN1
HSP60
UACC62P
UACC62R
M249P
M249R
B
UACC62P
BRAFi
MEKi
pERK1/2
ERK2
FSCN1
HSP60
C
M229P
siCTRL
siFSCN1 #1
siFSCN1 #2
FSCN1
CDK2
p27KIP1
Survivin
CyclinD1
pRb
Rb
E2F1
HSP60
D
UACC62P
Phase Object Confluence (Percent)
Hours
****
E
UACC62P
Migrated cells/field (% of control)
****
**
F
M229P
UACC62P
MITF
AXL
NGFR

Figure EV4.

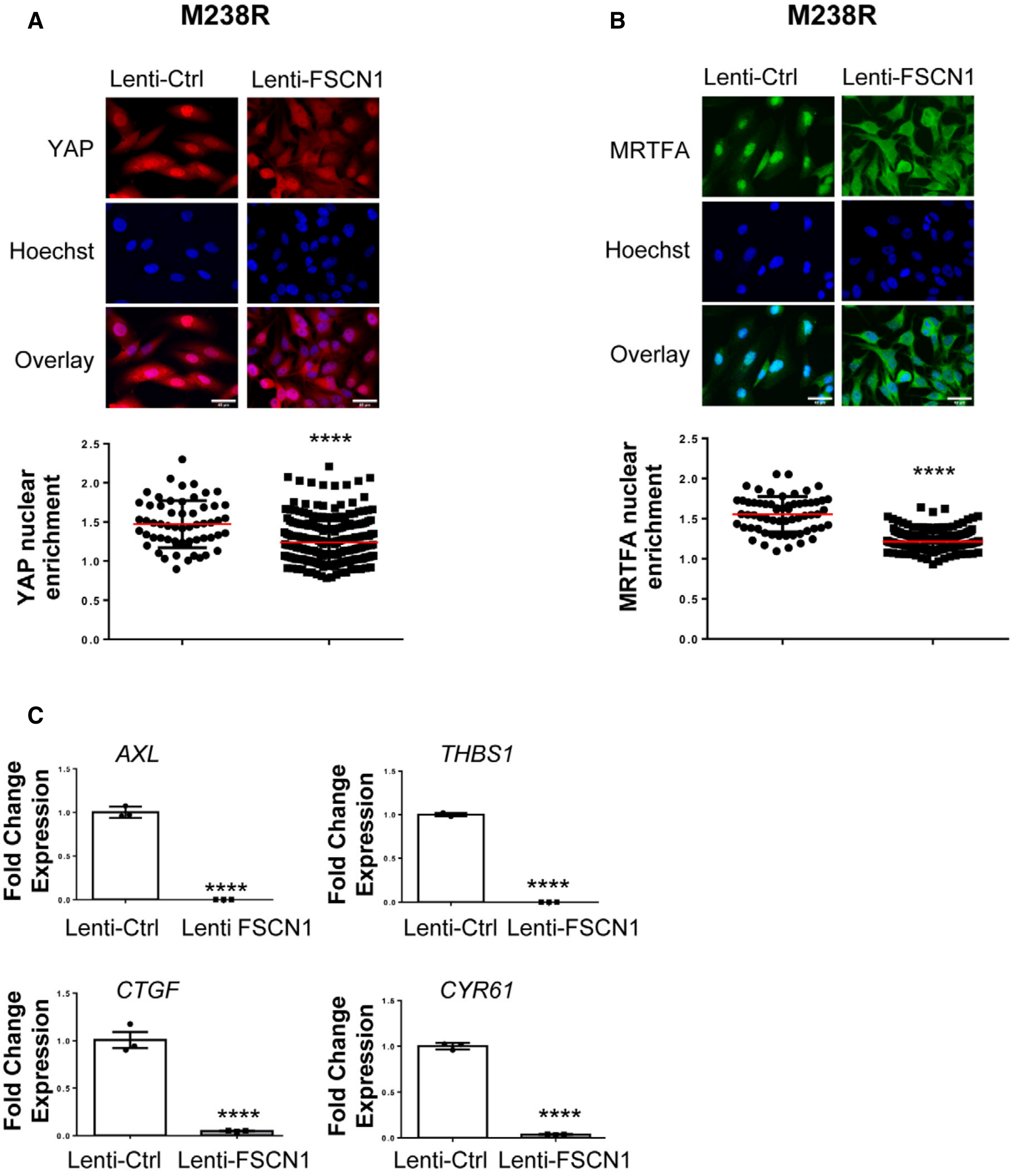

Figure EV5. FSCN1 restoration impairs the activation of mechanopathways.

BRAFi-resistant M238R cells overexpressing FSCN1 were obtained after transduction with a FSCN1 lentiviral construct. M238R cells transduced with a Ctrl lentivirus were used as control.

A, B Effect of FSCN1 overexpression on YAP (A) and MRTFA (B) nuclear translocation assessed by immunofluorescence in cells stained for YAP (red) or MRTFA (green) and nuclei (blue). Data are represented as scatter plot with mean ± SD ($n \geq 30$ cells per condition). Each point represents the nuclear/cytoplasm ratio. The Mann–Whitney *U*-test was used for statistical analysis. ****$P \leq 0.0001$. Scale bar 40 μm.

C RT-qPCR analysis for the expression of YAP1/MRTFA target genes in M238R cells stably overexpressing FSCN1. Data are normalized to the expression in parental control cells. Data are represented as mean ± SE from a triplicate representative of at least 3 independent experiments. Paired Student's *t*-test was used for statistical analysis. ****$P \leq 0.0001$.

 © 2022 The Authors
