# Supplementary material for: Blockade of the pro‐fibrotic reaction mediated by the miR‐143/‐145 cluster enhances the responses to targeted therapy in melanoma
Source: EMBO Mol Med. 2022 Feb 14;14(3):e15295. doi: 10.15252/emmm.202115295 (PMC8899916; doi:10.15252/emmm.202115295)
Supplement: Supplementary file 3 — Source Data for Figure 1 [file EMMM-14-e15295-s006.zip › emmm-202115295-sup-0002-SDataFig1.pptx]

## Slide 1
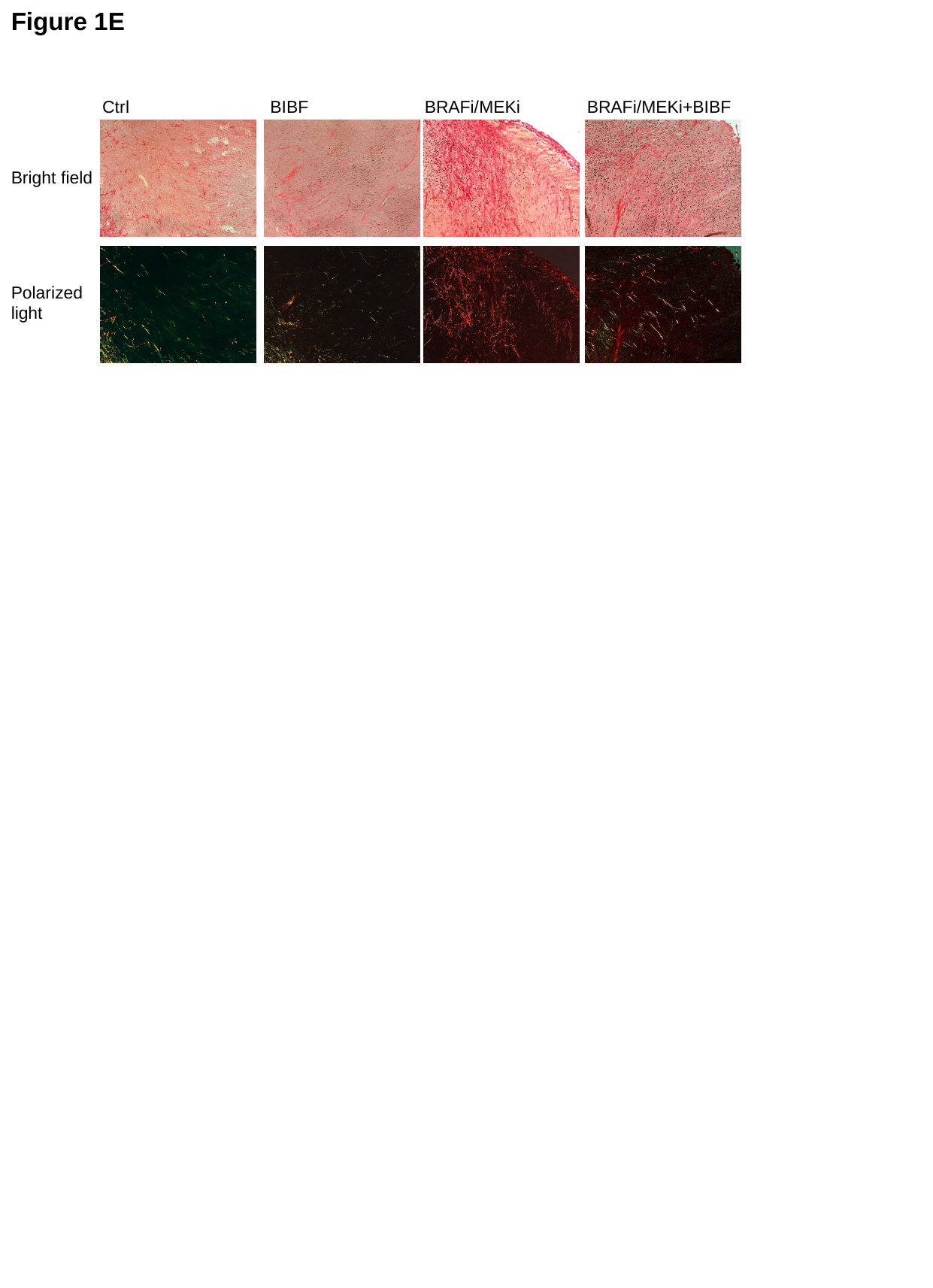

Figure 1E
Ctrl
BIBF
BRAFi/MEKi
BRAFi/MEKi+BIBF
Bright field
Polarized
light

## Slide 2
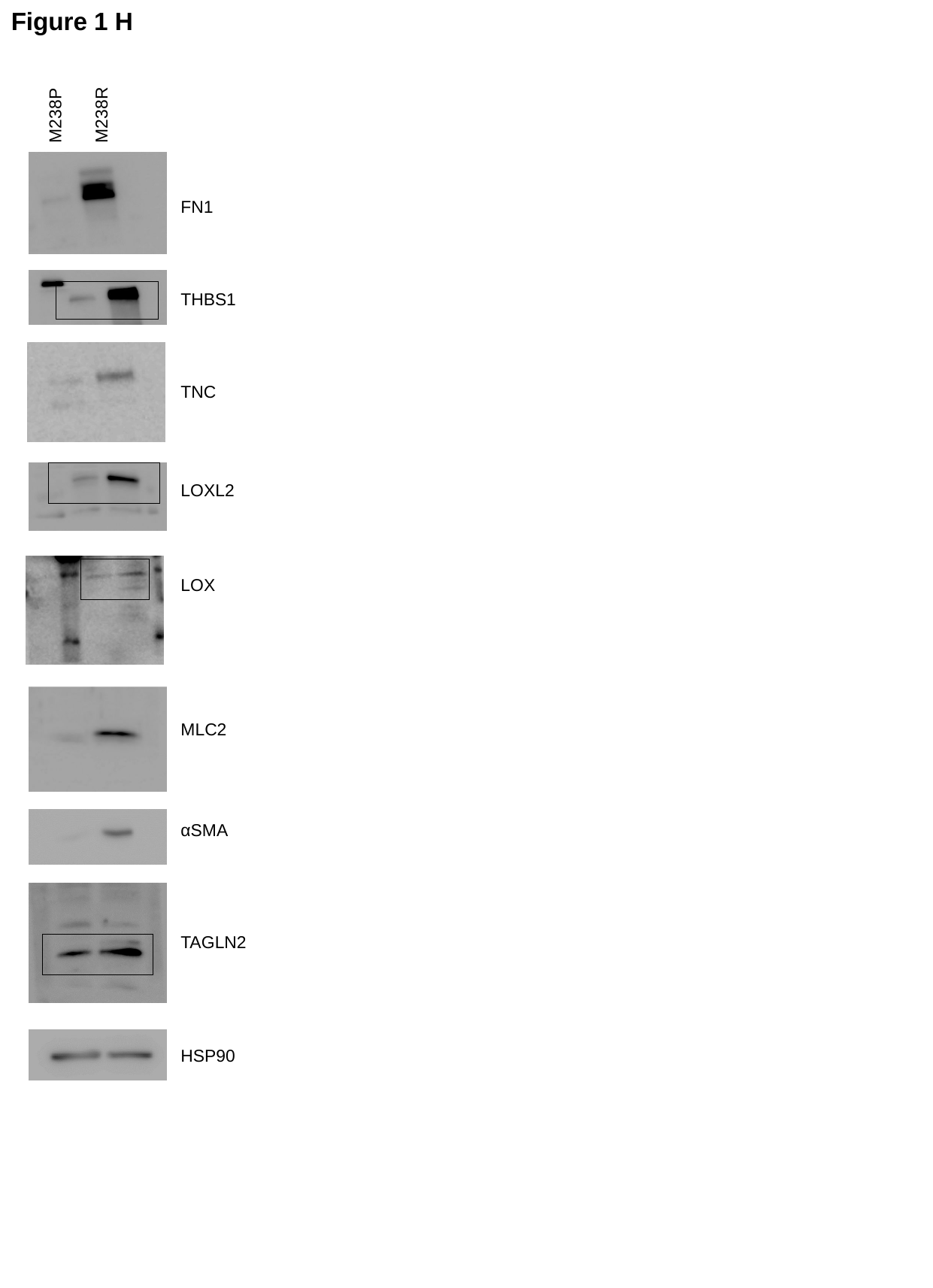

Figure 1 H
M238P
M238R
FN1
THBS1
TNC
LOXL2
LOX
MLC2
αSMA
TAGLN2
HSP90
