# Supplementary material for: Blockade of the pro‐fibrotic reaction mediated by the miR‐143/‐145 cluster enhances the responses to targeted therapy in melanoma
Source: EMBO Mol Med. 2022 Feb 14;14(3):e15295. doi: 10.15252/emmm.202115295 (PMC8899916; doi:10.15252/emmm.202115295)
Supplement: Supplementary file 5 — Source Data for Figure 3 [file EMMM-14-e15295-s007.zip › emmm-202115295-sup-0005-SDataFig3.pptx]

## Slide 1
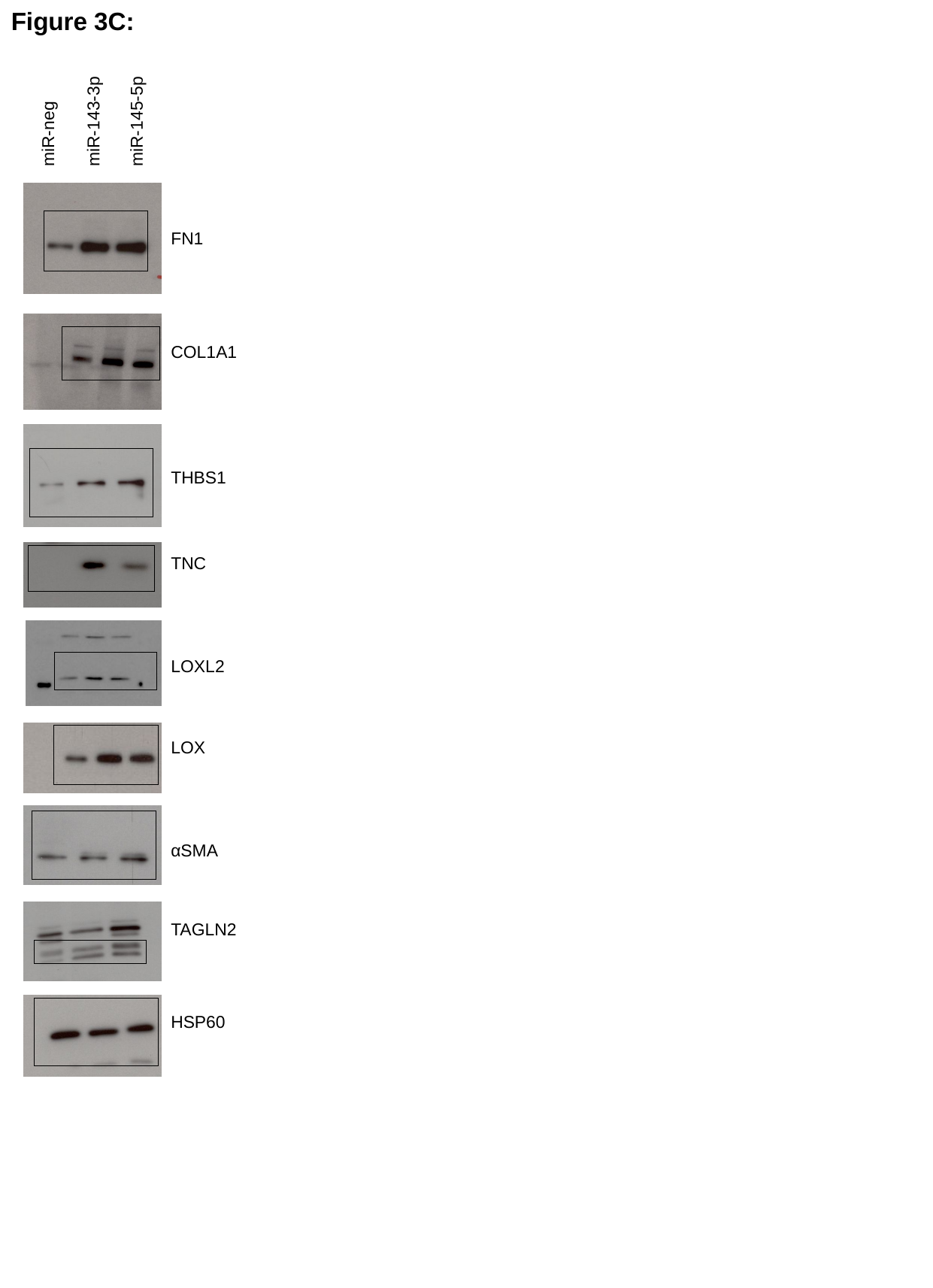

Figure 3C:
miR-neg
miR-143-3p
miR-145-5p
FN1
COL1A1
THBS1
TNC
LOXL2
LOX
αSMA
TAGLN2
HSP60

## Slide 2
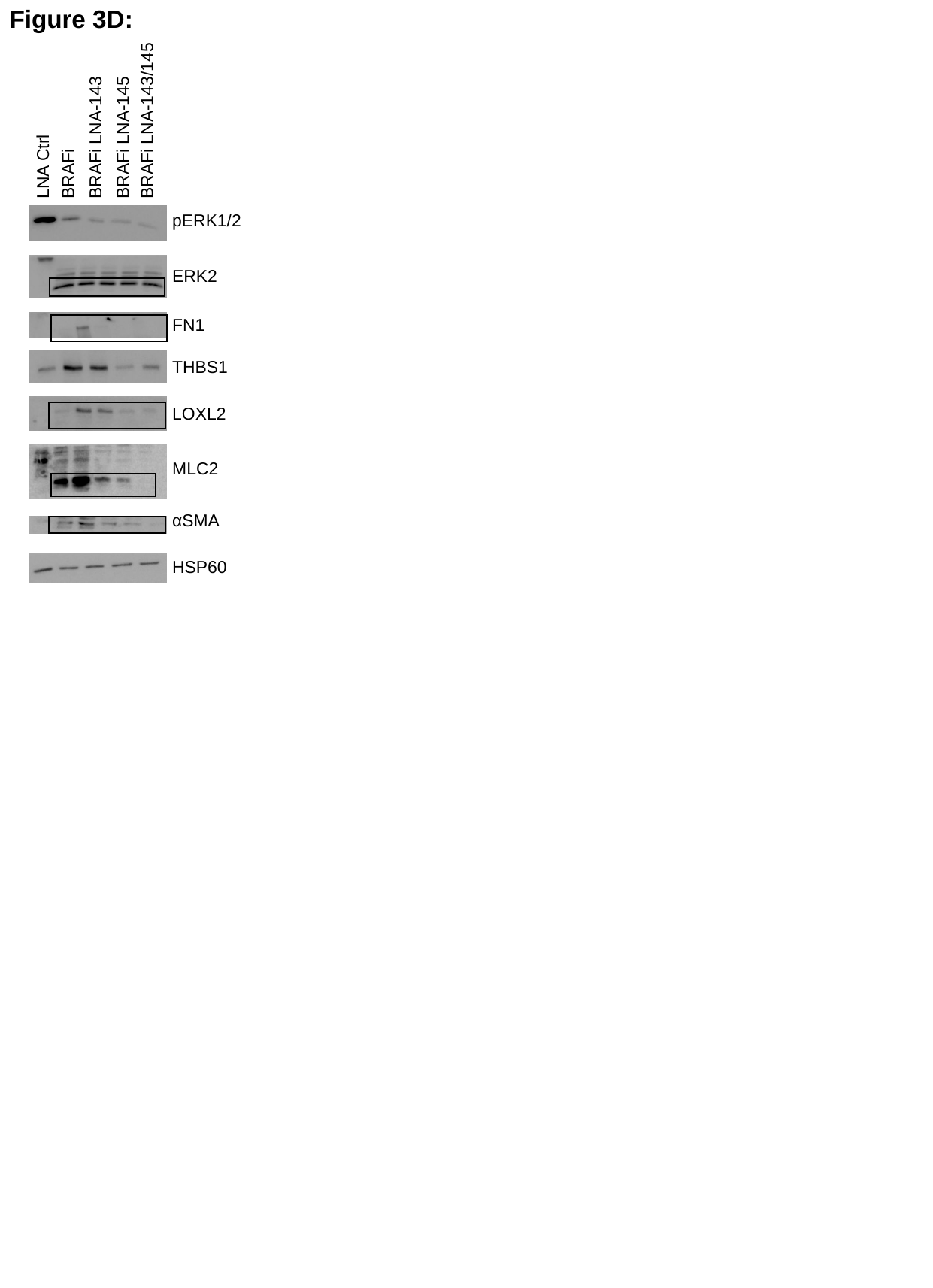

Figure 3D:
BRAFi LNA-143
BRAFi LNA-145
BRAFi LNA-143/145
LNA Ctrl
BRAFi
pERK1/2
ERK2
FN1
THBS1
LOXL2
MLC2
αSMA
HSP60

## Slide 3
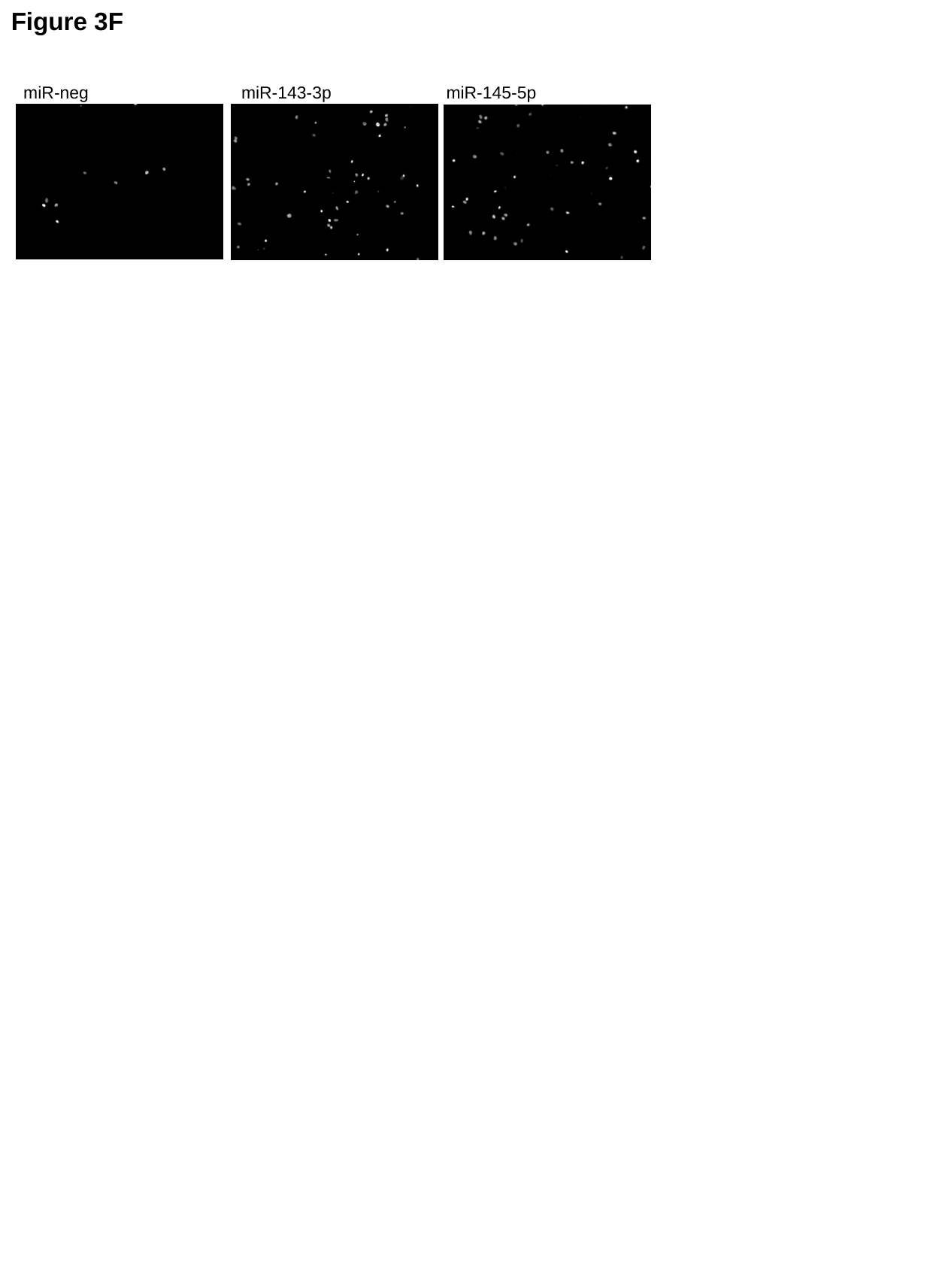

Figure 3F
miR-neg
miR-143-3p
miR-145-5p

## Slide 4
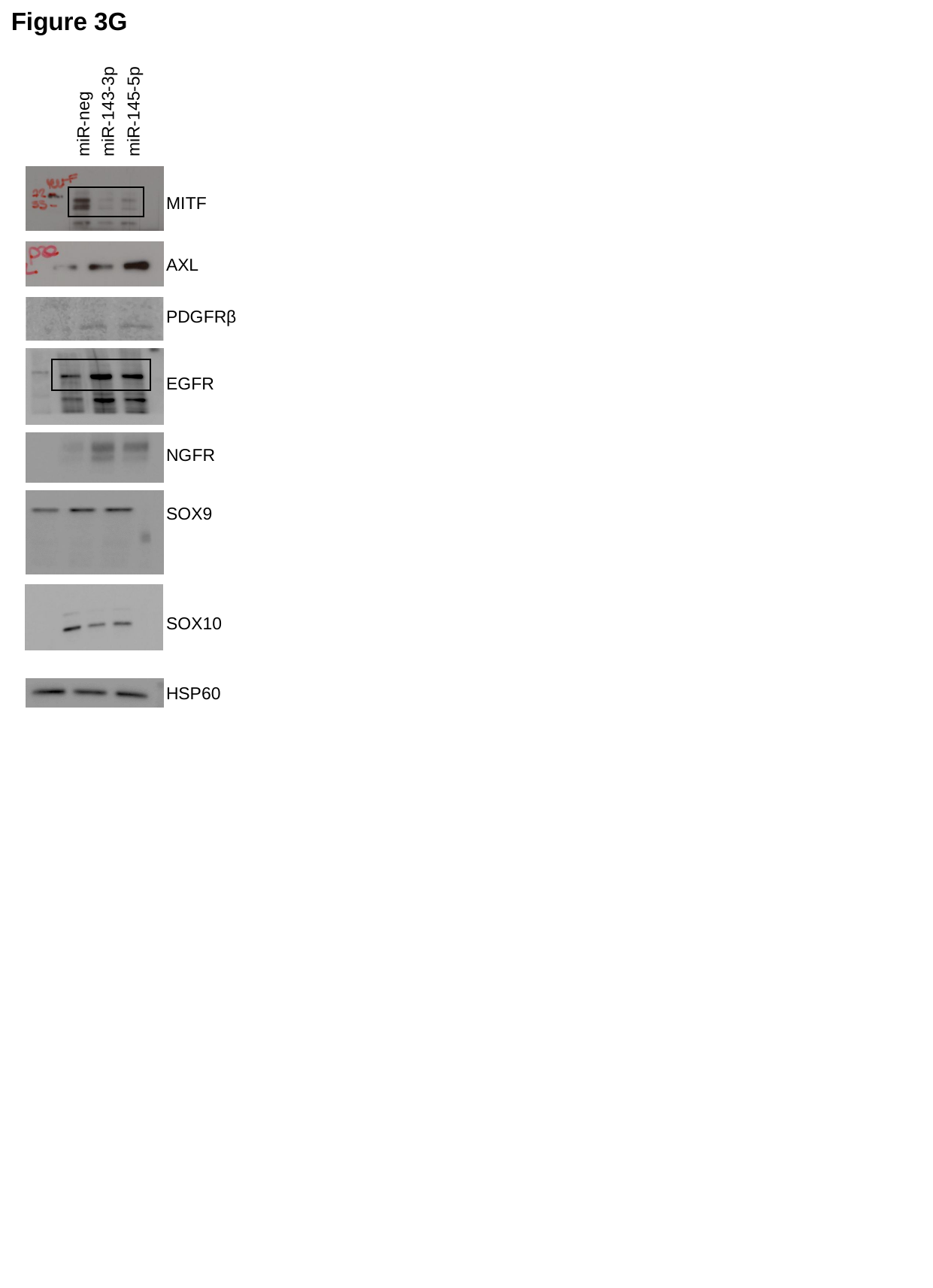

Figure 3G
miR-neg
miR-143-3p
miR-145-5p
MITF
AXL
PDGFRβ
EGFR
NGFR
SOX9
SOX10
HSP60
