# Supplementary figures and images for: Blockade of the pro‐fibrotic reaction mediated by the miR‐143/‐145 cluster enhances the responses to targeted therapy in melanoma
Source: EMBO Mol Med. 2022 Feb 14;14(3):e15295. doi: 10.15252/emmm.202115295 (PMC8899916; doi:10.15252/emmm.202115295)

## Slide 1
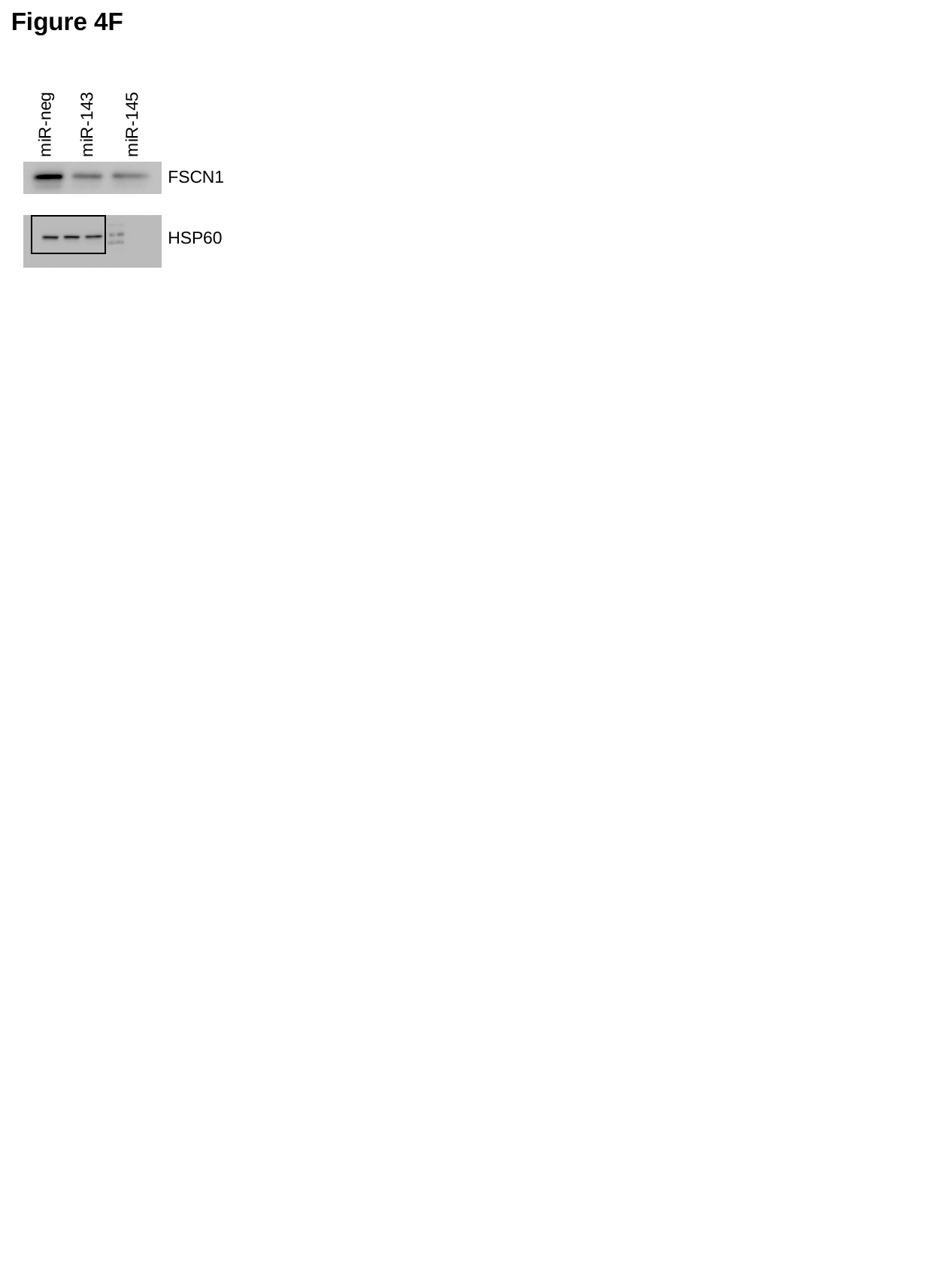

Figure 4F
miR-neg
miR-143
miR-145
FSCN1
HSP60

Supplement: Supplementary file 6 — Source Data for Figure 4 [file EMMM-14-e15295-s004.zip › emmm-202115295-sup-0007-SDataFig4.pptx]
