# Supplementary material for: Blockade of the pro‐fibrotic reaction mediated by the miR‐143/‐145 cluster enhances the responses to targeted therapy in melanoma
Source: EMBO Mol Med. 2022 Feb 14;14(3):e15295. doi: 10.15252/emmm.202115295 (PMC8899916; doi:10.15252/emmm.202115295)
Supplement: Supplementary file 7 — Source Data for Figure 5 [file EMMM-14-e15295-s008.zip › emmm-202115295-sup-0009-SDataFig5.pptx]

## Slide 1
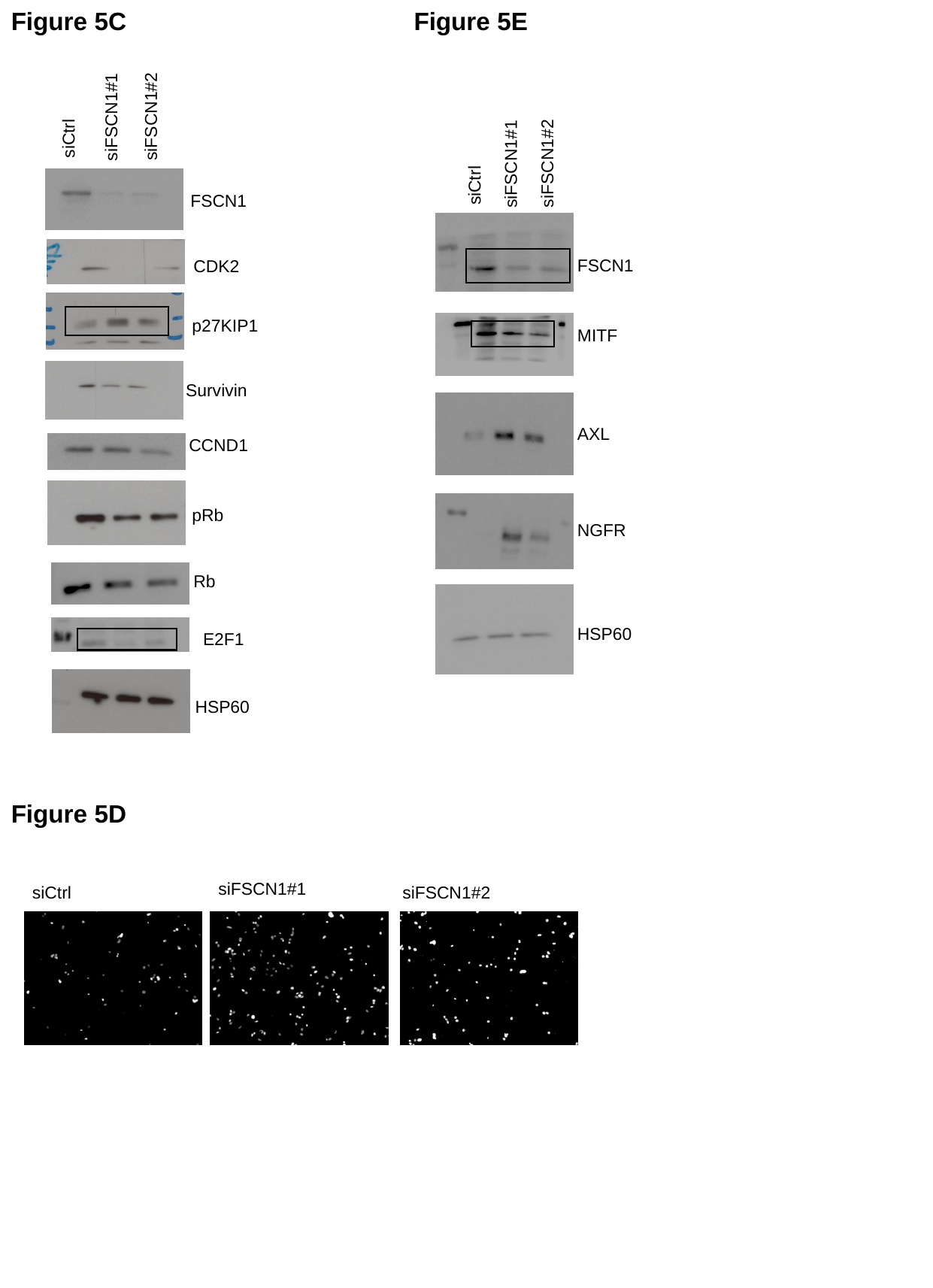

Figure 5C
Figure 5E
siCtrl
siFSCN1#2
siFSCN1#1
siCtrl
siFSCN1#2
siFSCN1#1
FSCN1
FSCN1
CDK2
p27KIP1
MITF
Survivin
AXL
CCND1
pRb
NGFR
Rb
HSP60
E2F1
HSP60
Figure 5D
siFSCN1#1
siCtrl
siFSCN1#2

## Slide 2
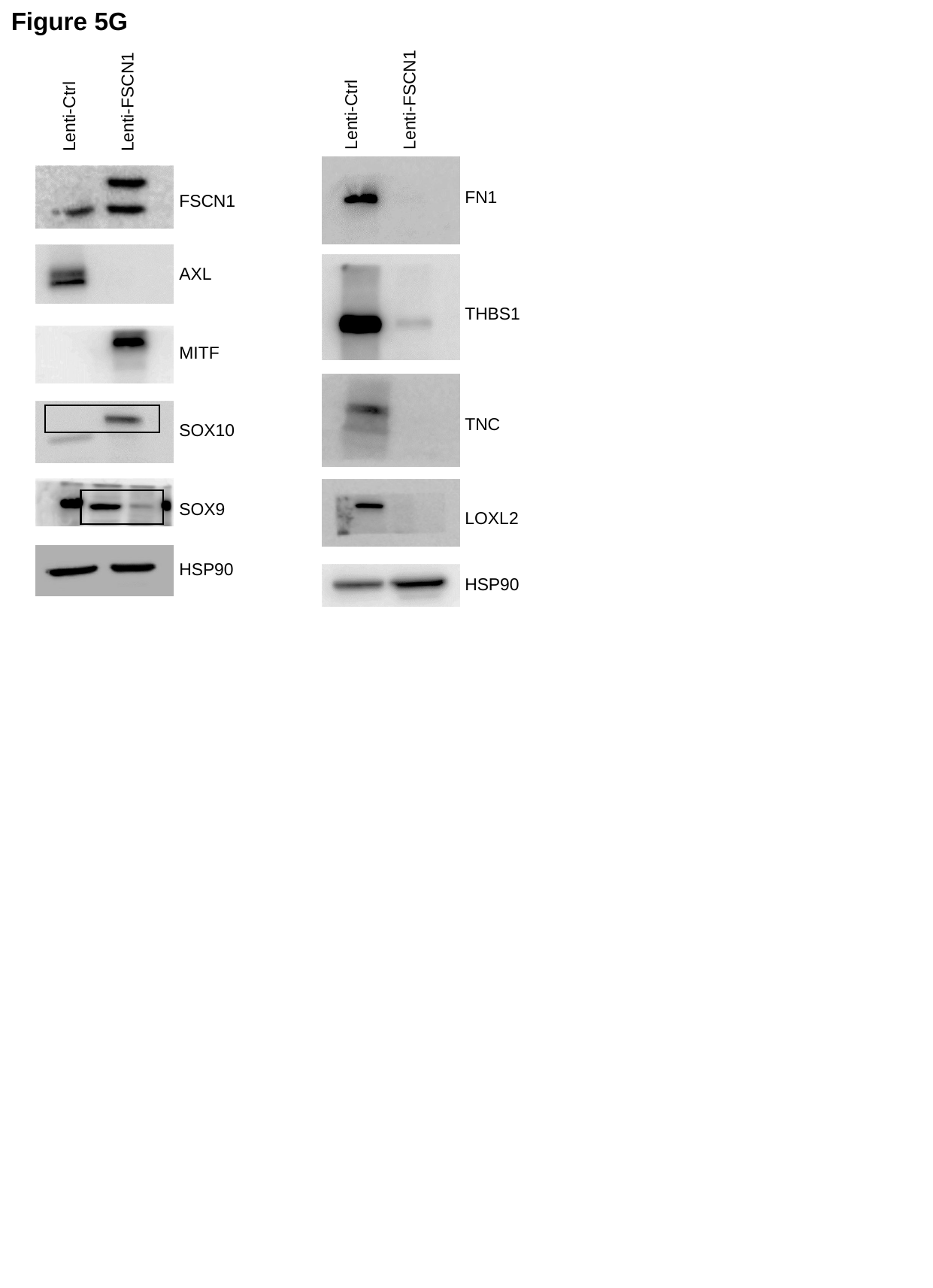

Figure 5G
Lenti-Ctrl
Lenti-FSCN1
Lenti-Ctrl
Lenti-FSCN1
FN1
FSCN1
AXL
THBS1
MITF
TNC
SOX10
SOX9
LOXL2
HSP90
HSP90
