# Supplementary material for: Blockade of the pro‐fibrotic reaction mediated by the miR‐143/‐145 cluster enhances the responses to targeted therapy in melanoma
Source: EMBO Mol Med. 2022 Feb 14;14(3):e15295. doi: 10.15252/emmm.202115295 (PMC8899916; doi:10.15252/emmm.202115295)
Supplement: Supplementary file 10 [file EMMM-14-e15295-s010.pdf]

EMBO Molecular Medicine

Blockade of the pro-fibrotic reaction mediated by the miR-143/-145 cluster enhances the responses to targeted therapy in melanoma

Serena Diazzi, Alberto Baeri, Julien Fassy, Margaux Lecacheur, Oskar Marin-Bejar, Christophe Girard, Lauren Lefevre, Caroline Lacoux, Marie Irondelle, Carine Mounier, Marin Truchi, Marie Couralet, Mickael Ohanna, Alexandrine Carminati, Ilona Berestjuk, Frederic Larbret, David Gilot, Georges Vassaux, Jean-Christophe Marine, Marcel Deckert, Bernard Mari, and Sophie TARTARE-DECKERT
DOI: 10.15252/emmm.202115295

Corresponding authors: Bernard Mari (bernard.mari@unice.fr) , Sophie TARTARE-DECKERT (tartare@unice.fr)

Review Timeline:

Transfer from Review Commons:	16th Oct 21
Editorial Decision:	12th Nov 21
Revision Received:	4th Jan 22
Accepted:	10th Jan 22

Editor: Lise Roth

Transaction Report:

This manuscript was transferred to EMBO Molecular Medicine following peer review at Review Commons

(Note: With the exception of the correction of typographical or spelling errors that could be a source of ambiguity, letters and reports are not edited. Depending on transfer agreements, referee reports obtained elsewhere may or may not be included in this compilation. Referee reports are anonymous unless the Referee chooses to sign their reports.)

Review #1

1. How much time do you estimate the authors will need to complete the suggested revisions:

Estimated time to Complete Revisions (Required)

(Decision Recommendation)

Less than 1 month

2. Evidence, reproducibility and clarity:

Evidence, reproducibility and clarity (Required)

The manuscript is interesting and well presented.
The authors propose the use of an antifibrotic drug to attenuate resistance to RTK inhibitors.

Specific comments

1. It is not entirely clear how Nintedanib decreases tumour growth. It may be due to its effect on resistant melanoma cells as proposed, but it could also be due to the effect on CAFs. This should be at least discussed
2. A potential caveat is that drug used is non-specific as it also blocks PDGFR signalling. Hyperactivation of RTKs is a mechanism of BRAFi resistance and for example in Figure 1J, they see that BIF1120/Nintedanib has a significant effect on BRAFi-resistant cells, which may indicate that the growth inhibition seen in allografts could be a combination of an "anti-fibrotic" role and its own activity inhibiting the survival of resistant cells. This needs to be considered.
3. Does the viability decrease in BRAFi-sensitive cells? For instance, in the parental cells.
4. Figure 1 b-e, in vivo and in vivo experiments. How many animals we used? Collagen decrease is not quantified (statistics missing).
5. The title is not accurate. "prevent" resistance in melanoma is an overestimation because the cells do become resistant, albeit later.

3. Significance:

Significance (Required)

As the authors discussed, they and others have previously studied the contribution of ECM and stromal remodelling to resistance to targeted therapies in melanoma. Previous data from E. Sahai´s lab show that BRAFi activate CAFs and increase the production and remodelling of the extracellular matrix, but in this work, they look at a cell-autonomous mechanism mediated by miRs that promotes fibrosis and propose the use of an antifibrotic drug to attenuate resistance to RTK inhibitors.

Review #2

1. How much time do you estimate the authors will need to complete the suggested revisions:

Estimated time to Complete Revisions (Required)

(Decision Recommendation)

Between 1 and 3 months

2. Evidence, reproducibility and clarity:

Evidence, reproducibility and clarity (Required)

In this very interesting study, Diazzi and colleagues show that during adaptation to MAPK-targeted therapy (MAPKi), melanoma cells upregulate a miRNA profibrotic cluster (miR-143, -145), which drives a phenotypic switch towards a drug resistant undifferentiated mesenchymal-like state. From the miRNA targets, authors identify FSCN1 as a gene that needs to be downregulated during adaptation to MAPKi by the miRNAs, since FSCN1 ablation promotes the drug resistant phenotype. Importantly, authors show in a preclinical mouse melanoma model that the anti-fibrotic drug nintedanib (BIBF) improves response to MAPKi and delays onset of resistance.

The study conclusions are convincing and the data are adequately replicated and presented, authors should be commended for having the manuscript in such good shape. However, there are a few issues that authors should clarify/expand.

1. The study starts with the in vivo YUMM1.7 model and combination BRAFi+MEKi, and then authors use this combination in many in vitro experiments. However, when studying resistant lines, only BRAFi-resistant and -sensitive pairs were used.
I would suggest including more validation of the upregulation of the miRNA and the fibrotic genes on BRAFi+MEKi-resistant lines, and this could be easily gathered from published transcriptomes of several BRAFi+MEKi-resistant melanoma lines from Roger Lo's lab (Song et al 2017 Cancer Discov, including M238, M229, M249 used by the authors). To complement this approach, miRNA expression could be evaluated in large collections of melanoma cell lines classified as more or less undifferentiated (correlating with more or less resistance) as in Tsoi 2018 Cancer Cell and Verfaille 2015 Nat Commun.

Related to this, the clinical relevance would increase if findings were validated using patient samples, for example, from published transcriptomes (Hugo 2015 Cell, Song 2017 Cancer Discov, Wagle 2014 Cancer Discov...) or even from TCGA, which could be used to identify if patients with high miRNA have worse prognosis.

2. While blocking the miRNA improves BRAFi response (Fig.3H), it is not clear that this combination would overcome resistance (using resistant lines), although authors show that BIBF does overcome resistance (Fig.1J).
This also applies to line 277 ".. mirroring the effect of miR143/145 ASOs, forced expression of FSCN1 in M238R cells decreased viability in the presence of BRAFi (Fig.5H)." However, the miRNA ASOs were used in parental cells (Fig.3H).
3. Analysis of cytoskeletal changes. Text (lines 284-287) is missing references, regarding "..morphological changes with cells assuming flattened spindle-like shape" and "..function of FSCN1 in F-actin microfilaments reorganization..".
Besides, authors say that transient overexpression of miRNAs reproduced these morphological changes as shown by F-actin staining. These would have benefited from including also side-by-side comparison of BRAFi treatment on these cell lines. To my knowledge, these melanoma lines (M238, M229, etc) have not been characterized in that regard (F-actin, focal adhesions). In Nazarian et al 2010, only brightfield pictures are shown in a supplementary figure.
The same applies to YAP and especially MRTF activation upon miRNA overexpression, and whether this mirrors what BRAFi does to YAP and MRTF. In Misek et al 2020 and Kim et al 2015 YAP and MRTF were shown to be more enriched in the nucleus in resistant than in parental cells. Kim et al also show in time course experiments that there is significantly higher nuclear YAP after 7-14 days of BRAFi treatment. In the present manuscript, authors seemed to have assessed nuclear YAP/MRTF after 72h miRNA overexpression. Does it mirror MAPKi?
4. Regarding the decreased proliferation/survival after miRNA overexpression, is it truly slow cycling and not combined with some cell death? Table S1 has a "cell death of tumor cell lines" theme after miRNA overexpression.

Related to this, in Supp. Fig.4C the effect on the cell cycle effect is very small, is this significant? It is unclear when the cell cycle was assessed after miRNA overexpression (72h?), it could be a matter of timing. According to Fig.3E, there is a reduction in growth from 60-72h onwards.

5. Statistics. While multiple comparison tests were used, most graphs have asterisks on top of some bars, and it is unclear what is being compared with what. For example, Fig.2B have asterisks on top of BRAFi+MEKi group, does it mean it is significant vs vehicle group? In this and other similar cases (1J, 2C, S1B and others), a comparison against the combination group (BRAFiMEKi+BIBF) is also relevant. This should be revised throughout manuscript.

Minor:

-For all the studies using stable cell lines, authors should state how long after transduction and selection experiments were performed.

-Authors only show single miRNA overexpression or inhibition. However, both miRNA are upregulated upon MAPKi. Did authors try the double overexpression or blockade?

-For the 1205Lu xenograft experiment, authors should also show the tumour growth

curves, and explain how long treatment was and when miRNA expression was analysed (endpoint?). In addition, why in 5A there are only 3 dots (mice?) per group, while in 5B there are more (6-7 in control, 4-5 in BRAFi)?

-In a few graphs, the axis legend should give more information. For example, Fig.2 says Fold change, and it should be Fold change expression, or similar; Fig.4G fold change FSCN mRNA expression; Fig. S2 log2 expression (resistant/par), S5A...

-Fig.1E-G and S1B. Is this at endpoint for each group?

-Fig.3H and S6B. how long were these experiments?
Fig.7B and D. Why the MRTFA signal in miR-neg and siCTRL is so different? Same for UACC in S11A vs s11D.

-Fig.5C and 5E. FSCN1 knockdown in 5C is very efficient, while not so much in 5E. However, effects on MITF, AXL etc in 5C are quite impressive. are these knockdowns representative?

-Fig.6-7 legend. When mentioning scale bar, it reads uM, should it be um?

-Fig.7A. In the graph, the "YAP nuclear enrichment", do the numbers represent the nuclear/cytoplasm ratio?

-When showing migration and a picture (Fig.3F, 5D, S4D, S5E...), the blue over dark background is difficult to see, using greyscale or a brighter pseudocolour would help.

3. Significance:

Significance (Required)

These findings have important preclinical implications, since the study proposes a biomarker of resistance (profibrotic signature) and importantly, a potential new therapy to delay MAPKi resistance in melanoma (BIBF). It could also apply to other BRAFmutant cancers and diseases cursing with fibrosis.

Field of expertise: melanoma, drug resistance, cytoskeleton

Review #3

1. How much time do you estimate the authors will need to complete the suggested revisions:

Estimated time to Complete Revisions (Required)

(Decision Recommendation)

Less than 1 month

2. Evidence, reproducibility and clarity:

Evidence, reproducibility and clarity (Required)

Summary:

In the present work Diazzi and co-authors describe the mechanism through which the anti-fibrotic drug Nintedanib potentiates MAPK-targeted therapy efficacy in melanoma cells. Nintedanib prevents the MAPK-induced pro-fibrotic response and is associated with loss of miR-143/-145 cluster expression. These miRs promote melanoma cells de-differentiation towards a pro-fibrotic mesenchymal-like state that correlates with resistance to MAPK inhibitors. Looking for miR-143/-145 targets responsible for this phenotype switch, the authors identified Fascin1 as a crucial regulator of cytoskeleton dynamics and mechanopathways.

Major comments:

The manuscript is well written, data are convincing, well presented and supportive of the conclusions.

Minor points that may be improved:

- The expression of miR-143/145 increases in melanoma cell lines treated with BRAFi and/or MEKi for 72h (Fig. 2B, Supp. Fig. 2B-F), and also after the development of resistance to MAPK-targeted therapies (Fig. 2A, Supp. Fig. 2A). The transient overexpression of miRs in therapy-naive cells leads to cells de-differentiation toward a mesenchymal/MAPK resistant state. On the other hand, these cells become more sensitive to BRAFi treatment when combined with LNA-mediated inhibition of miRs activity. It would be important to determine if the same occurs also in resistant cells, or whether MAPKi-resistance is established, cells are no longer sensitive to miRs blockade.
- In 2 out of 4 melanoma PDX samples naïve/resistant to combo BRAFi/MEKi therapy, the expression level of miR-143/145 cluster correlates with the de-differentiated transcriptomic profile of resistant tumor. How is Fascin1 expression in these samples?
- The clinical relevance of the data could be strongly improved by assessing the expression of the miRs cluster and of its target Fascin1 in resistant subsets of patients, comparing their expression to patients before treatment, making use of available datasets.

Minor comments:

- Fig. 4C, lower legend: M238P not M238S

3. Significance:

Significance (Required)

Nature and significance of the advances:

The findings not only suggest the combination therapy with the anti-fibrotic drug Nintedanib to be effective in enhancing MAPKi treatment in melanoma, reducing the development of resistance, but identify the molecular mechanism via the induction o the miR-143/145 cluster and the effects on the target Fascin1.

Compare to existing knowledge

These two miRNAs have been shown to have both oncogenic and oncosuppressor activities and have already been involved in EMT induction. The findings add yet one more piece to the puzzle.

Audience

This manuscript is not only of interest for oncology researchers but also of general interest or the understanding of fundamental biological processes and their effects on cancer therapy.

Your expertise

Molecular biologist and cancer research, transcriptional control of tumor transfromatin and progression including EMT, microRNAs -143/145

Full Revision

Manuscript number: RC-2021-00920
Corresponding author(s): Bernard Mari, Sophie Tartare-Deckert

1. General Statements [optional]

First, we would like to sincerely thank the managing Editor and the referees for acknowledging the impact of the study and for their comments as well as helpful and constructive suggestions, which help us to improve our study. We have answered to all of their queries.
In summary, our work highlights miR-143-3p and miR-145-5p as potential targets for overcoming targeted therapy adaptation and inhibiting the expansion of resistant clones, which could be exploited for clinical management of melanoma. This study also provides a rationale for designing clinical trials with clinically approved anti-fibrotic drugs such as Nintedanib in patients treated with targeted therapies. Finally, this work could serve as a paradigm for other cancers that dedifferentiate upon therapy exposure such as breast cancers or glioblastomas and/or display fibrosis-associated properties.
As underlined by the reviewers, our work "is not only of interest for oncology researchers" but also of general interest for the understanding of fundamental biological processes and their effects on cancer therapy". We believe that our findings are of broad interest given the interdisciplinary nature of the manuscript and the hot topics that it covers. Given the importance of improving our understanding of tumor cell dedifferentiation and therapy resistance in cancer, our manuscript can be of great interest to the readers of EMBO Molecular Medicine.
We thank you again for contributing to the quality of our study and look forward to hearing from you. We thank you in advance for your time and consideration of our manuscript.

2. Point-by-point description of the revisions

Reviewer #1 (Evidence, reproducibility and clarity):

The manuscript is interesting and well presented.
The authors propose the use of an antifibrotic drug to attenuate resistance to RTK inhibitors.

Specific comments

1. It is not entirely clear how Nintedanib decreases tumour growth. It may be due to its effect on resistant melanoma cells as proposed, but it could also be due to the effect on CAFs. This should be at least discussed.

The reviewer asks about a potential effect of Nintedanib on CAFs in our mouse model. While we show that Nintedanib has a direct action on melanoma cells *in vitro*, the *in vivo* situation can indeed be more complex. We agree that we cannot rule out the possibility that its therapeutic efficacy could be attributed in part to inhibition of CAFs, knowing that BRAF inhibitors has been shown to activate CAFs in melanoma, generating a host-tumor niche that can mediate therapeutic escape. However, addressing the contribution of CAF *in vivo* is challenging and would represent an entire new study. As requested by the reviewer, we have discussed this important issue and added 3 new references (see discussion section lines 377-381).

2. A potential caveat is that drug used is non-specific as it also blocks PDGFR signalling. Hyperactivation of RTKs is a mechanism of BRAFi resistance and for example in Figure 1J, they see that BIF1120/Nintedanib has a significant effect on BRAFi-resistant cells, which may indicate that the growth inhibition seen in allografts could be a combination of an "anti-fibrotic" role and its own activity inhibiting the survival of resistant cells. This needs to be considered.

We thank the reviewer for this interesting issue. Nintedanib was chosen due to its inhibitory action on extracellular matrix deposition and as an example of a rapidly available drug to be exploited therapeutically to increase the effect of targeted therapy and delay the emergence of therapy-resistant cells. We recognize that a possible disadvantage of Nintedanib could be due to its multi-targeted nature (e.g. PDGFR (α and β), FGFR-1, -2, -3, -4 and VEGFR-1, -2, -3 as well as Src, Lck or Lyn) but it is one of the only approved molecules for the treatment of fibroproliferative diseases. Upregulation of PDGFRβ/AKT signaling was previously shown to contribute to acquired resistance in M238R (Shi et al. Cancer Res. 2011;71:5067-74 ; Nazarian et al. Nature. 2010;468:973-7). Our *in vitro* results indicate that Nintedanib inhibits survival of these resistant cells along with a decrease in their myofibroblast-like dedifferentiated phenotype (Fig. 1 I-J).

To meet the reviewer's comment, we have now addressed the contribution of PDGFRβ inhibition in Nintedanib's effects on resistant cells. We have performed experiments on M238R using the selective PDGFR inhibitor CP673451 in comparison with Nintedanib (please see results section lines 120-127 and new Supplementary Fig. S1F-H). The data show that selective inhibition of the PDGFR pathway attenuates the myofibroblast-like signature typical of resistant cells to a similar degree as Nintedanib and affects melanoma cell viability (new Supplementary Fig. S1G-H). However, administration of CP673451 showed less efficiency than Nintedanib in inducing a phenotype switch toward a more differentiated phenotype (new Supplementary Fig. S1G). To further confirm the implication of RTK pathway in the phenotype observed, we analyzed the tyrosine phosphorylation status of EGFR, PDGFR and FGFR (another RTK inhibited by Nintedanib) and activation of AKT in M238R melanoma cells upon treatment with Nintedanib or CP673451 (new Supplementary Fig. S1F and Figure below). Nintedanib had no effect on FGFR tyrosine phosphorylation and slightly decreased pEGFR levels. However, we found that the two inhibitors showed similar efficiency in decreasing phospho-PDGFRβ and phospho-AKT levels (Supplementary Fig. S1F). The results section has been modified according to these new results (lines 126-127).

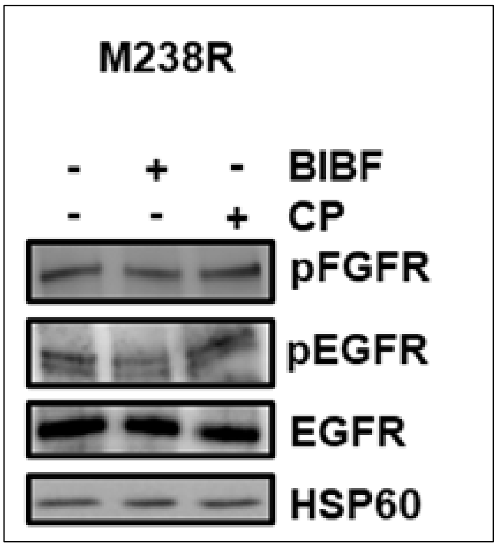

Effect of CP673451 and BIBF1120 on FGFR and EGFR phosphorylation.
Immunoblot analysis of FGFR and EGFR phosphorylation in resistant cells (M238R) treated with BIBF1120 (2 µM, 72h) or CP673451 (2 µM, 72h). HSP60 is used as loading control.

Altogether these data suggest that inhibition of PDGFR signaling likely plays a prominent role in the efficacy of Nintedanib *in vitro* on M238R survival. Thus, as proposed by the reviewer, we can predict that the growth inhibition induced by Nintedanib seen *in vivo* could be a combination of its "anti-fibrotic" action and PDGFR inhibitory activity inhibiting the survival of resistant cells. It is important to note that, compared to Nintedanib, inhibition of PDGFR/AKT signaling by the CP673451 compound is not sufficient to direct melanoma cells to a more differentiated state. This is now discussed in the manuscript (Discussion section lines 404-405).

3. Does the viability decrease in BRAFi-sensitive cells? For instance, in the parental cells?

This information was already addressed in the manuscript. As shown in Supplemental Fig. S1D, Nintedanib had no effect on BRAFi-sensitive M238P viability. We have also confirmed this result using a crystal violet viability assay on M238P and UACC62 cells treated with different doses of BIBF1120 (Please see figure below).

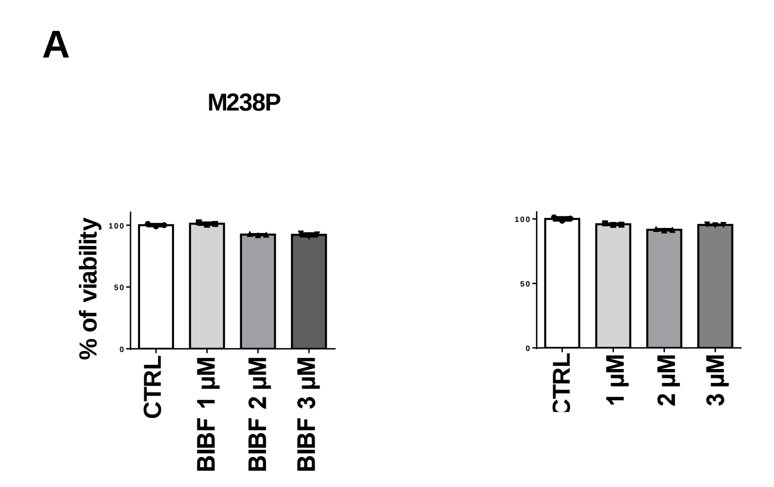

Effect of BIBF1120 treatment on parental BRAFi-sensitive cells. Viability of M238P (A) or UACC62P (B) cells was assessed by crystal violet staining upon 72 h BIBF1120 treatment at the indicated doses.

4. Figure 1 b-e, in vivo and in vivo experiments. How many animals were used? Collagen decrease is not quantified (statistics missing).

We apologize for this omission and have now added the number of animals in the legend of Fig.1 (n = 6). We have also performed statistics for collagen quantification and included this analysis in Fig.1F (see lines 720/723). We also provide to the referee the detailed statistical analysis of mature collagen fibers between the different treatment groups, as shown below.

Statistical analysis of mature collagen fibers (red)	BIBF1120	BRAFi/MEKi	BRAFi/MEKi +BIBF1120
Ctrl	ns	****	***
BIBF1120		****	*
BRAFi/MEKi			**

Statistics for mature collagen fibers quantification in the different treatment groups. Two-way ANOVA was used for statistical analysis. $*P \leq 0.05$, $**P \leq 0.01$, $***P \leq 0.001$, $****P \leq 0.0001$.

5. The title is not accurate. "prevent" resistance in melanoma is an overestimation because the cells do become resistant, albeit later.

We agree with the reviewer and we have modified the title accordingly. The new title is now: "Blockade of pro-fibrotic response mediated by the miR-143/-145 cluster prevents targeted therapy-induced phenotypic plasticity and **delays** resistance in melanoma".

Reviewer #1 (Significance):

As the authors discussed, they and others have previously studied the contribution of ECM and stromal remodelling to resistance to targeted therapies in melanoma. Previous data from E. Sahai´s lab show that BRAFi activate CAFs and increase the production and remodelling of the extracellular matrix, but in this work, they look at a cell-autonomous mechanism mediated by miRs that promotes fibrosis and propose the use of an antifibrotic drug to attenuate resistance to RTK inhibitors.

Reviewer #2 (Evidence, reproducibility and clarity (Required)):

In this very interesting study, Diazzi and colleagues show that during adaptation to MAPK-targeted therapy (MAPKi), melanoma cells upregulate a miRNA profibrotic cluster (miR-143, -145), which drives a phenotypic switch towards a drug resistant undifferentiated mesenchymal-like state. From the miRNA targets, authors identify FSCN1 as a gene that needs to be downregulated during adaptation to MAPKi by the miRNAs, since FSCN1 ablation promotes the drug resistant phenotype. Importantly, authors show in a preclinical mouse melanoma model that the anti-fibrotic drug nintedanib (BIBF) improves response to MAPKi and delays onset of resistance.

The study conclusions are convincing and the data are adequately replicated and presented, authors should be commended for having the manuscript in such good shape. However, there are a few issues that authors should clarify/expand.

We sincerely thank the reviewer for his/her careful review and constructive comments.

1. The study starts with the in vivo YUMM1.7 model and combination BRAFi+MEKi, and then authors use this combination in many in vitro experiments. However, when studying resistant lines, only BRAFi-resistant and -sensitive pairs were used.
I would suggest including more validation of the upregulation of the miRNA and the fibrotic genes on BRAFi+MEKi-resistant lines, and this could be easily gathered from published transcriptomes of several BRAFi+MEKi-resistant melanoma lines from Roger Lo's lab (Song et al 2017 Cancer Discov, including M238, M229, M249 used by the authors). To complement this approach, miRNA expression could be evaluated in large collections of melanoma cell lines classified as more or less undifferentiated (correlating with more or less resistance) as in Tsoi 2018 Cancer Cell and Verfaille 2015 Nat Commun.

We thank the reviewer for these interesting suggestions. We have performed several analyses, summarized below:
- First, we have analyzed the expression of the miRNA-143/-145 cluster and pro-fibrotic signature by qPCR in A375 parental and BRAFi/MEKi double resistant melanoma cell lines described in Shen *et al.* Nat Commun. 2019;10:5713. We observed the upregulation of both mature miRNAs along with a pro-fibrotic signature in several A375 DR clones compared to

parental cells. This new result is described in the results section (lines 147-150) and shown in new Supplementary Fig. S2B. In addition, we have included in the results section the important information that the undifferentiated/mesenchymal-like BRAFi-resistant M229R and M238R cells used in our work also displayed cross-resistance to MEKi (results section, line 112 and 1 new reference).

- Second, as recommended, we have also fully (re)analyzed the mentioned studies and associated datasets. We provide below a summary of the different studies including samples number, design of the study, platform used and accession.

Study	Results	Number of samples	Technique	GEO access nb
Song et al. 2017, Cancer Discovery	• MIR143HG up-expressed gene in MAPK-redundant R-lines (Rr)	• 18 isogenic P-lines versus R-lines (SDR or DDR) ;	RNA-seq platform in table S1B	GSE75313
	• MIR143HG in the Rr specific signature	• Patient-matched melanoma tumors (total n = 46) before MAPKi therapies (BRAFi, MEKi, or BRAFi + MEKi) at baseline (n = 20) and On-Tx (n = 26; 23 of 26 biopsies on day 22 or earlier) after objective clinical response		
Tsoi et al. 2018 Cancer Cell	• No MIR143HG expression in the differential expressed genes	53 human melanoma cell lines including paired acquired resistance sublines, established from patient biopsies	Illumina HiSeq2500 (GPL16791)	• RNASeq of melanoma cell lines GSE80829
	• FSCN1 FPKM downregulated in several acquired resistant cell lines (M229AR, M238AR, M376AR, M395AR, M397AR)			• RNASeq of vemu treatment timecourse GSE110054
Verfaillie et al. 2015 Nature Communications	• No MIR143HG expression detected in the dataset	11 short-term cultures derived from patient biopsies	Illumina HiSeq2000 (GPL11154)	GSE60666
	• FSCN1 not linked to the proliferative or invasive phenotype			
Hugo et al. 2015 Cell	•No miR-143, miR-145 or MIR143HG expression in the dataset	90 specimens of patient-derived melanoma tissues	Illumina HiSeq 2000 (Homo sapiens) GPL11154	GSE65186
Tirosh et al. 2016 Science	• MIR143HG upregulated in 4 out of 6 patients post MAPKi (bulk RNA seq)	• 6 patients pre and post MAPKi (Bulk RNA seq)	Illumina HiSeq 2500 (GPL16791)	GSE77940 (bulk RNA seq)
	• FSCN1 downregulated in 2 out of 6 patients post MAPKi (bulk RNA-seq)	• scRNA-seq data performed on stromal microenvironment only (FACS sorting)	Illumina NextSeq 500 (GPL18573)	GSE72056 (scRNA-seq)

List and summary of main transcriptomic studies available.

A general observation is that unfortunately, none of these published studies provided an available small RNA-seq dataset, which thus does not allow quantifying the expression levels of mature miRNAs. However, some interesting observations have been uncovered from these datasets, confirming at least in part some of our data:

i) The dataset from Song *et al.* 2017 compared 18 isogenic parental versus resistant cell lines. Two subsets of resistant cells were identified, with MAPK addiction (Ra) or Resistance with MAPK redundancy (Rr). The expression of the pri-miR-143/145 precursor, named MIR143HG, was detected in these cells and was found significantly upregulated in Rr cell lines compared to parental cells. Of note, MIR143HG was also part of the Rr specific signature associated with a mesenchymal phenotype. This interesting observation is now discussed in the manuscript (Discussion section, lines 392-394).

ii) The dataset from Tsoi *et al.* 2018 focused on transcriptome analysis of 53 human melanoma cell lines including paired acquired resistance sublines established from patient biopsies. Unfortunately, MIR143HG expression is not detected in this dataset, probably due to a limited sequencing depth. Interestingly, we found that FSCN1 expression was decreased in most mesenchymal-like resistant cell lines compared to their parental counterpart. These data cannot be added in the manuscript since we cannot correlate the expression of the miRNAs with their target.

iii) The dataset from Verfaillie *et al*. 2015 revealed transcriptomic analyses on 11 short-term cultures derived from patient biopsies before therapy and gave access to RNA-seq data of tumors with a proliferative or an invasive phenotype. MIR143HG is not detected and FSCN1 expression does not appear to be associated with a specific phenotype. We have performed qPCR-based expression of miR-143-3p and miR-145-5p in some of these short-term cultures, confirming that miR-143/-145 expression is not associated with a specific phenotype in therapy naïve melanoma cells (results for referees, see below). Expression of miR-143-3p and miR-145-5p in each short-term culture was compared to the average expression of the analyzed miRNA in the proliferative short-term cultures. These results are consistent with the findings of our study describing that expression of the miR-143/145 cluster is triggered by the inhibition of the BRAF oncogenic pathway.

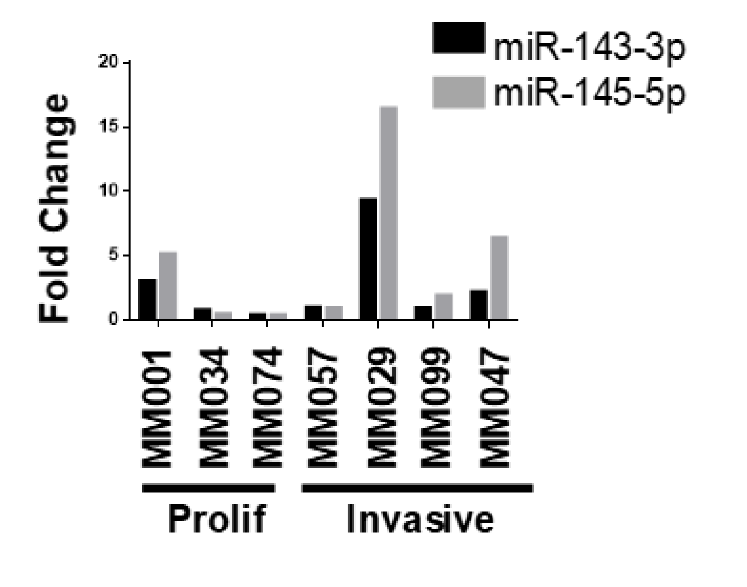

Expression of miR-143-3p and miR-145-5p in short-term cultures derived from patient biopsies. Relative miRNA expression levels were quantified by RT-qPCR.

Related to this, the clinical relevance would increase if findings were validated using patient samples, for example, from published transcriptomes (Hugo 2015 Cell, Song 2017 Cancer Discov, Wagle 2014 Cancer Discov...) or even from TCGA, which could be used to identify if patients with high miRNA have worse prognosis.

We agree with the reviewer about the importance of providing clinical data supporting our observations. We have carefully analyzed all these profiling studies and provide below a summary.
Overall, these studies have several limitations: i) as underlined above, expression of the miRNA cluster is specifically induced in response to therapy and is not present (or barely) in tumors at diagnosis; ii) no small RNA-seq datasets are available yet; iii) melanoma tumors are highly heterogeneous and invaded with stroma, especially CAFs and vessels that also express these miRNAs. We have looked at the expression of the MIR143HG precursor in these datasets and it was not present, probably due to low to medium sequencing depths in these clinical studies.
We have also carefully explored TCGA datasets to look at possible association between prognosis and mature / precursor miRNA as well as miRNA target (FSCN1) expression in skin cutaneous melanoma (SKCM) using the tools developed by Anaya *et al.* 2016, PeerJ

Computer Science 2:e67. Cox regression models and Kaplan-Meier analysis (using different percentiles) did not show any association of our candidates with survival on a cohort of 459 SKCM patients (median survival of 2.4 years, see Kaplan plots below).

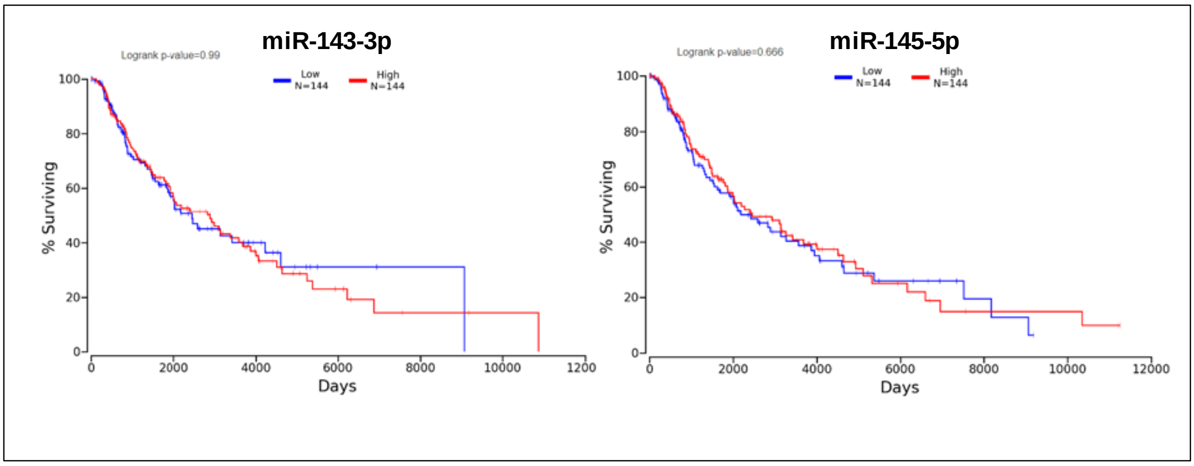

Kaplan–Meier analyses of correlations between miR-143-3p and miR-145-5p expression level and overall survival of TCGA SKCM patients. P-values were calculated using the logrank test.

Finally, during the revision process, we could have access to 9 relapsed melanoma for research purposes from the Dermatology Department of Nice University Hospital (CHU) following treatment with targeted therapies, immunotherapies or a combination of them. We have analyzed in these biopsies the expression of fibrotic/mesenchymal genes, FSCN1 and the miR-143/145 cluster compared to the mean expression of the same genes/miRNAs in therapy naïve patient-derived xenografts (MEL003, MEL006, MEL015, MEL047). Our first results indicate that relapsed tumors acquire a strong fibrotic signature which is associated to increased expression of the miR-143/-145 cluster and decreased expression of FSCN1 (8 out of 9 patients, see results for referees below).

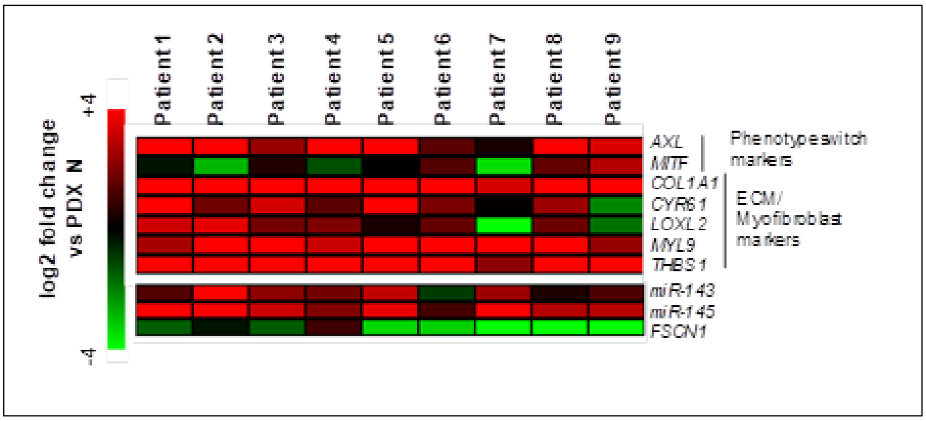

Expression of miR-143-3p, miR-145-5p, FSCN1 and mesenchymal markers in biopsies from melanoma patients progressing on therapies. Relative gene expression and miRNA expression levels were quantified by RT-qPCR.

These results are encouraging and represent a good indicator for further clinical validation but are not solid enough to be incorporated in the manuscript. Overall, validation of our hypotheses in patient samples would require an entire new and highly complex clinical study comparing

tumors at diagnosis with relapsed tumors after targeted therapies and ideally processed using single-cell RNA-seq and/or RNA FISH to take into account the stromal compartment.

2. While blocking the miRNA improves BRAFi response (Fig.3H), it is not clear that this combination would overcome resistance (using resistant lines), although authors show that BIBF does overcome resistance (Fig.1J).
This also applies to line 277 "… mirroring the effect of miR143/145 ASOs, forced expression of FSCN1 in M238R cells decreased viability in the presence of BRAFi (Fig.5H)." However, the miRNA ASOs were used in parental cells (Fig.3H).

To meet the reviewer's comment, we have conducted new experiments in resistant melanoma cells using different approaches to silence simultaneously the 2 mature miRNAs: i) an ASO-directed RNAse H degradation of the miR-143/145 precursor, as described by Plaisance *et al*., JACC Basic Transl Sci. 2016, 1:472-493 to knock-down the pri-miRNA in cardiomyocytes, and ii) a combination of the 2 anti-miRs ASOs. Unfortunately, the first approach failed to efficiently inhibit the expression of mature miR-143-3p and miR-145-5, suggesting that the miR-143/145 cluster has a different precursor gene in melanoma than the one described in cardiomyocytes. Concerning the second approach, as expected, the 2 anti-miRs ASOs as well as the combination of the 2 ASOs efficiently targeted the mature miRNAs (new Supplementary Fig.S6C). Inhibition of miR-145-5p alone and combined inhibition of the two miRNAs significantly affected the viability of BRAFi resistant melanoma cells (M238R) in the absence of BRAFi (new Supplementary Fig.S6D) in a similar way as Nintedanib/BIBF (Fig. 1J).

3. Analysis of cytoskeletal changes. Text (lines 284-287) is missing references, regarding "…morphological changes with cells assuming flattened spindle-like shape" and "..function of FSCN1 in F-actin microfilaments reorganization...".

We apologize for these omissions and have added the relevant references in the text (lines 305/306).

Besides, authors say that transient overexpression of miRNAs reproduced these morphological changes as shown by F-actin staining. These would have benefited from including also side-by-side comparison of BRAFi treatment on these cell lines. To my knowledge, these melanoma lines (M238, M229, etc) have not been characterized in that regard (F-actin, focal adhesions). In Nazarian et al 2010, only brightfield pictures are shown in a supplementary figure.
The same applies to YAP and especially MRTF activation upon miRNA overexpression, and whether this mirrors what BRAFi does to YAP and MRTF. In Misek et al 2020 and Kim et al 2015 YAP and MRTF were shown to be more enriched in the nucleus in resistant than in parental cells. Kim et al also show in time course experiments that there is significantly higher nuclear YAP after 7-14 days of BRAFi treatment. In the present manuscript, authors seemed to have assessed nuclear YAP/MRTF after 72h miRNA overexpression. Does it mirror MAPKi?

As suggested by the reviewer, we have compared side-by-side the effect of oncogenic MAPK pathway inhibition to the effect of miR-143 or miR-145 overexpression on cytoskeleton and focal adhesion dynamics as well as YAP and MRTFA nuclear translocation in M238P, M229P and UACC62P melanoma cells. These analyses clearly show that transient overexpression of miR-143-3p or miR-145-5p mirrors the effects of BRAF or BRAF/MEK inhibition after 3 days on mechanopathways and acto-myosin remodeling. We thank the referee for this comment,

which is helpful for the interpretation of the data. The new additional panels have been included in new Fig. 6B-D, new Fig. 7B-D, new Supplementary Fig. S10B-D and new Supplementary Fig. S11C-D.

4. Regarding the decreased proliferation/survival after miRNA overexpression, is it truly slow cycling and not combined with some cell death? Table S1 has a "cell death of tumor cell lines" theme after miRNA overexpression.

Following the reviewer suggestion, Annexin V/DAPI staining has been performed in M238P cells upon transient overexpression of miR-143 or miR-145. No significant cell death was observed (new Supplementary Fig. S4D). Detailed statistical analysis and quantification of the experiment is provided below (panel A). Staurosporine (Stauro) treatment was used as a positive control of cell death induction.

Related to this, in Supp. Fig.4C the effect on the cell cycle effect is very small, is this significant? It is unclear when the cell cycle was assessed after miRNA overexpression (72h?), it could be a matter of timing. According to Fig.3E, there is a reduction in growth from 60-72h onwards.

We performed, as suggested by the reviewer, cell cycle analysis at longer timing after transfection (96 hours) (new Supplementary Fig. S4C). We observed a significant accumulation of melanoma cells in G0/G1 phase upon miR-143 or miR-145 overexpression and a significant decrease of the percentage of cells in S phase. Detailed statistical analysis of the described experiment is shown below (panel B).

A

	Viable %	Annexin V+ %	DAPI + %	Annexin V+/DAPI+ %
miR-neg	98.1	1	0.6	0.08
miR-143	98	2.1	0.1	0.04
miR-145	98.6	1.1	0.2	0.04
Stauro	5.3	19.28	4.26	71.16

B

	G0/G1	S	G2/M
miR-143 vs miR-neg	****	****	****
miR-145 vs miR-neg	****	****	ns

Detailed statistics analysis of the experiments shown in Fig.S4D (A) and Fig. S4C (B).

Statistics. While multiple comparison tests were used, most graphs have asterisks on top of some bars, and it is unclear what is being compared with what. For example, Fig.2B have asterisks on top of BRAFi+MEKi group, does it mean it is significant vs vehicle group? In this and other similar cases (1J, 2C, S1B and others), a comparison against the combination group (BRAFiMEKi+BIBF) is also relevant. This should be revised throughout manuscript.

As recommended by the reviewer, statistical analysis have been modified in the mentioned figures: Fig. 1J (lines 732/733), Fig. 2B (lines 745/746), Fig. 2C (lines 749/750) and Fig. S1B (see new figures and lines 251/252 of Supplementary materials).

Minor:

-For all the studies using stable cell lines, authors should state how long after transduction and selection experiments were performed.

As recommended, we have now added this information (see lines 8-12 of Supplementary materials).

- Authors only show single miRNA overexpression or inhibition. However, both miRNA are upregulated upon MAPKi. Did authors try the double overexpression or blockade?

As suggested by the reviewer, we experimented the double blockade in M238P and 1205Lu cells treated with MAPK inhibitors. Results are presented in new Fig. 3B, 3D, 3H and Supplementary Fig. S6A-B. Overall, combined inhibition of the two miRNAs had an effect comparable or more significant than the single miRNA inhibition depending on the cellular parameter analyzed.
Concerning the double overexpression, we already experimented lentivirus-mediated stable overexpression of the two miRNAs in two melanoma cell lines. Results are presented in Supplementary Fig. S5A-F and confirmed the functional effects observed by the single miRNA overexpression.

- For the 1205Lu xenograft experiment, authors should also show the tumour growth curves, and explain how long treatment was and when miRNA expression was analysed (endpoint?). In addition, why in 5A there are only 3 dots (mice?) per group, while in 5B there are more (6-7 in control, 4-5 in BRAFi)?

We apologize for this omission. We have added line 270 of the manuscript the reference to the previous study in which the experiment is described. miRNA expression was analyzed in tumors at the endpoint of the experiment i.e. 2 weeks after Vemurafenib treatment start. Moreover, we performed again the analysis of FSCN1 and miR-143/145 expression with the same number of mice (n = 6), please see new Fig. 5A.

- In a few graphs, the axis legend should give more information. For example, Fig.2 says Fold change, and it should be Fold change expression, or similar; Fig.4G fold change FSCN mRNA expression; Fig. S2 log2 expression (resistant/par), S5A...

We have corrected this and modified y-axis legends in the corresponding figures.

- Fig.1E-G and S1B. Is this at endpoint for each group?

Yes, it is as stated in the materials and methods section.

- Fig.3H and S6B. how long were these experiments?

Experiments shown in Fig. 3H and Fig. S6B were carried out during 72 h. This information has been included in the legend of the corresponding figures.

- Fig.7B and D. Why the MRTFA signal in miR-neg and siCTRL is so different? Same for UACC in S11A vs s11D.

We apologize for this inaccuracy. We have revised the figures to show more representative pictures (new Figs. 7B, 7D and S11A, S11D and new Fig. 6C).

- Fig.5C and 5E. FSCN1 knockdown in 5C is very efficient, while not so much in 5E. However, effects on MITF, AXL etc in 5C are quite impressive. are these knockdowns representative?

We again apologize for this inaccuracy. We performed a new experiment and we are now showing a more representative FSCN1 knockdown in new Fig. 5E.

- Fig.6-7 legend. When mentioning scale bar, it reads uM, should it be um?

We have corrected this mistake.

- Fig.7A. In the graph, the "YAP nuclear enrichment", do the numbers represent the nuclear/cytoplasm ratio?

Yes, numbers represent the nuclear/cytoplasm ratio. This information was added in the legend of the corresponding figures.

- When showing migration and a picture (Fig.3F, 5D, S4D, S5E...), the blue over dark background is difficult to see, using greyscale or a brighter pseudocolour would help.

We thank the reviewer for this useful suggestion. We have done this and used the gray scale to improve the quality of the pictures.

Reviewer #2 (Significance):

These findings have important preclinical implications, since the study proposes a biomarker of resistance (profibrotic signature) and importantly, a potential new therapy to delay MAPKi resistance in melanoma (BIBF). It could also apply to other BRAFmutant cancers and diseases cursing with fibrosis.

Field of expertise: melanoma, drug resistance, cytoskeleton

Reviewer #3:

Major comments:

The manuscript is well written, data are convincing, well presented and supportive of the conclusions.

We thank the reviewer for his/her interest about our study and supportive comments.

Minor points that may be improved:

- The expression of miR-143/145 increases in melanoma cell lines treated with BRAFi and/or MEKi for 72h (Fig. 2B, Supp. Fig. 2B-F), and also after the development of resistance to MAPK-targeted therapies (Fig. 2A, Supp. Fig. 2A). The transient overexpression of miRs in therapy-naive cells leads to cells de-differentiation toward a mesenchymal/MAPK resistant state. On the other hand, these cells become more sensitive to BRAFi treatment when combined with LNA-mediated inhibition of miRs activity. It would be important to determine if the same occurs also in resistant cells, or whether MAPKi-resistance is established, cells are no longer sensitive to miRs blockade.

The answer to this point is common to the point 2 raised by the reviewer #2.
According to reviewers suggestion, we have conducted new experiments in resistant melanoma cells using different approaches to silence simultaneously the 2 mature miRNAs: i) an ASO-directed RNAse H degradation of the miR-143/145 precursor, as described by Plaisance *et al.,* JACC Basic Transl Sci. 2016, 1:472-493 to knock-down the pri-miRNA in cardiomyocytes, and ii) a combination of the 2 anti-miRs ASOs. Unfortunately, the first approach failed to efficiently inhibit the expression of mature miR-143-3p and miR-145-5, suggesting that the miR-143/145 cluster has a different precursor gene in melanoma than the one described in cardiomyocytes.
Concerning the second approach, as expected, the 2 anti-miRs ASOs as well as the combination of the 2 ASOs efficiently targeted the mature miRNAs (Supplementary Fig.S6C). Inhibition of miR-145-5p alone and combined inhibition of the two miRNAs significantly affected the viability of BRAFi resistant melanoma cells (M238R) in the absence of BRAFi (new Supplementary Fig.S6D) in a similar way as BIBF (Fig. 1J).

- In 2 out of 4 melanoma PDX samples naïve/resistant to combo BRAFi/MEKi therapy, the expression level of miR-143/145 cluster correlates with the de-differentiated transcriptomic profile of resistant tumor. How is Fascin1 expression in these samples?

The reviewer legitimately asks about the expression level of the miR-143/-145 target FSCN1 in the PDX samples used in the study. Expression of FSCN1 in PDX resistant vs naïve samples has been assessed by RT-qPCR. Results are presented below. We observed decreased expression of FSCN1 in only 1 out of the 2 samples showing increased miR-143/145 expression. This can be due to the heterogeneity of the subpopulations composing the tumor sample. It would have been interesting and probably more informative to test FSCN1 expression also at protein level since often miRNA molecular targets are inhibited at translation

level but unfortunately we did not have the access to protein extracts corresponding to these samples.

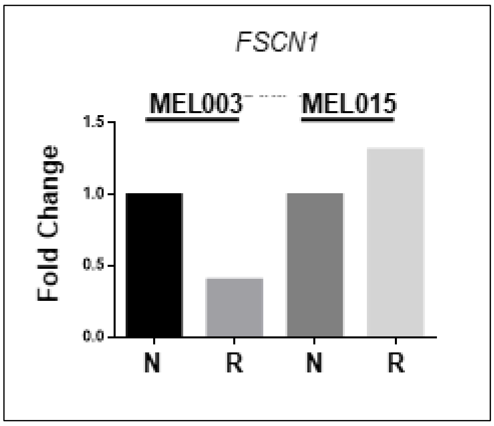

Relative FSCN1 expression levels were quantified in therapy-naïve (N) and resistant (R) PDX samples by RT-qPCR.

- The clinical relevance of the data could be strongly improved by assessing the expression of the miRs cluster and of its target Fascin1 in resistant subsets of patients, comparing their expression to patients before treatment, making use of available datasets.

We agree with the reviewer about the importance of providing clinical data supporting our observations. We have carefully analyzed all available profiling studies and datasets and provide below a summary.

Study	Results	Number of samples	Technique	GEO access nb
Song et al. 2017, Cancer Discovery	• MIR143HG up-expressed gene in MAPK-redundant R-lines (Rr)	• 18 isogenic P-lines versus R-lines (SDR or DDR) ;	RNA-seq platform in table S1B	GSE75313
	• MIR143HG in the Rr specific signature	• Patient-matched melanoma tumors (total n = 46) before MAPKi therapies (BRAFi, MEKi, or BRAFi + MEKi) at baseline (n = 20) and On-Tx (n = 26; 23 of 26 biopsies on day 22 or earlier) after objective clinical response		
Tsoi et al. 2018 Cancer Cell	• No MIR143HG expression in the differential expressed genes	53 human melanoma cell lines including paired acquired resistance sublines, established from patient biopsies	Illumina HiSeq2500 (GPL16791)	• RNASeq of melanoma cell lines GSE80829
	• FSCN1 FPKM downregulated in several acquired resistant cell lines (M229AR, M238AR, M376AR, M395AR, M397AR)			• RNASeq of vemu treatment timecourse GSE110054
Verfaillie et al. 2015 Nature Communications	• No MIR143HG expression detected in the dataset	11 short-term cultures derived from patient biopsies	Illumina HiSeq2000 (GPL11154)	GSE60666
	• FSCN1 not linked to the proliferative or invasive phenotype			
Hugo et al. 2015 Cell	•No miR-143, miR-145 or MIR143HG expression in the dataset	90 specimens of patient-derived melanoma tissues	Illumina HiSeq 2000 (Homo sapiens) GPL11154	GSE65186
Tirosh et al. 2016 Science	• MIR143HG upregulated in 4 out of 6 patients post MAPKi (bulk RNA seq)	• 6 patients pre and post MAPKi (Bulk RNA seq)	Illumina HiSeq 2500 (GPL16791)	GSE77940 (bulk RNA seq)
	• FSCN1 downregulated in 2 out of 6 patients post MAPKi (bulk RNA-seq)	• scRNA-seq data performed on stromal microenvironment only (FACS sorting)	Illumina NextSeq 500 (GPL18573)	GSE72056 (scRNA-seq)

List and summary of main available transcriptomic studies.

Overall, these studies have several limitations: i) as demonstrated in our study, expression of the miRNA cluster is specifically induced in response to therapy and is not present (or barely) in tumors at diagnosis; ii) no small RNA-seq datasets are available yet; iii) melanoma tumors are highly heterogeneous and invaded with stroma, especially CAFs and vessels that also express these miRNAs. We have looked at the expression of the MIR143HG precursor in these datasets and it was not present, probably due to low to medium sequencing depths in these clinical studies.

We have also carefully explored TCGA datasets to look at possible association between prognosis and mature / precursor miRNA as well as miRNA target (FSCN1) expression in skin cutaneous melanoma (SKCM) using the tools developed by Anaya *et al.* 2016 PeerJ Computer Science 2:e67. Cox regression models and Kaplan-Meier analysis (using different percentiles) did not show any association of our candidates with survival on a cohort of 459 SKCM patients (median survival of 2.4 years, see Kaplan plots below).

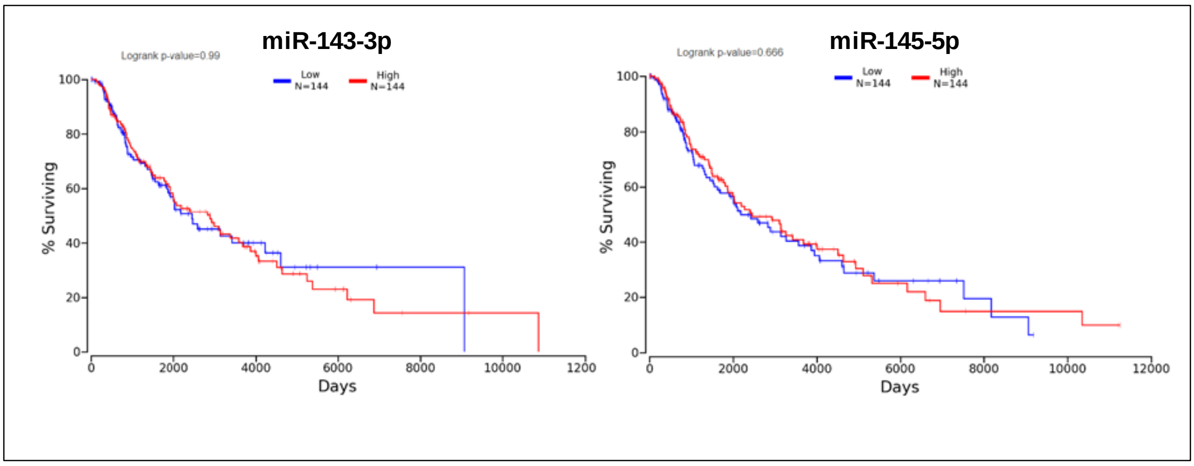

Kaplan–Meier analyses of correlations between miR-143-3p and miR-145-5p expression level and overall survival of TCGA SKCM patients. P-values were calculated using the logrank test.

Finally, during the revision process, we could have access to 9 relapsed melanoma for research purposes from the Dermatology Department of Nice University Hospital (CHU) following treatment with targeted therapies, immunotherapies or a combination of them. We analyzed in these samples the expression of fibrotic/mesenchymal genes, FSCN1 and the miR-143/145 cluster compared to the mean expression of the same genes/miRNAs in therapy naïve patient-derived xenografts (MEL003, MEL006, MEL015, MEL047). Our results indicate that relapsed tumors acquire a strong fibrotic signature which is associated to increased expression of the miR-143/145 cluster and decreased expression of FSCN1 (8 out of 9 patients) (see Results for referees below).

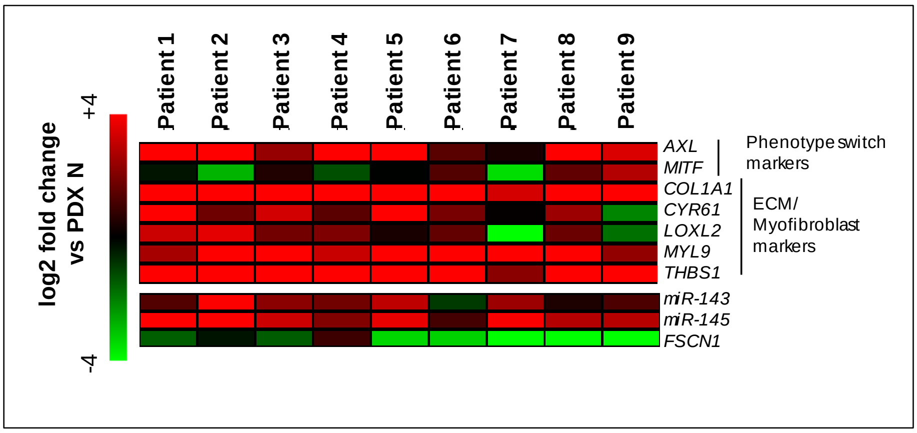

Expression of miR-143-3p, miR-145-5p, FSCN1 and mesenchymal markers in biopsies from melanoma patients progressing on therapies. Relative gene expression and miRNA expression levels were quantified by RT-qPCR.

This represents a good indicator for further clinical validation but is not solid enough to be incorporated in the manuscript. Overall, validation of our hypotheses in patient samples would require an entire new and highly complex clinical study comparing tumors at diagnosis with

relapsed tumors after targeted therapies and ideally processed using single-cell RNA-seq and/or RNA FISH to take into account the stromal compartment.

Minor comments:

- Fig. 4C, lower legend: M238P not M238S.

We apologize for this mistake and corrected it.

Reviewer #3 (Significance):

Nature and significance of the advances:

The findings not only suggest the combination therapy with the anti-fibrotic drug Nintedanib to be effective in enhancing MAPKi treatment in melanoma, reducing the development of resistance, but identify the molecular mechanism via the induction o the miR-143/145 cluster and the effects on the target Fascin1.

Compare to existing knowledge

These two miRNAs have been shown to have both oncogenic and oncosuppressor activities and have already been involved in EMT induction. The findings add yet one more piece to the puzzle.

Audience

This manuscript is not only of interest for oncology researchers but also of general interest or the understanding of fundamental biological processes and their effects on cancer therapy.

Your expertise

Molecular biologist and cancer research, transcriptional control of tumor transfromatin and progression including EMT, microRNAs -143/145

12th Nov 2021

Dear Dr. Mari,

Thank you for the submission of your manuscript to EMBO Molecular Medicine. We have now received the enclosed reports from the three referees who had initially reviewed your manuscript for Review Commons.
As you will see, they are supportive of publication, and I am therefore pleased to inform you that we will be able to accept your manuscript, once the following minor points will be addressed:

1/ Please include the statistical analysis of cell cycle in figure S4C-I as suggested by referee #3.

2/ Main manuscript text
- Please address the queries from our data editors in track changes mode in the attached manuscript file. Please use this file for any further modification and only keep in track changes mode the new modifications.
- If possible, please reduce the size of your title and shorten your abstract.
- Please remove the yellow highlights.
- Please provide up to 5 keywords.
- Please replace "Competing interests" by "Conflict of interest" and place it after author contributions.
- Material and methods:
o Include in the main manuscript files the methods currently listed in the supplementary file.
o In vivo experiments: please indicate the origin, gender and age of the mice, as well as the housing and husbandry conditions.
o Human samples: Please include a full statement that the experiments conformed to the principles set out in the WMA Declaration of Helsinki and the Department of Health and Human Services Belmont Report. This information should also be indicated in the checklist.
- Funding information should be merged with the acknowledgements. Fondation ARC is missing from our submission system.
- Data Availability: This section should be placed after the Material and Methods. Please note that the datasets must be publicly available before acceptance of the manuscript. Please remove "All other data are available in the main text or in the supplementary materials."
- References should be alphabetical and list 10 authors before et al.

3/ Figures:
- Please indicate in the figures or in their legends and in the Appendix the exact p= values, not a range (including for non-significant, ns). Some people found that to keep the figures clear, providing a supplemental table with all exact p-values was preferable. You are welcome to do this if you want to.
- Please provide Individual production quality figure files as .eps, .tif, .jpg (one file per figure).
- Appendix: please merge your different supplementary files in one appendix file with a table of content. The figures should be renamed "Appendix Figure S1" etc. and "Appendix Table S1" etc. Please reformat the tables for better legibility.

4/ We would also encourage you to include the source data for figure panels that show essential data. Numerical data should be provided as individual .xls or .csv files (including a tab describing the data). For blots or microscopy, uncropped images should be submitted (using a zip archive if multiple images need to be supplied for one panel). Additional information on source data and instruction on how to label the files are available at
.

5/ The paper explained: EMBO Molecular Medicine articles are accompanied by a summary of the articles to emphasize the major findings in the paper and their medical implications for the non-specialist reader. Please provide a draft summary of your article highlighting
- the medical issue you are addressing,
- the results obtained and
- their clinical impact.
This may be edited to ensure that readers understand the significance and context of the research. Please refer to any of our published articles for an example.

6/ For more information: There is space at the end of each article to list relevant web links for further consultation by our readers. Could you identify some relevant ones and provide such information as well? Some examples are patient associations, relevant databases, OMIM/proteins/genes links, author's websites, etc...

7/ Every published paper now includes a 'Synopsis' to further enhance discoverability. Synopses are displayed on the journal webpage and are freely accessible to all readers. They include a short stand first (maximum of 300 characters, including space) as well as 2-5 one-sentences bullet points that summarizes the paper. Please write the bullet points to summarize the key NEW

findings. They should be designed to be complementary to the abstract - i.e. not repeat the same text. We encourage inclusion of key acronyms and quantitative information (maximum of 30 words / bullet point). Please use the passive voice.

Please also suggest a striking image or visual abstract to illustrate your article as a PNG file 550 px wide x 300-600 px high.

8/ As part of the EMBO Publications transparent editorial process initiative (see our Editorial at http://embomolmed.embopress.org/content/2/9/329), EMBO Molecular Medicine will publish online a Review Process File (RPF) to accompany accepted manuscripts.
This file will be published in conjunction with your paper and will include the anonymous referee reports, your point-by-point response and all pertinent correspondence relating to the manuscript. Let us know whether you agree with the publication of the RPF and as here, IF YOU WANT TO REMOVE OR NOT any figures from it prior to publication.
Please note that the Authors checklist will be published at the end of the RPF.

I look forward to receiving your revised manuscript.

Yours sincerely,

Lise Roth

Lise Roth, PhD
Editor
EMBO Molecular Medicine

You can submit your revised files by logging onto our online manuscript tracking system or simply follow this link:

Link Not Available

Please do not share this URL as it will give anyone who clicks it access to your account.

***** Reviewer's comments *****

Referee #1 (Remarks for Author):

The authors have addressed the concerns raised. Thanks.

Referee #2 (Comments on Novelty/Model System for Author):

The authors extensively addressed the points raised, introducing new interesting results in the MS. Despite their efforts, the clinical relevance of their data was only slightly improved, mainly due to the paucity and technical limitations of the available clinical data. Overall, I believe that this was not the main scope of the present manuscript that still represents an interesting and well performed work. Therefore, I recommend the publication of the MS in its present form in EMBO Molecular Medicine.

Referee #3 (Remarks for Author):

Authors have done a thorough job of responding to my and other reviewer's concerns of the previous version. I fully support publication and congratulate the authors, this is an important study for the melanoma therapy resistance field and also with possible implications for other MAPK-driven cancers and fibrosis-related diseases.

For S4C- I would include in the figure the statistical analysis of cell cycle that is shown in point-by-point letter, page 9, panel B.

The authors performed the requested editorial changes.

10th Jan 2022

Dear Dr. Mari,

Thank you for sending the corrected manuscript text. I am pleased to inform you that your manuscript is accepted for publication and is now being sent to our publisher to be included in the next available issue of EMBO Molecular Medicine.

Please read below for additional IMPORTANT information regarding your article, its publication and the production process.

Congratulations on your nice work!

With my best wishes,

Lise

Lise Roth, Ph.D
Editor
EMBO Molecular Medicine

Follow us on Twitter @EmboMolMed
Sign up for eTOCs at embopress.org/alertsfeeds

EMBO PRESS

YOU MUST COMPLETE ALL CELLS WITH A PINK BACKGROUND ↓

PLEASE NOTE THAT THIS CHECKLIST WILL BE PUBLISHED ALONGSIDE YOUR PAPER

Corresponding Author Name: Bernard MARI
Journal Submitted to: EMBO MOL MED
Manuscript Number: EMM-2021-15295

Reporting Checklist For Life Sciences Articles (Rev. June 2017)

This checklist is used to ensure good reporting standards and to improve the reproducibility of published results. These guidelines are consistent with the Principles and Guidelines for Reporting Preclinical Research issued by the NIH in 2014. Please follow the journal's authorship guidelines in preparing your manuscript.

A- Figures
1. Data
The data shown in figures should satisfy the following conditions:

- the data were obtained and processed according to the field's best practice and are presented to reflect the results of the experiments in an accurate and unbiased manner.
- figure panels include only data points, measurements or observations that can be compared to each other in a scientifically meaningful way.
- graphs include clearly labeled error bars for independent experiments and sample sizes. Unless justified, error bars should not be shown for technical replicates.
- if n< 5, the individual data points from each experiment should be plotted and any statistical test employed should be justified
- Source Data should be included to report the data underlying graphs. Please follow the guidelines set out in the author ship guidelines on Data Presentation.

2. Captions
Each figure caption should contain the following information, for each panel where they are relevant:

- a specification of the experimental system investigated (eg cell line, species name).
- the assay(s) and method(s) used to carry out the reported observations and measurements
- an explicit mention of the biological and chemical entity(ies) that are being measured.
- an explicit mention of the biological and chemical entity(ies) that are altered/varied/perturbed in a controlled manner.

- the exact sample size (n) for each experimental group/condition, given as a number, not a range;
- a description of the sample collection allowing the reader to understand whether the samples represent technical or biological replicates (including how many animals, litters, cultures, etc.).
- a statement of how many times the experiment shown was independently replicated in the laboratory.
- definitions of statistical methods and measures:
  - common tests, such as t-test (please specify whether paired vs. unpaired), simple χ2 tests, Wilcoxon and Mann-Whitney tests, can be unambiguously identified by name only, but more complex techniques should be described in the methods section;
  - are tests one-sided or two-sided?
  - are there adjustments for multiple comparisons?
  - exact statistical test results, e.g., P values = x but not P values < x;
  - definition of 'center values' as median or average;
  - definition of error bars as s.d. or s.e.m.

Any descriptions too long for the figure legend should be included in the methods section and/or with the source data.

In the pink boxes below, please ensure that the answers to the following questions are reported in the manuscript itself. Every question should be answered. If the question is not relevant to your research, please write NA (non applicable). We encourage you to include a specific subsection in the methods section for statistics, reagents, animal models and human subjects.

USEFUL LINKS FOR COMPLETING THIS FORM

http://www.antibodypedia.com
http://1degreebio.org
http://www.equator-network.org/reporting-guidelines/improving-bioscience-research-repor
http://grants.nih.gov/grants/olaw/olaw.htm
http://www.mrc.ac.uk/Ourresearch/Ethicsresearchguidance/Useofanimals/index.htm
http://ClinicalTrials.gov
http://www.consort-statement.org
http://www.consort-statement.org/checklists/view/32-consort/66-title
http://www.equator-network.org/reporting-guidelines/reporting-recommendations-for-tumo
http://datadryad.org
http://figshare.com
http://www.ncbi.nlm.nih.gov/gap
http://www.ebi.ac.uk/ega
http://biomodels.net/
http://biomodels.net/miriam/
http://jjj.biochem.sun.ac.za
http://oba.od.nih.gov/biosecurity/biosecurity_documents.html
http://www.selectagents.gov/

B- Statistics and general methods

Please fill out these boxes ↓ (Do not worry if you cannot see all your text once you press return)

1.a. How was the sample size chosen to ensure adequate power to detect a pre-specified effect size?	Sample size was chosen empirically based on our previous experiences in the calculation of experimental variability. For all in vitro experiments, a minimum of 3 biological replicates were performed, while 6 biological replicates were performed for in vivo experiments.
1.b. For animal studies, include a statement about sample size estimate even if no statistical methods were used.	No statistical method was used to predetermine sample size. Sample size was chosen empirically based on our previous experiences in the calculation of experimental variability (group sizes of 6 mice)
2. Describe inclusion/exclusion criteria if samples or animals were excluded from the analysis. Were the criteria pre-established?	No samples, mice or data points were excluded from the reported analyses.
3. Were any steps taken to minimize the effects of subjective bias when allocating animals/samples to treatment (e.g. randomization procedure)? If yes, please describe.	After tumor cells injection, animals were randomly allocated to pre-defined experimental groups.
For animal studies, include a statement about randomization even if no randomization was used.	Randomisation of mice was performed blindly by a lab member not involved in the study.
4.a. Were any steps taken to minimize the effects of subjective bias during group allocation or/and when assessing results (e.g. blinding of the investigator)? If yes please describe.	Measurements of tumor volumes were performed by an investigator blind to the group allocation of mice.
4.b. For animal studies, include a statement about blinding even if no blinding was done	Blinding was performed by a lab member not involved in the study.
5. For every figure, are statistical tests justified as appropriate?	For every figure, statistical tests are justified as appropriate. See methods ''Statistical analysis'' and individual figure legends.
Do the data meet the assumptions of the tests (e.g., normal distribution)? Describe any methods used to assess it.	When possible, assumption of normality was tested using Shapiro Wilk's W test.
Is there an estimate of variation within each group of data?	Variation was reported in ANOVA output as Sum of square within each group of data.

Is the variance similar between the groups that are being statistically compared?	Variance were found to be similar between groups statistically compared when parametric tests were used.

C- Reagents

6. To show that antibodies were profiled for use in the system under study (assay and species), provide a citation, catalog number and/or clone number, supplementary information or reference to an antibody validation profile. e.g., Antibodypedia (see link list at top right), 1DegreeBio (see link list at top right).	Company, catalog number and dilution uses are provided for each antobody: see supplemental Table S6.
7. Identify the source of cell lines and report if they were recently authenticated (e.g., by STR profiling) and tested for mycoplasma contamination.	The source of cell lines was specified in the Materials and Methods section. Cell lines were STR profiled and confirmed to be authentic. All cell lines were tested and found to be mycoplasma-negative.

* for all hyperlinks, please see the table at the top right of the document

D- Animal Models

8. Report species, strain, gender, age of animals and genetic modification status where applicable. Please detail housing and husbandry conditions and the source of animals.	In vivo experiments were performed on 6 weeks old female C57BL/6 mice. Animal housing was carried out in the Centre Méditerranéen de Médecine Moléculaire in accordance with the Institutional Animal Care and the local ethical committee (CIEPAL-Azur). Mice were from Janvier Labs (https://www.janvier-labs.com/)
9. For experiments involving live vertebrates, include a statement of compliance with ethical regulations and identify the committee(s) approving the experiments.	All mouse experiments were carried out in accordance with the guidelines of the Institutional Animal Care and the local ethical committee and within the context of approved project applications (CIEPAL-Azur agreement NCE/2018-483).
10. We recommend consulting the ARRIVE guidelines (see link list at top right) (PLoS Biol. 8(6), e1000412, 2010) to ensure that other relevant aspects of animal studies are adequately reported. See author guidelines, under 'Reporting Guidelines'. See also: NIH (see link list at top right) and MRC (see link list at top right) recommendations. Please confirm compliance.	We confirm compliance.

E- Human Subjects

11. Identify the committee(s) approving the study protocol.	UZ Leuven Medical Ethical Committee (S54185/S57760/S59199)
12. Include a statement confirming that informed consent was obtained from all subjects and that the experiments conformed to the principles set out in the WMA Declaration of Helsinki and the Department of Health and Human Services Belmont Report.	Written informed consent was obtained from all patients. Experiments conformed to the principles set out in the WMA Declaration of Helsinki and the Department of Health and Human Services Belmont Report.
13. For publication of patient photos, include a statement confirming that consent to publish was obtained.	N/A
14. Report any restrictions on the availability (and/or on the use) of human data or samples.	N/A
15. Report the clinical trial registration number (at ClinicalTrials.gov or equivalent), where applicable.	N/A
16. For phase II and III randomized controlled trials, please refer to the CONSORT flow diagram (see link list at top right) and submit the CONSORT checklist (see link list at top right) with your submission. See author guidelines, under 'Reporting Guidelines'. Please confirm you have submitted this list.	N/A
17. For tumor marker prognostic studies, we recommend that you follow the REMARK reporting guidelines (see link list at top right). See author guidelines, under 'Reporting Guidelines'. Please confirm you have followed these guidelines.	N/A

F- Data Accessibility

18: Provide a "Data Availability" section at the end of the Materials & Methods, listing the accession codes for data generated in this study and deposited in a public database (e.g. RNA-Seq data: Gene Expression Omnibus GSE39462, Proteomics data: PRIDE PXD000208 etc.) Please refer to our author guidelines for 'Data Deposition'.<br><br>Data deposition in a public repository is mandatory for:<br>a. Protein, DNA and RNA sequences<br>b. Macromolecular structures<br>c. Crystallographic data for small molecules<br>d. Functional genomics data<br>e. Proteomics and molecular interactions	A "Data Availability" section has been provided with all expression dataset records deposited in Gene Expression Omnibus (GEO): SuperSerie record GSE171883 containing 3 distinct datasets.
19. Deposition is strongly recommended for any datasets that are central and integral to the study; please consider the journal's data policy. If no structured public repository exists for a given data type, we encourage the provision of datasets in the manuscript as a Supplementary Document (see author guidelines under 'Expanded View' or in unstructured repositories such as Dryad (see link list at top right) or Figshare (see link list at top right).	N/A
20. Access to human clinical and genomic datasets should be provided with as few restrictions as possible while respecting ethical obligations to the patients and relevant medical and legal issues. If practically possible and compatible with the individual consent agreement used in the study, such data should be deposited in one of the major public access-controlled repositories such as dbGAP (see link list at top right) or EGA (see link list at top right).	N/A
21. Computational models that are central and integral to a study should be shared without restrictions and provided in a machine-readable form. The relevant accession numbers or links should be provided. When possible, standardized format (SBML, CellML) should be used instead of scripts (e.g. MATLAB). Authors are strongly encouraged to follow the MIRIAM guidelines (see link list at top right) and deposit their model in a public database such as Biomodels (see link list at top right) or JWS Online (see link list at top right). If computer source code is provided with the paper, it should be deposited in a public repository or included in supplementary information.	N/A

G- Dual use research of concern

22. Could your study fall under dual use research restrictions? Please check biosecurity documents (see link list at top right) and list of select agents and toxins (APHIS/CDC) (see link list at top right). According to our biosecurity guidelines, provide a statement only if it could.	N/A
